# A Genome-Wide Survey of Highly Expressed Non-Coding RNAs and Biological Validation of Selected Candidates in *Agrobacterium tumefaciens*

**DOI:** 10.1371/journal.pone.0070720

**Published:** 2013-08-08

**Authors:** Keunsub Lee, Xiaoqiu Huang, Chichun Yang, Danny Lee, Vincent Ho, Kan Nobuta, Jian-Bing Fan, Kan Wang

**Affiliations:** 1 Center for Plant Transformation, Plant Sciences Institute, Iowa State University, Ames, Iowa, United States of America; 2 Department of Agronomy, Iowa State University, Ames, Iowa, United States of America; 3 Department of Computer Science, Iowa State University, Ames, Iowa, United States of America; 4 Scientific Research, Illumina Inc., San Diego, California, United States of America; Cinvestav, Mexico

## Abstract

*Agrobacterium tumefaciens* is a plant pathogen that has the natural ability of delivering and integrating a piece of its own DNA into plant genome. Although bacterial non-coding RNAs (ncRNAs) have been shown to regulate various biological processes including virulence, we have limited knowledge of how *Agrobacterium* ncRNAs regulate this unique inter-Kingdom gene transfer. Using whole transcriptome sequencing and an ncRNA search algorithm developed for this work, we identified 475 highly expressed candidate ncRNAs from *A. tumefaciens* C58, including 101 *trans*-encoded small RNAs (sRNAs), 354 antisense RNAs (asRNAs), 20 5′ untranslated region (UTR) leaders including a RNA thermosensor and 6 riboswitches. Moreover, transcription start site (TSS) mapping analysis revealed that about 51% of the mapped mRNAs have 5′ UTRs longer than 60 nt, suggesting that numerous *cis*-acting regulatory elements might be encoded in the *A. tumefaciens* genome. Eighteen asRNAs were found on the complementary strands of *virA*, *virB*, *virC*, *virD*, and *virE* operons. Fifteen ncRNAs were induced and 7 were suppressed by the *Agrobacterium* virulence (*vir*) gene inducer acetosyringone (AS), a phenolic compound secreted by the plants. Interestingly, fourteen of the AS-induced ncRNAs have putative *vir* box sequences in the upstream regions. We experimentally validated expression of 36 ncRNAs using Northern blot and Rapid Amplification of cDNA Ends analyses. We show functional relevance of two 5′ UTR elements: a RNA thermonsensor (C1_109596F) that may regulate translation of the major cold shock protein *cspA*, and a thi-box riboswitch (C1_2541934R) that may transcriptionally regulate a thiamine biosynthesis operon, *thiCOGG*. Further studies on ncRNAs functions in this bacterium may provide insights and strategies that can be used to better manage pathogenic bacteria for plants and to improve *Agrobacterum*-mediated plant transformation.

## Introduction

Last decade, non-coding RNAs (ncRNAs) have emerged as crucial regulators of diverse physiological and cellular processes in both bacteria and eukaryotes. In bacteria, ncRNAs control various biological processes including, but not limited to, virulence [1], photosynthesis [2], iron homeostasis [3], pH sensing [4], temperature sensing [5], plasmid replication [6]–[9], and toxin suppressions [10]–[12]. Bacterial ncRNAs include ribosomal RNAs (rRNAs), transfer RNAs (tRNAs), *cis*-encoded antisense RNAs (asRNAs) and *trans*-encoded small RNAs (sRNAs). rRNAs and tRNAs regulate protein translation and some tRNAs have also been implicated to have regulatory roles [13], [14]. AsRNAs regulate genes encoded on the opposite strand, while sRNAs have their gene targets encoded elsewhere in the genome and often have multiple targets 15,16. Both asRNAs and sRNAs base-pair with target mRNAs and alter translation and/or mRNA stability. Most of the time they inhibit translation and lead to co-degradation [17].

Various strategies can be used to identify ncRNAs in bacteria. Computational predictions followed by experimental validations have revealed many sRNAs in the earlier days [18]–[20]. Sequencing cDNA clones prepared from small-sized RNA fractions can be useful to identify sRNAs [21]. Most recently, tiling arrays and deep sequencing analyses have identified tens to hundreds of ncRNAs from various bacterial species [22]–[25]. However, it remains difficult to answer how many regulatory ncRNAs exist in any bacterial genome [17] due to several major challenges: 1) extremely abundant ribosomal RNAs, which account for 95–97% of total RNA, reduce detection sensitivity for transcriptome analysis [26], 2) some ncRNAs are only expressed under specific conditions, and 3) ncRNAs derived from mRNAs are hard to be distinguished. The presence of internal transcriptional start site (TSS) within annotated ORFs also adds another level of complexity to the transcriptome [27]. In addition, due to the huge data size generated by deep sequencing, often in hundreds of gigabytes (GB), differences in the algorithms for data analysis affect the outcomes [28]. Thus, more robust sequencing technology and advances in data analysis will likely expand the inventories of ncRNAs, and ultimately help us to see better insights into the gene regulatory networks.

*A. tumefaciens* is a gram-negative bacterium and the causal agent of crown gall disease. *A. tumefaciens* recognizes various signals from plants and convert its life style from a free-living saprobe to a plant pathogen. The way *A. tumefaciens* affects infected plants is unique: it genetically transform host plant by exporting a segment of tumor inducing (Ti) plasmid (T-DNA) into plant cell where the T-DNA integrates into host plant genome [29]. Genes encoded by the T-DNA are expressed to produce plant hormones to induce tumor formation and enzymes to make novel compounds called opines that are utilized by *A. tumefaciens* [29]. The transition from a saprobe to a pathogen requires expression of virulence genes on Ti plasmid and some other genes encoded on the chromosome [30]. This process begins with recognition of plant-produced compounds, such as phenolics (e.g., acetosyringone) and sugars, by the *Agrobacterium* VirA two-component sensor kinase in accordance with a periplasmic sugar binding protein ChvE encoded by the chromosomal virulence gene *E* (*chvE*) [31]–[33]. VirA phosphorylates VirG, the two-component response regulator, which activates transcription of other virulence genes [34], [35]. While much is known about how the T-DNA border sequences, its virulence genes and some plant genes are involved in this unique inter-Kingdom gene transfer [29], the importance of regulatory functions of ncRNAs in that process remains unanswered. In various bacteria, some ncRNAs have been found to be crucial virulence factors [1]; similar mechanisms may exist in plant pathogenic bacteria, such as *A. tumefaciens*.

Several transcriptome analyses using microarrays have identified differentially expressed genes in *A. tumefaciens* in response to various conditions, such as low pH [36], plant-derived signaling molecules [37], and the loss of phosphatidylcholine biosynthesis [38]. A few recent studies showed that *A. tumefaciens* uses ncRNAs to regulate important biological processes, such as Ti plasmid replication [39] and the uptake of a plant-derived signaling molecule, γ-amynobutyric acid (GABA) by an ABC transporter [40]. The RNA chaperone Hfq was involved in the sRNA-mediated regulation of the GABA transporter, and the *hfq* deletion mutant showed reduced virulence, suggesting that ncRNAs may play important roles during *Agrobacterium*-plant interactions [41]. A recent deep sequencing study by Wilms et al [51] obtained a total of 348,998 cDNA reads (≥18 nt) from four cDNA libraries and reported 152 sRNAs and 76 asRNAs from *A. tumefaciens* C58. Some of these were induced by the phenolic compound (AS), which is essential for the induction of *Agrobacterium* virulence genes [42].

In this study, we present an extensive list of ncRNAs for the same strain *A. tumefaciens* C58. We improved RNA-seq detection sensitivity by using a combination of treatments, including the MICROB*Express* ^TM^ kit and 5′-phosphate-dependent exonuclease to deplete ribosomal RNAs. We then performed whole transcriptome sequencing (RNA-seq) on an Illumina GAII platform and obtained over 48 million uniquely mapped reads ( = 50 bp) from eight cDNA libraries. Using an ncRNA search algorithm developed for this work, we identified and generated a list of highly expressed ncRNAs in *Agrobacterium* strain C58 grown under 4 different conditions. Selected ncRNAs were experimentally validated by RACE and Northern analyses.

## Results and Discussion

### Overview of whole genome transcriptome sequencing

To identify *A. tumefaciens* ncRNAs expressed under different conditions, we grew the strain C58 and harvested the cultures at: 1) nutrient rich medium at mid-log phase (YEP-L: OD_600_  = 0.5), 2) nutrient rich medium at late stationary phase (YEP-S: OD_600_  = 1.3), 3) modified AB induction medium without AS (AB: OD_600_  = 0.8) and 4) modified AB induction medium with AS (IND: OD_600_  = 0.8). To improve non-rRNA detection sensitivity, we removed 16S and 23S rRNAs using two commercially available kits. First, total RNA sample was treated according to the MICROB*Express* ^TM^ kit (Ambion, USA), which uses hybridization oligonucleotides attached to magnetic beads to selectively deplete 16S and 23S rRNAs. Analysis by an Agilent 2100 BioAnalyzer showed that about 55% of 16S and 23S rRNAs had been successfully removed using this kit. Because *Agrobacterium* 23S rRNA undergoes post-transcriptional fragmentation [43], some 23S rRNA could not be removed by the hybridization oligonucleotides. The remaining rRNAs were further depleted using the terminator 5′-phosphate-dependent exonuclease (TEX), which selectively digests processed RNA molecules with 5′-monophosphate [44]. Because rRNAs are processed from the primary transcript upon transcription, this enzyme is useful to deplete rRNAs from total RNA samples as well as to enrich primary transcripts [26]. This dual treatment can increase the non-ribosomal RNA components from 3–5% to up to 25% in a total RNA population [26]. Thus, a total of eight cDNA libraries, YEP-L, YEP-S, AB and IND with/without primary transcript enrichment (−/+ TEX), were prepared and sequenced in parallel on the Illumina GAII platform.

We obtained a total of 842.109 million reads from the 8 cDNA libraries (Table 1; 429.340 million reads from the –TEX samples and 412.769 million reads from the +TEX samples). These short sequence reads (50 bp) were aligned to the reference genome (NC_003062.2, NC_003063.2, NC_003064.2, NC_003065.3) using the Bowtie 2 program [45]. A total of 490.552 million reads, 252.825 ( = 18,959+233,866) from the –TEX samples and 237.728 ( = 29,343+208,385) million reads from the +TEX samples, were mapped to the reference genome (Table 1). Among these, 92.5% of the reads (233.866 millions) from the –TEX samples and 87.7% of the reads (208.385 millions) from the +TEX samples were aligned more than once to the reference genome.

**Table 1:** Summary of RNA sequencing and alignment*.

		Number of reads (×1000)				
	TEX	YEP-L	YEP-S	AB	IND	Total
Total generated	−	106,658	102,249	112,547	107,886	429,340
	+	115,420	84,435	105,172	107,741	412,769
Total aligned	−	60,046	60,536	67,643	64,600	252,825
	+	61,617	53,185	62,453	60,473	237,727
**Aligned 1 time**	**−**	**4,033**	**3,534**	**5,760**	**5,632**	**18,959**
	**+**	**5,155**	**5,411**	**8,929**	**9,848**	**29,343**
Aligned >1 time	−	56,013	57,001	61,883	58,969	233,866
	+	56,462	47,774	53,524	50,625	208,385

* Short sequence reads were aligned to the *A. tumefaciens* C58 reference genome using the Bowtie 2 program [45].

The number of reads that were mapped exactly once to the reference genome (uniquely mapped reads, UMR) varied among different cDNA libraries, ranging from 3.534 million (YEP-S, – TEX) to 9.848 million (IND, +TEX). We used only these UMRs for gene expression quantification and subsequent analysis. A total of 18.959 million UMRs in the –TEX samples and 29.343 million UMRs in the +TEX samples were found, representing 7.5% (of the 252.825 million) and 12.3% (of the 237.727 million) of total mapped reads, respectively. Thus, a considerably higher percentage of UMRs were obtained in the +TEX libraries than the –TEX libraries, suggesting improved non-rRNA detection sensitivity.

The increased number of UMRs was likely due to the depletion of rRNAs and tRNAs by TEX. We computed the total number of UMRs that were aligned to the rRNAs and tRNAs. In the –TEX libraries, a total of 3.785 million reads were mapped to rRNAs and tRNAs, while in the +TEX libraries only 0.662 million reads were mapped to rRNAs and tRNAs. The percentage of the rRNAs and tRNAs reads were significantly decreased from 20.9% in the –TEX samples (YEP-L, 25.0; YEP-S, 26.0; AB, 16.3; IND, 16.3) to 2.3% in the +TEX samples (YEP-L, 3.5; YEP-S, 1.5; AB, 2.3; IND, 1.9).

The UMRs were then piled up using the SAMtools [46], which provides various tools to manipulate sequence alignment data. This pile-up step allowed us to compute the depth of coverage (i.e., the number of reads mapped to a nucleotide position on the forward or reverse strands) throughout the entire genome of *A. tumefaciens* C58. For gene expression quantification, we first computed the average depth of coverage (ADC) of each gene, and then converted ADC to RPKM (Reads per Kilobase per Million mapped reads) by a simple formula: 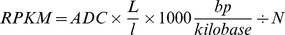, where *L* is the length of a gene, *l* is the length of a sequence read ( = 50 bp), and *N* is the number of total reads in millions [47].

Primary transcript enrichment using TEX was helpful to obtain relatively under-represented RNA species (Table 1; Figure S1A&B in File S2). As shown in Figure S1A and B, the *vir* genes expression was higher with TEX treatment than was without TEX treatment. To determine whether TEX treatment has systematic effects on gene expression quantification, we generated a scatter plot with the log-transformed RPKM values of all annotated protein-coding genes with and without TEX treatment (Figure S1C in File S2). Some genes had lower expressions with TEX treatment than were without TEX treatment (e.g., data points on the X-axis in Figure S1C in File S2). These could represent post-transcriptionally processed transcripts. However, TEX treatment did not have systematic biased effects on quantifying gene expression (Pearson's product-moment coefficient, *r* = 0.91; Figure S1C in File S2), and many genes became detectable after TEX treatments (i.e., data points on the Y-axis in Figure S1C in File S2). Indeed, among the 5432 protein-coding genes, 3411∼3842 genes were detected (RPKM >0) without TEX treatment (YEP-L, 3603; YEP-S, 3487; AB, 3842; IND, 3411), while 3957∼4361 genes were detected with TEX enrichment (YEP-L, 3957; YEP-S, 3959; AB, 4361; IND, 4156). These results suggest that combination of rRNA depletion kit (MICROB*Express* ^TM^ kit) and TEX treatment is very useful to improve overall RNA-seq detection sensitivity.

### Data validation

**Figure 1:**
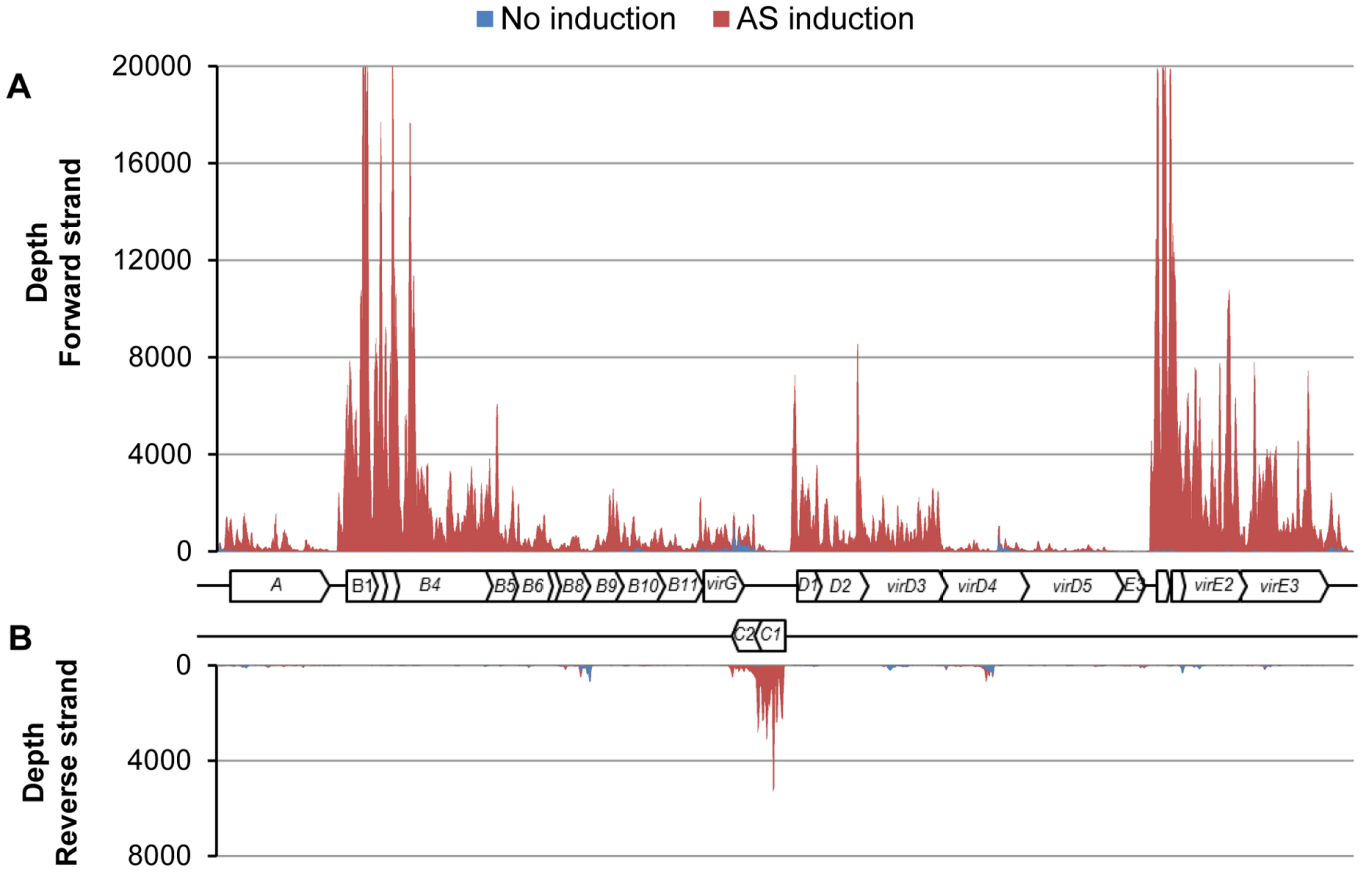
Induction of *vir* genes with AS. Expression of 24 *vir* genes with and without AS was visualized for data validation. Depth of coverage at each nucleotide position from 180,590 to 211,094 of Ti plasmid was plotted for (**A**) forward strand and (**B**) reverse strand. A total of 24 *vir* genes were included: *virA*, *virB* (B1∼B11), *virG*, *virC* (C1, C2), *virD* (D1∼D5) and *virE* (E0∼E3).

For validation purposes, we visualized *vir* genes expression on the Ti plasmid. We plotted the depth of coverage at each nucleotide position from 180,590 to 211,094 of Ti plasmid (Figure 1). As shown in Figure 1, there was a large difference in *vir* genes expression with/without AS induction. For instance, the two component sensor kinase *virA* was expressed at a low level without induction, 33 RPKM, but was expressed at 22.6 fold higher level with induction, 734 RPKM. Likewise, the expression levels of the two component response regulator *virG* were 219 RPKM without induction and 1614 RPKM with induction, a 7.4 fold increase. In a previous study by Winans et al. [48], a *lacZ* reporter assay demonstrated about 9-fold increase in *virA* expression by AS. In our RNA-seq study, *virA* transcript level was increased by a 22.6-fold. Although the fold changes were not equal (9 vs. 22.6), it has been well-documented that mRNA level is not directly correlated with protein abundance [49], [50]. Other *vir* operons, such as *virB*, *virC* and *virD* were only expressed under induction condition. These *vir* genes expression patterns were consistent with previous microarray and RNA-seq studies (Table S1 in File S1) [38], [51].

Interestingly, there were some noticeable antisense transcripts on the complementary strands of *virB9*, *virC*, *virD* and *virE*. The existence of some of these *cis*-antisense transcripts were confirmed by 5′ and 3′ RACE (Rapid Amplification of cDNA Ends) (Figure S2 in File S2). In addition, *virB10*, *virB11* and *virD4* had internal transcripts expressed without AS induction. Especially, *virD4* internal transcript (virD4*; pTi 201529–201869) was expressed under all four growth conditions (Figure S3 in File S2; RPKM: YEP-L, 267; YEP-S, 380; AB, 526; IND, 738), as opposed to the full length transcript, virD4, which is only expressed under *vir* gene induction conditions. Therefore, if virD4* has a functional role, if any, it may not be restricted to pathogenicity. The functional relevance of these RNAs needs to be determined, but the presence of these transcripts suggests that the *A. tumefaciens* transcriptome could be as complex as those of other bacterial transcriptomes such as *Listeria monocytogenes, Escherichia coli, and Sinorhizobium meliloti* [52], and the full inventory of transcripts, both protein-coding and non-coding, may be significantly expanded in the future.

### Transcriptional Start Sites (TSS) mapping

**Figure 2:**
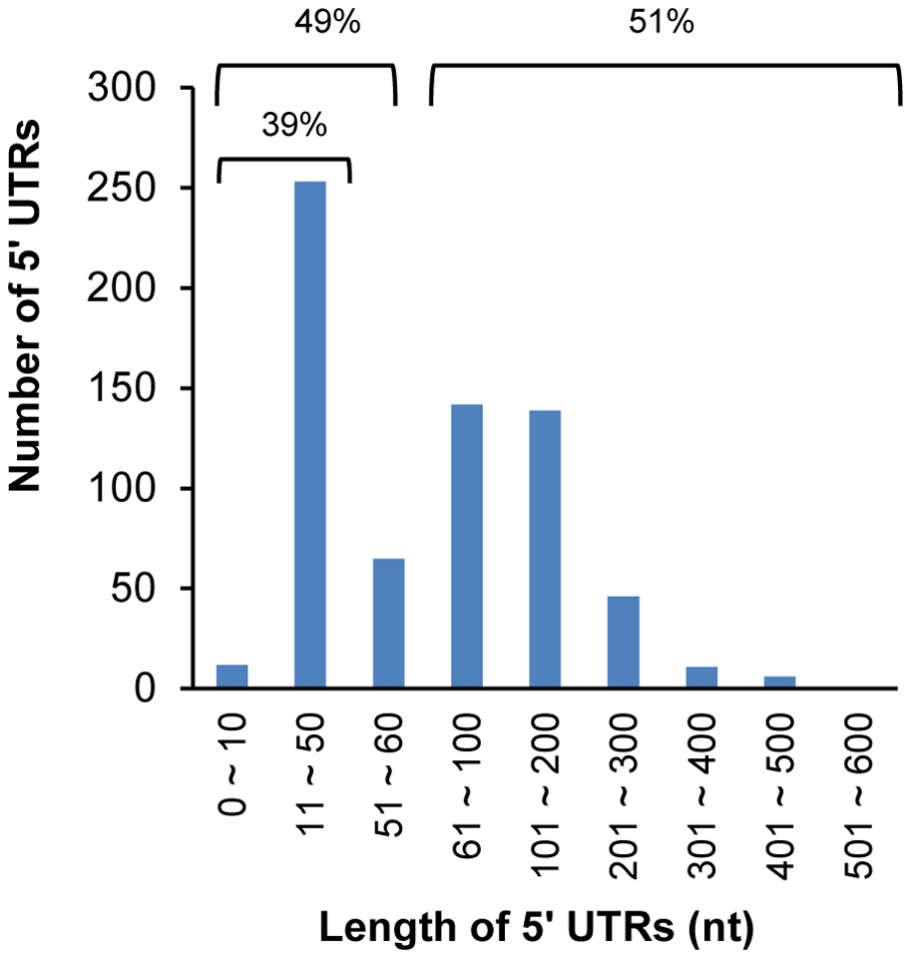
Variation of 5′ UTR length. The distance between TSS and start codon (5′ UTR) varied substantially from 0 to 521 nt, averaging 88 nt and median of 61 nt. Among the 675 protein coding genes, 1.8% (12) were leaderless (≤10 nt), 39% (253) were short (11∼50 nt), while 30% (203) were long (>100 nt). About 51% (345) of 5′ UTRs were longer than 60 nt.

We identified TSSs for 705 annotated protein-coding genes (Table S2 in File S1). Excluding 30 genes whose TSS were mapped within the coding region, we estimated the 5′ UTR lengths for 675 protein-coding genes. The length of the 5′ UTR varied from 0 to 521 nt, averaging 88 nt with a median of 61 nt (Figure 2). About 39% (253) of the protein-coding genes had short 5′ UTRs (≤50 nt), while 30% (203) of them had long 5′ UTRs (>100 nt). About 51% (345) had 5′ UTRs longer than 60 nt, which is long enough to contain *cis*-regulatory element [53]. There were 12 genes with 5′ UTR length no longer than 10 nt (Table S2 in File S1), suggesting that leaderless mRNAs exist in this bacterium, which may require special ribosomes for translation [54]. These results were comparable to those obtained by Wilms et al. [51]: the estimated length of 5′ UTRs reported in their study varied from 0 to 544 nt averaging 87 nt and about 40% (145/356) were short (≤50 nt).

We also found that at least 27 genes, 20 of them encoding hypothetical proteins (marked by * in Table S2 in File S1), had TSSs mapped within annotated coding sequences, suggesting that they might be incorrectly annotated. Indeed, BLAST searches against the GenBank database using the predicted amino acid sequences as queries showed that 19 of those 27 genes have longer N-termini than their homologs (Table S2 in File S1). Further investigation is required to verify these sequences.

### Identification of non-coding RNAs

To identify highly expressed ncRNA transcripts, we calculated depth of coverage at each nucleotide position on both forward and reverse strands of all four replicons of *A. tumefaciens*. Then, using already annotated gene features [55]–[57], we searched for non-gene-coding genomic regions that have at least 10 times higher depth of coverage than adjacent regions. This was done to avoid erroneous annotations due to pervasive transcription [58], [59]. This approach yielded a total of 475 candidate ncRNAs, 101 *trans*-encoded small RNAs (sRNAs), 354 antisense RNAs (asRNAs) and 20 5′ UTR elements.Some of these were differentially expressed under different growth conditions (Table 2; Table S3 in File S1). Candidate ncRNAs were distributed across all four replicons: 221 on the circular chromosome, 164 on the linear chromosome, 43 on the pAt plasmid and 47 on the Ti plasmid. The vast majority of the sRNAs (89/101) were found on the two chromosomes and only 12 of them were found on the two plasmids. In addition, 87% of ncRNAs (78/90) found on the two plasmids were asRNAs; 18 of them were encoded on the opposite strand of *virA*, *virB*, *virD*, *virE*, *virF* and *virK*.

**Table 2:** Distribution of ncRNAs on four replicons.

Replicon	sRNA	asRNA	5′ UTR	Total	%
Circular chromosome	56	154	11	221	46.5
Linear chromosome	33	125	6	164	34.5
At plasmid	8	33	2	43	9.1
Ti plasmid	4	42	1	47	9.9
**Total**	**101**	**354**	**20**	**475**	**100**
%	21.3	74.5	4.2	100.0	

We searched Rfam database (http://rfam.sanger.ac.uk/) and previously reported ncRNAs, and found that 91 of the 475 candidate ncRNAs (37 sRNAs and 44 asRNAs, and 10 5′ UTR elements) had been identified previously [39], [40], [51]. Those 91 ncRNAs correspond to 92 previously identified ncRNAs including recently identified *A. tumefaciens* sRNAs, repE [39], AbcR1 and AbcR2 [40]. Some well conserved sRNAs were also identified, such as 6S RNA, the signal recognition particle (SRP) RNA (4.5S RNA), tmRNA (SsrA, Atu2049), RNase P, and counter-transcribed RNA (ctRNA_p42d, Atu8080), which binds to repB mRNA to inhibit translation (Table S3 in File S1) [60]. The discrepancy (91 vs. 92) was because an ncRNA identified by our study (C1_1533961R) overlapped with 2 ncRNAs identified by Wilms et al. [51], 1533826–1533764 and 1533957–1533833. Thus, a total of 384 novel ncRNAs were identified in this study, including 64 sRNAs, 310 asRNAs, and 10 5′ UTR leaders.

A previous study by Wilms et al [51] used the Roche 454 platform to sequence the *A. tumefaciens* transcriptome and identified 228 candidate ncRNAs. They obtained a total of 348,998 cDNA reads (≥18 bp) mapped to the reference genomes from four libraries, representing two growth conditions (−Vir and +Vir). We used Illumina GAII platform and obtained a total of 2415 megabases (Mb) sequences from more than 48.3 million UMRs ( = 50 bp). In addition, we sequenced four more cDNA libraries representing two more growth conditions including stationary phase in a nutrient rich medium, under which many stress-related ncRNAs accumulate [17]. As summarized in Table 3, we categorized the candidate ncRNAs into three groups: sRNAs, asRNAs, and 5′ UTR leaders. Wilms et al. [51] originally reported 152 sRNAs and 76 asRNAs, but our study suggested that three sRNAs reported by Wilms et al were likely to be 5′ UTR leaders (Table 3). From our data set, we identified 101 sRNAs, 354 asRNAs and 20 5′ UTR leaders. Among those, 36 sRNAs, 44 asRNAs and three 5′ UTR leaders were identified by both studies (Table 3; Common). A total of 145 ncRNAs were identified only by Wilms et al. [51] and 393 ncRNAs were identified only by our study. Therefore, 621 ncRNA candidates were identified in *A. tumefaciens* C58 by two RNA-seq studies: 215 sRNAs, 386 asRNAs and 20 5′ UTR leaders (Table 3).

**Table 3:** Comparison of two *A. tumefaciens* RNA-seq studies.

	Number of ncRNAs					
Category	Wilms et al. [51]		Our study		Common^b^	Grand total
	Total	Unique^a^	Total	Unique^a^		
**sRNA**	149^c^	113	101	66^d^	36	**215**
**asRNA**	76	32	354	310	44	**386**
**5′ UTR leader**	3^c^	0	20	17	3	**20**
**Total**	228	145	475	393	83	**621**

a Unique ncRNAs were identified by one study but not by the other study.

b Common ncRNAs were identified by both RNA-seq studies.

c Three sRNA identified by Wilms et al. [51] were found to be 5′ UTR leaders in our study.

d One sRNA identified by our study overlaps with two sRNAs identified by Wilms et al. [51].

Interestingly, Wilms et al. [51] identified more sRNAs (149) than our study (101), while we identified many more asRNAs (354) than Wilms et al. [51] (76). This might be due to the differences in RNA-seq technology and ncRNA search algorithm. We treated the RNA samples consecutively with two methods to deplete rRNAs using hybridization oligos (MICROB*Express* ^TM^ kit, Ambion, USA) and TEX, while Wilms et al only treated their samples with TEX (e.g., Figure 1A&B Figure 2B in Wilms et al. [51]). The dual treatment in our study could help to obtain a higher overall coverage. In addition, we developed an ncRNA search algorithm, which identified genomic regions that did not overlap with any annotated genes and had at least ten times higher expression levels than neighboring regions (see Experimental procedures for detail). On the one hand, this algorithm has the strength to quickly identify highly expressed asRNAs, and indeed we did identify 354 asRNAs (6.6% of the 5,355 protein-coding genes, Table 3). On the other hand, some intergenic sRNAs may not be identified by this algorithm if adjacent genes are highly expressed at the same time. For example, the sRNAs C3 and Ti2 from the Wilms et al. [51] were not reported as a sRNA by our study because the immediate downstream genes (*dnaA* and *Atu6155*) were also highly expressed. However, it is also possible that some of the sRNAs identified by Wilms et al. [51] might be part of 5′ UTRs of protein coding genes. As shown in Figure S4 in File S2, for instance, our data suggested that C3 could be part of the 369 nt 5′ UTR of *dnaA* (Figure S4A in File S2) and Ti2 could be part of the 207 nt 5′ UTR of *Atu6155* (Figure S4B in File S2). Thirty-two sRNAs identified by Wilms et al. [51] appeared to be part of the long 5′ UTRs in our TSS mapping analysis (marked by ^†^ in Table S2 in File S1). In fact, the 5′ ends of 11 of those 32 sRNAs (including C3) were also identified as TSSs of protein-coding genes by Wilms et al. [51] (Table S2 in File S1). Another explanation could be that the bacterial growth conditions used for each RNA-seq study were different. Validation of all identified ncRNAs is needed for future studies.

### Differentially expressed ncRNAs

We identified differentially expressed ncRNAs by using the Bioconductor DESeq package [61]. Briefly, the number of reads mapped to each gene was calculated using a simple formula (Read count 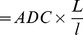, where L is the length of a gene and *l* is the length of a sequence read, 50), and normalized by effective cDNA library sizes. Differentially expressed ncRNAs were identified by comparing the full generalized linear model (GLM: ∼ treatment + TEX) against the null model (GLM: ∼ TEX).

We first identified differentially expressed ncRNAs (*P*<0.05) under induction conditions by AS (IND vs. AB) (Table S4-A, B in File S1). Fifteen ncRNAs were induced (Table S4-A in File S1), while 7 ncRNAs were suppressed (Table S4-B in File S1) by AS. Fourteen of the 15 AS induced ncRNAs have putative vir box sequences [62] in the upstream region (Table S4-A in File S1). It will be worthwhile to determine if some of these ncRNAs have regulatory roles during *Agrobacterium*-plant interactions.

We then identified differentially expressed ncRNAs during the stationary phase and the mid-log phase (YEP-S vs. YEP-L). Sixteen ncRNAs were accumulated during the stationary phase (Table S4-C in File S1) and 8 ncRNAs were suppressed (Table S4–D in File S1). Those ncRNAs accumulated during the stationary phase might be involved in stress-related responses [17].

### Validation of selected ncRNAs

**Figure 3:**
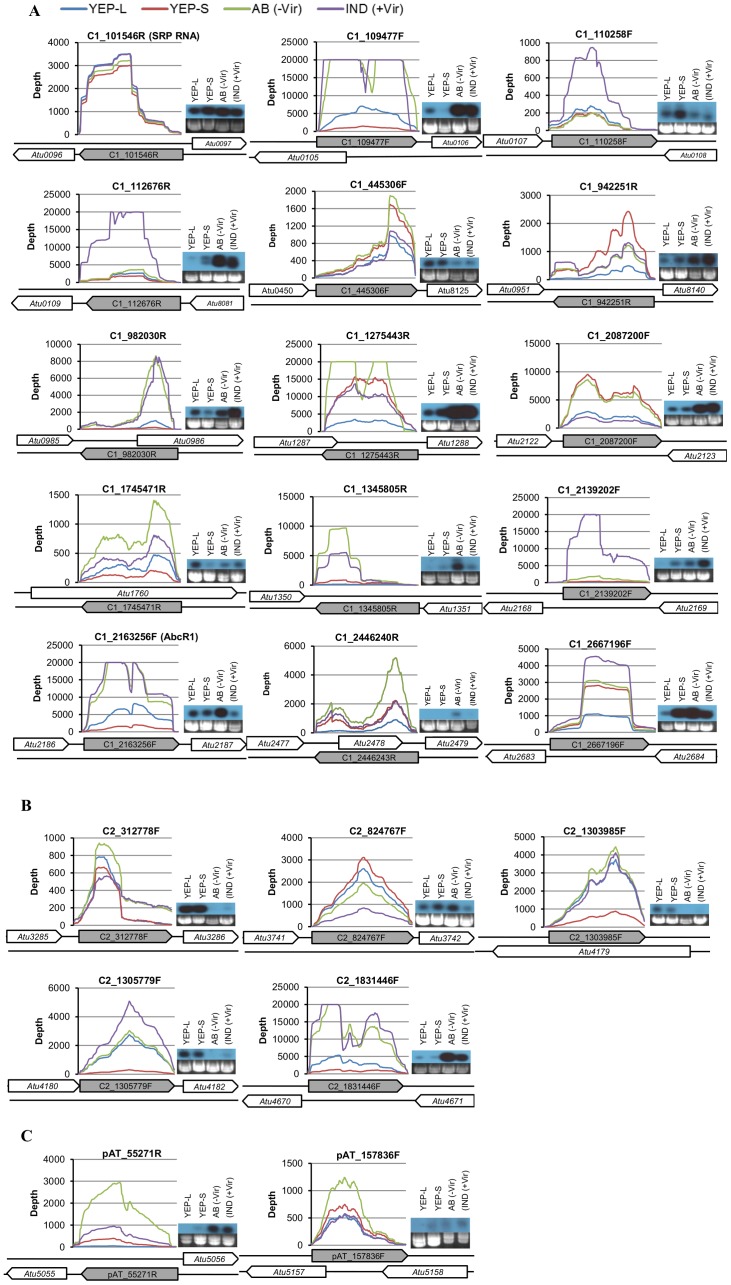
Validation of selected ncRNAs by Northern blot analysis. Depth of coverage profiles and Northern hybridization images of 22 *Agrobacterium* ncRNAs under four growth conditions: YEP medium until mid-log phase (YEP-L), YEP medium until late stationary phase (YEP-S), AB induction medium without AS (AB), AB induction medium with AS (IND). (**A**) Fifteen ncRNAs encoded on the circular chromosome (C1), (**B**) five ncRNAs encoded on the linear chromosome (C2), and (**C**) two ncRNAs encoded on the pAt plasmid (pAt).

To confirm the expression of the identified ncRNAs, we employed two independent techniques: Northern blot analysis and RACE. We validated a total of 36 ncRNAs. Northern blot analysis confirmed the expression of 24 of 28 ncRNAs (Table 4). Twenty-two representative ncRNAs are presented in Figure 3. RACE independently confirmed the expression of 16 of 18 ncRNAs (Table S5 in File S1) and we present the results for 9 ncRNAs found on the Ti plasmid (Figure S2 in File S2). Four ncRNAs were validated by both methods. Fourteen of the 36 validated ncRNAs, 9 by Northern blot analysis and 5 by RACE, were identified for the first time by this study.

**Table 4:** Validated ncRNAs with Northern blot analysis.

						RPKM											
		Position		Size (nt)		(−TEX)					(+TEX)						
	ncRNA tag	5′ end	3′ end	RNA seq	Northern blot	YEP-L	YEP-S	AB	IND		YEP-L	YEP-S			AB	IND	antisense to
**Circular chromosome**																	
1	C1_101545R† (SRP RNA)	101545	101446	100	∼100	10697	14477	13806	9254		6999	5692			3723	3674	Small SRP
2	C1_109477F†	109477	109594	118	∼120	1503	413	15354	24677		16287	3009			37950	34389	*Atu0105*
3	C1_109596F (thermosensor)	109596	109822	227	∼500	719	377	868	802		1346	1098			1443	1275	thermosensor
4	C1_110258F*	110258	110380	123	∼180	5420	5444	2955	3636		559	381			220	983	intergenic
5	C1_112676R†	112676	112535	142	∼140	541	402	819	8686		5028	3404			3819	23172	intergenic
					∼100												
6	C1_445306F	445306	445498	193	∼200	307	577	538	313		1377	2210			1519	776	intergenic
7	C1_942251R†	942251	942016	236	∼220	233	1171	830	1080		636	3179			1046	1109	intergenic
8	C1_982030R†	982030	981727	304	∼310	337	57	3986	5579		962	229			5155	4584	*Atu0986*
9	C1_1275443R†	1275443	1275297	147	∼150	2713	12965	25899	11016		8574	36917			30473	16440	*Atu1287*
10	C1_1345805R†	1345805	1345651	155	∼200	471	1602	3667	1634		454	1593			7684	4123	intergenic
					∼140												
11	C1_1745471R†	1745471	1745262	210	∼220	213	108	677	646		966	403			1676	851	*Atu1760*
12	C1_2087200F†	2087200	2087384	185	∼200	1661	8580	6413	1243		6545	20617			11237	2333	intergenic
13	C1_2139202F†	2139202	2139332	131	∼150	818	1387	1597	7564		644	902			2166	21501	intergenic
14	C1_2163256F†	2163256	2163370	115	∼110	5043	1273	28677	25589		17172	4036			27964	27289	suhB ( = AbcR1)
15	C1_2446240R†	2446240	2445919	322	∼330	476	1551	2583	1261		1032	3072			4164	1903	*Atu2478*
16	C1_2541934R* (TPP RS)	2541935	2541832	103	∼110	6422	13871	12566	5053		753	909			849	674	TPP riboswitch (*Atu2569*, *thiC*)
17	C1_2667196F* †	2667196	2667281	86	∼90	977	3379	1448	2276		2537	6206			4108	5534	Intergenic
**Linear chromosome**																	
1	C2_312778F (TPP RS)	312778	312932	155	∼150	337	566	510	281		582	481			636	463	TPP riboswitch (*Atu3286*)
2	C2_824767F	824767	824863	97	∼100	595	1432	642	259		5276	6043			2292	914	Intergenic
3	C2_1303985F	1303985	1304143	159	∼160	1597	430	1382	1800		7403	1648			4913	3853	*Atu4179*
4	C2_1305779F	1305779	1305881	103	∼120	1726	543	2406	3228		5564	580			3553	5343	Intergenic
5	C2_1831446F†	1831446	1831607	162	∼160	689	272	3458	3970		10541	2865			25085	25357	Intergenic
**At plasmid**																	
1	pAT_55271R* †	55271	55154	118	∼140	45	866	3597	877	105			717	3728		1042	Intergenic
2	pAT_157836F* †	157836	158083	248	∼270	288	656	622	259	966			1279	1243		506	*Atu5157* (*atsD*); *Atu5158*

* ncRNAs have been validated with 5′ and 3′ RACE.

† ncRNAs have been previously identified or detected by Wilms et al. [51].

F and R at the end of each ncRNA tag denote strand information: Forward and Reverse.

Among the 24 ncRNAs validated with Northern blot analysis, three were 5′ UTR elements, 14 were sRNAs and 7 were asRNAs. In most cases, the ncRNA sizes predicted by RNA sequencing were consistent with Northern blot analysis results with an exception of C1_10956F (thermosensor). This is because this ncRNA was not transcribed as an independent transcript (∼227 nt) but was transcribed as part of downstream gene in all four growth conditions (see below for detail). Two ncRNAs, C1_112676R and C1_1345805R had two bands (Table 4; Figure 3), suggesting that they might be transcribed from different promoters or they might be processed to become mature transcripts.

### Analysis of cis-antisense RNAs

Interestingly, while the expression level of all seven validated asRNAs varied considerably under different growth conditions (Table 4), the putative target mRNAs encoded on the complementary strand were not expressed at detectable levels or only expressed at a very low level (<10 RPKM). For example, the expression level of C1_109477F varied from 413 RPKM (YEP-S: −TEX) to 37950 RPKM (IND: +TEX) as shown in Table 4, but its putative target *Atu0105* (hypothetical protein; Ref 56 & 57) mRNA was not detectable in all eight cDNA libraries. Similarly, the expression level of C1_982034R varied from 57 RPKM (YEP-S: −TEX) to 5579 RPKM (AB: +TEX), but its putative target *Atu0986* (hypothetical protein; Ref 56 & 57) was not expressed at all.

**Figure 4:**
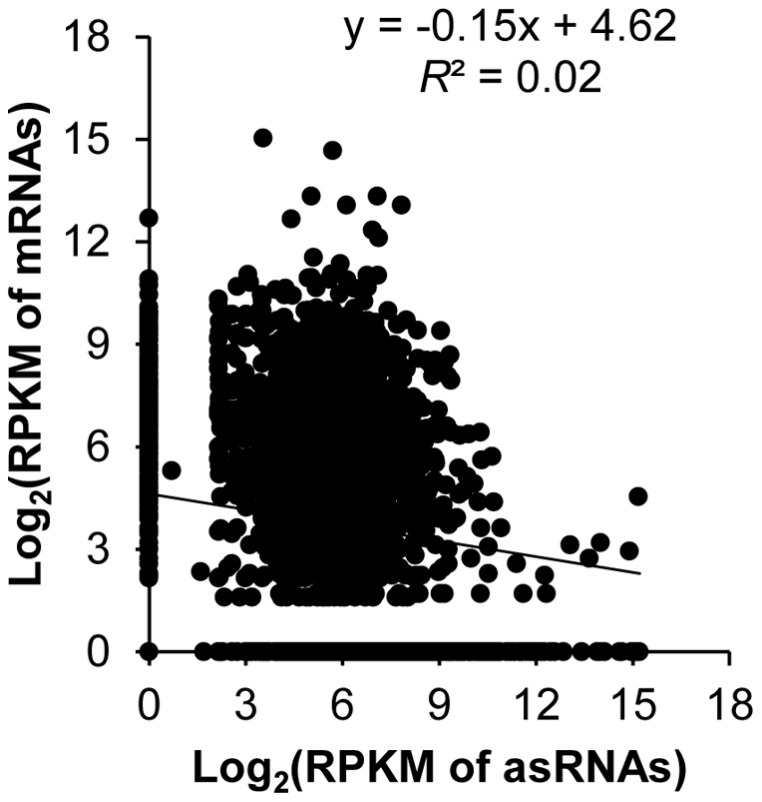
Expression correlation between *cis*-antisense RNAs and putative target genes. Log-transformed RPKM data for 354 *Agrobacterium* asRNAs were plotted against log-transformed RPKM data of genes encoded on the complementary strand. Pearson product-moment coefficient was given (*r* ^2^ = 0.02).

To investigate whether there was a general trend between the transcriptional levels of asRNAs and genes encoded on the complementary strands, we performed a Pearson product-moment correlation test. In a recent study, it has been shown that pervasive asRNAs play an important role for degradation of sense mRNAs by base-paring with them to form double stranded substrates of RNase III [63]. Furthermore, the presence of promoters on the opposite strands can affect expression of genes on the sense strand *via* transcription interference [64], [65]. The RPKM values of each asRNA and its putative target gene on the complementary strand were log-transformed before plotted. A Pearson product-moment test (SPSS 17; SPSS Inc., USA) showed that there was no evident correlation between the two (*r^2^* = 0.02; Figure 4). Clearly, there were many asRNAs with varying expression levels while their putative target genes on the opposite strands were not expressed at all. The lack of correlation might be attributed to the fact that some asRNAs may have positive effects while others have negative effects on target gene expression at the transcriptional level [52]. Alternatively, some of these so-called asRNAs may have their real targets encoded somewhere else in the genome; thus they might be *trans*-acting sRNAs. Because candidate asRNAs were named so solely due to the presence of annotated genes on the opposite strand, it is still possible that these ncRNAs may interact with other mRNAs that have sufficient sequence complementarity, especially when the genes encoded on the opposite strand are not expressed. A third possibility is that some candidate asRNAs might be protein-coding genes. We found that eight putative asRNAs contained a putative open reading frame (ORF; indicated by ^§^ in Table S3 in File S1). Because some of the annotated genes on the opposite strand of these candidate asRNAs were not detectable in all eight libraries, it is possible that the candidate asRNAs could be the protein-coding genes and the annotated genes on the opposite strand might represent pseudo genes.

As *A. tumefaciens* virulence is of great interest, it was intriguing to find that some asRNAs were encoded on the opposite strands of known virulence genes, such as *virC2*, *virB9*, *virB10*, *virD3*, *virD4*, *virE2* and *virE3*. To test if some of these asRNAs affect *A. tumefaciens* virulence, we chose two asRNAs: pAt_157836F is antisense to *atsD*, which might be important for bacterial attachment to plant cells [66], and pTi_191667R is antisense to *virB10* (*Atu6176*), an essential component of the Type IV secretion system that transports T-DNA into plant cells along with other effector proteins [67]. We generated a knock-out mutant strain, Δ*atsD*, in which the gene *atsD* and its antisense RNA pAt_157836F was deleted. We also generated overexpression strains of *A. tumefaciens* C58 that harbored replicating plasmid vectors carrying either the sense or antisense strands of the asRNA pAt_157836F driven by a constitutive promoter. Similarly, we made overexpression constructs for the sense and antisense sequences of pTi_191667R and introduced them into the wild type C58.

Tobacco leaf disk assay, *Arabidopsis* root segment assay and maize immature embryo transformation were performed as previously described [68]–[70]. Overexpression of pTi_191667R or its complementary sequence (anti-pTi_191667R) did not show detectable effects on *A. tumefaciens* virulence (Figure S5A in File S2). One explanation could be the limitation of the tobacco leaf disc assays for the quantitative virulence measurement. It has been suggested that bacterial small RNAs often have quantitative effects on the target gene expression [71]. Tobacco leaf disk assay may not be sensitive enough for measuring low level changes of *A. tumefaciens* virulence. Another explanation could be that the real target gene for pTi_191667R might not be its sense strand *virB10* gene, but rather a gene elsewhere in the genome.

Overexpression or knockout mutation of pAt_157836F also did not have significant effects on *A. tumefaciens* virulence measured by *Arabidopsis* root segment assay (Figure S5B in File S2). However, we observed marginally significant effects of the knockout mutation of *atsD* and pAt_157836F (Δ*atsD*) on maize immature embryo transformation frequency (Figure S5C in File S2; paired sample *t*-test, ***P*** = 0.017). Future work is needed to determine whether these ncRNAs have regulatory functions on other target genes that may affect bacterial phenotypes other than T-DNA delivery to plants.

### Two 5′ UTR elements function as a thermosensor and a thi-box riboswitch

**Figure 5:**
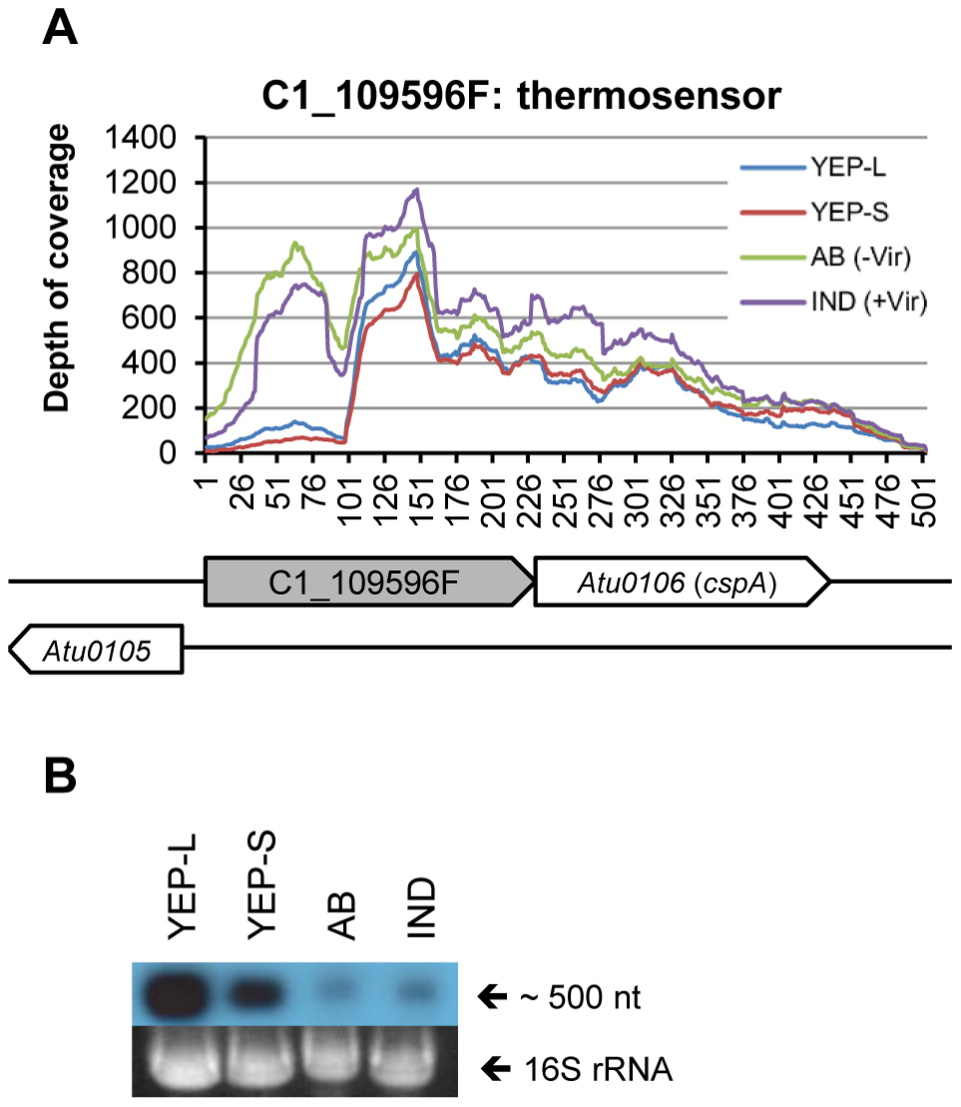
Expression profiling of a thermosensor, C1_109596F and a major cold shock protein, *cspA*. The depth of coverage data of the nucleotide positions of 109596–110198 on the Circular chromosome was plotted (+TEX). Northern blot analysis using a probe specific to the 5′ UTR showed that cspA is transcribed as an approximately 500 nt transcript, which was consistent with the RNA-seq results, 503 nt including 227 nt 5′ UTR (109596–109822), 210 nt *cspA* (*Atu0106*) coding region (109823–110032), and 66 nt 3′ UTR (110033–110098). YEP-L, YEP medium until mid-log phase, YEP-S, YEP medium until late stationary phase, AB, AB induction medium without AS, IND, AB induction medium with AS.

The two 5′ UTR elements (C1_109596F and C1_2541934R) were predicted to be *trans*-encoded sRNAs after initial screening, but C1_109596F was located immediate upstream of a cold shock protein (*Atu0106*: *cspA*) and C1_2541934R was found at the upstream of a thiamine biosynthesis operon (*thiCOGG*). A RNA family database search (Rfam: http://rfam.sanger.ac.uk/) suggested that they were homologous to a thermosensor (C1_109596F: http://rfam.sanger.ac.uk/family/cspA) and a thiamine riboswitch (C1_2541934R: http://rfam.sanger.ac.uk/family/TPP), respectively. A thermosensor is a 5′ UTR element of mRNAs and regulates translation of downstream coding sequence [72]. The secondary structure of a thermosensor changes depending on ambient temperature, and regulates the accessibility of the mRNA to ribosomes, thus affecting translation. One of the best studied thermosensors is located at the 5′ UTR of the global virulence regulator of *Listeria monocytogenes*, *prfA* [73]. Our Northern blot analysis suggested that C1_109596F is not expressed by itself (∼227 nt), but was transcribed as a 5′ UTR of *cspA* (Figure 5). Thus, the corresponding transcript of C1_109596F from Northern blot analysis was about 503 nt, including the 227 nt 5′ UTR, 210 nt coding sequence and 66 nt 3′ UTR (Figure 5). These results suggest that the thermosensor (C1_109596F) may post-transcriptionally regulate *cspA* expression like its homolog in *Escherichia coli* [74].

Riboswitches are located at the 5′ UTRs of many bacterial mRNAs and affect expression of downstream protein-coding regions upon binding of metabolites [75]. When there are sufficient metabolites, riboswitch-metabolite binding results in conformational changes in the RNA secondary structure leading to transcription termination by forming *rho*-independent terminator or to translation inhibition by masking the ribosomal binding site [76], [77]. Thiamine is an essential enzyme co-factor for carbon metabolism in all living organisms. Bacteria, fungi and plants can synthesize thiamine. The thi-box riboswitch, also known as TPP (thiamine pyrophosphate) riboswitch (RF00059), directly binds to TPP and regulates downstream gene expression by means of premature transcription termination (attenuation) or translation inhibition [78].

**Figure 6:**
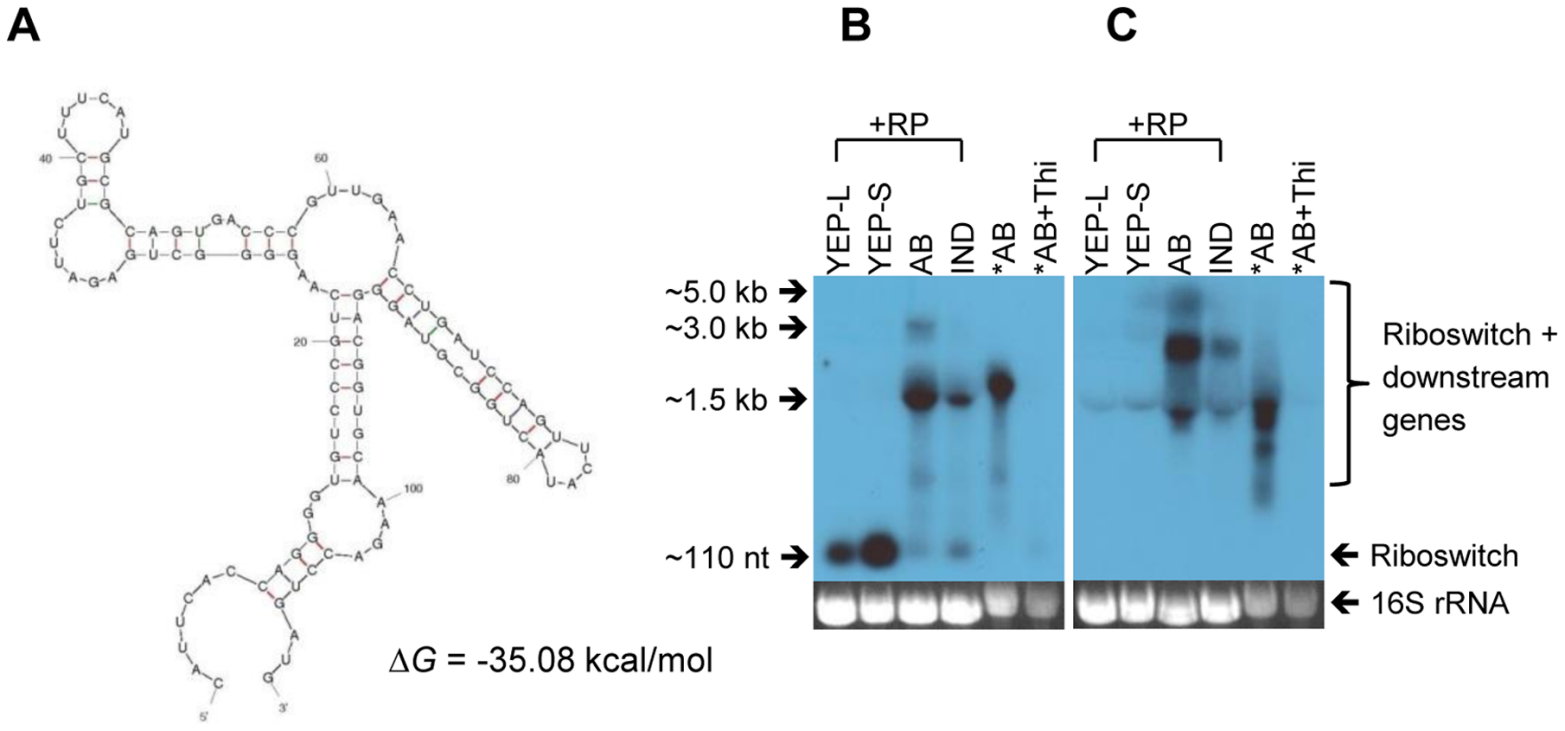
Transcriptional regulation of *thiCOGG* operon by a TPP riboswitch (C1_2541934R). A putative riboswitch at the 5′ UTR of thiamine biosynthesis operon, *thiCOGG*, transcriptionally regulates gene expression. **A**) Secondary structure predicted by mFold web server [94]: Δ*G*  = −35.08 kcal/mol. (**B**) Northern blot analysis with a probe specific to the riboswitch and (**C**) a probe specific to the downstream gene, *thiC* (**C**). Total RNA was isolated from *A. tumefaciens* strain C58 grown in YEP medium until mid-log phase (YEP-L), YEP medium until late stationary phase (YEP-S), AB induction medium without AS (AB), AB induction medium with AS (IND), and AB with 100 µg/mL of thiamine (AB+Thi). +RP, RNA samples were treated with RNAprotect Bacteria reagent (Qiagen, USA). *AB and *AB+Thi, RNA samples were not treated. Ethidiumbromide stained 16S rRNA bands were included as loading control.

According to the Rfam database, there were three TPP riboswitches in the *A. tumefaciens* C58 genome. Two TPP riboswitches were identified as candidate ncRNAs in our data set (C1_2541934R and C2_312778F) and the third one was also represented in our data set when we manually examined the predicted region in our files (Circular chromosome, 2700230–2700340, reverse strand). C1_2541934R was located in the 5′ UTR of an operon encoding proteins required for thiamine biosynthesis, *thiCOGG* (Figure 6A; Table 5). To determine whether this riboswitch is regulated by thiamine, as its homolog located at the 5′ UTR of *thiCOGE* in *Rhizobium etli* [78], we added thiamine to modified AB induction medium without AS (AB) to a concentration of 100 µg/mL. As can be seen in Figure 6, no *thiC* expression was observed in lanes YEP-L and YEP-S (Figure 6B&C) because YEP medium contains thiamine. Only the riboswitch (∼110 nt) was transcribed (Figure 6B), suggesting transcriptional regulation of the *thiCOGG* operon. However, *thiC* was expressed in the minimal medium (Figure 6B&C, lanes AB, IND and *AB) due to the absence of thiamine in the medium. Addition of thiamine clearly shut down transcription of downstream genes (Figure 6B& C, *AB+Thi), suggesting that this leader element works as a thi-box riboswitch. We also note that treating samples with the RNAprotect Bacteria reagent (Qiagen, USA) before RNA isolation can be important for stabilizing RNA molecules. Smaller bands observed in lane *AB (not treated) and AB (treated) in Figure 6C may represent degradation products of thiCOGG mRNA, demonstrating fast turnover of bacterial mRNAs [79].

**Table 5:** A thi-box riboswitch and thiamine biosynthesis gene operon.

					RPKM (−TEX)				RPKM (+TEX)			
Gene ID	5′ end	3′ end	Gene name	Product	YEP-L	YEP-S	AB	IND	YEP-L	YEP-S	AB	IND
*Atu2566*	2538732	2537959	*thiG*	thiazole synthase	0	0	28	117	0	0	47	114
*Atu2567*	2538934	2538737	*thiG*	sulfur carrier protein ThiS	0	0	14	92	0	0	36	112
*Atu2568*	2539905	2538931	*thiO*	thiamine biosynthesis oxidoreductase	0	0	28	107	4	0	65	179
*Atu2569*	2541730	2539907	*thiC*	thiamine biosynthesis protein ThiC	0	0	139	362	0	0	143	380
C1_2541934R	2541934	2541832		TPP riboswitch	6422	13871	12566	5053	753	909	849	674

Notably, the riboswitch transcript (∼110 nt) accumulated during the stationary phase (Figure 6B, YEP-S; Table S3A in File S1, C1_2541934R). The short transcript could be the truncated by-product caused by transcriptional attenuation [80]. But given that two S-adenosylmethionine (SAM) riboswitches, SreA and SreB, act as *trans*-acting sRNAs in *L. monocytogenes* [81], it would be worthwhile to examine if this thi-box riboswitch has additional targets *in trans*.

## Conclusion

We have generated a large date set consisting of over 840 million reads from 8 cDNA library representing four bacterial growth conditions and two treatments for enhancing RNA-seq quality (NCBI accession number, SRR747854). Depleting abundant rRNAs improved RNA-seq detection sensitivity, leading to the discovery of 384 novel ncRNAs. Our results show that numerous ncRNAs are transcribed from the opposite strands of many protein-coding genes as well as from the intergenic regions of the *A. tumefaciens* genome. Intriguingly, many asRNAs were discovered on the complementary strand of important virulence genes and operons, such as *virA*, *virB*, *virC*, *virD*, and *virE*. Furthermore, some candidate ncRNAs were differentially expressed when the cells are incubated with the *vir* gene inducer AS, suggesting that the identified ncRNAs may play a role in virulence regulation in *A. tumefaciens*. Whether these ncRNAs play crucial roles for physiological and cellular responses has yet to be elucidated, but their high abundance in the transcriptome suggests that they may have functional roles. Accumulating evidence strongly suggests that even tRNAs and protein-coding mRNAs can have regulatory functions [14], [82]–[85]. We speculate that future studies on ncRNAs functions during *Agrobacterium*-plant interactions will provide valuable tools to improve plant transformation efficiency as well as better understanding of fundamental plant-pathogen interactions.

## Experimental Procedures

### Media and bacterial growth conditions

*A. tumefaciens* C58 was grown at 28°C in YEP (10 g yeast extract, 10 g Bacto peptone, and 5 g NaCl per L, pH 7.0) or modified AB induction medium [1 g NH_4_Cl, 0.3 g MgSO_4_·7H_2_O, 0.15 g KCl, 0.01 g CaCl_2_, 2.5 mg FeSO_4_·7H_2_O, 2 mM phosphate buffer (pH 5.6), 50 mM 2-(4-morpholinoo)-ethane sulfonic acid (MES), 0.5% glucose per L, pH 5.6] with or without the vir gene inducer AS (100 µM) [86]. Cultures for strains carrying plasmid vectors were amended with appropriate antibiotics at the following concentrations: kanamycin, 50 µg/ml; spectinomycin, 100 µg/ml. Virulence gene induction was performed as described previously [86]. Briefly, *A. tumefaciens* cells were grown overnight in YEP medium containing appropriate antibiotics, if carrying plasmid vectors, and 0.5 mL culture was transferred to 50 mL AB-sucrose minimal medium containing appropriate antibiotics in a 250 mL flask. The culture was incubated at 28°C on a shaker-incubator (250 rpm) for about 16 hours. The bacterial densities were measured at OD_600_. The cultures were centrifuged at 4000×g for 10 min at room temperature, resuspended in two volumes of induction medium without AS (AB) and then divided equally (50 mL each) into two sterile 250 mL flasks. For virulence induction, AS was added to a final concentration of 100 µg/ml (IND) and incubated for 20 hours at 25°C (150 rpm).

### Whole transcriptome analysis

#### RNA sample preparation

Total RNA was isolated from *A. tumefaciens* cells using RNeasy Protect Bacteria mini kit (Qiagen, USA) according to the manufacturer's manual. Briefly, total RNA was extracted from *A. tumefaciens* strain C58 grown under four different growth conditions: YEP medium until mid-log phase (YEP-L: OD_600_  = 0.5), YEP medium until late stationary phase (YEP-S: OD_600_  = 1.3), modified AB induction medium without AS (AB: OD_600_  = 0.8), AB induction medium with AS (IND: OD_600_  = 0.8). One volume of *A. tumefaciens* culture (0.5∼2.5 mL) was mixed with two volumes of RNAprotect Bacteria reagent (Qiagen, USA), vortexed vigorously for 5 seconds, let sit for 5 min, and centrifuged for 10 min at 4000 x g at room temperature. Cell pellet was suspended in 200 µL of lysing buffer (10 mM Tris, 1 mM EDTA, pH 8.0) containing 15 mg/mL of lysozyme (Sigma) and incubated at 25°C for 10 min. Seven hundred µL of Buffer RLT containing 10 µL/mL of beta-mercaptoethanol was added, vortexed vigorously for 10 sec and centrifuged for 2 min at 15000×g. Only the supernatant was carefully transferred to a new tube and 500 µL of 100% ethanol was added and mixed by pipetting. Sample was applied to a spin column, centrifuged for 15 sec at 6000×g and the column was washed once with buffer RW1 and twice with buffer RPE (Qiagen, USA). To elute RNA from the column, 50 µL of RNase-free water was directly applied to the center of the membrane and centrifuged for 1 min at 21000×g. Contaminating DNA was removed by treating total RNA with DNase I (Invitrogen, USA) and checked by PCR.

Because the extreme abundance of rRNAs, which often account for 95–97% of total RNA in bacteria [26], is a major challenge for transcriptome analysis, we used two commercial kits to deplete rRNAs and tRNAs. A previous study demonstrated that rRNA content can be reduced by up to 19% by treating total RNA with MICROB*Express* ^TM^ kit (Ambion, USA), and then with 5′-phosphate-dependent exonuclease (TEX; Epicentre, USA) [26]. That is, mRNA content was increased from 5% to 25%, a 5-fold enrichment, which can improve mRNA detection sensitivity by up to 230% [26]. The MICROB*Express* ^TM^ kit uses hybridization oligos which specifically capture 16S and 23S rRNAs. TEX selectively digests processed RNA molecules with 5′ mono-phosphate, such as rRNAs, and is useful to enrich primary transcripts. About 10 µg of total RNA was treated MICROB*Express* ^TM^ kit (Ambion, USA) as recommended by the kit manual, and RNA integrity was checked by an Agilent 2100 BioAnalyzer. RNA samples were then further treated with TEX to remove remaining rRNAs and to enrich primary transcripts.

#### cDNA library preparation

cDNA libraries were prepared and analyzed at Illumina (San Diego, CA). The detailed protocol describes the steps for total RNA fragmentation, adapter ligation, reverse transcription, PCR amplification, purification, cluster generation and sequencing, and can be found in Illumina TruSeq Small RNA SamplePrep Guide (#15004197).

All purified total-RNA samples were started out with 100 ng in total volume of 16 µl. Each sample was treated with 2 µl of 5X fragmentation buffer (EPF#15016648, Illumina) and was incubated at 94°C for 4 minutes. Then the samples were cooled on ice.

The sample was mixed with a 7 µl master mix as following: 1 µl of RNAseOUT (40 U/µl) from Epicentre or RNAse Inhibitor (part#15003548, Illumina), 2 µl of T4 Polynucleotide Kinase (PNK) (part#M0201S, NEB), 2 µl of 10X PNK Buffer (part#M0201S, NEB), and 2 µl of 10 mM ATP (part#R109AT, Epicentre/Illumina). A 25 µl reaction mixture was incubated at 37°C for 1 hour on a pre-heated thermal cycler. The fragmented total-RNA samples (small RNA) were purified with procedure 1 of the RNA clean & concentrator-5 (part#R1015, Zymo Research), and were then eluted with 6 µl of RNase-free water.

Five microliter of purified fragmented RNAs were ligated with 1 µl RNA 3′ Adapter (RA3) (part# 15013207, Illumina); reactions were heated at 70°C for 2 minutes, then immediately cooled on ice. Next, a master mix of 4 µl was prepared as following before adding to the 6 µl reaction: 2 µl Ligation Buffer (HML) (part#15013206, Illumina), 1 µl RNase Inhibitor (part#15003548, Illumina), and 1 µl T4 RNA Ligase 2 Deletion Mutant (part# M0242S, NEB). The 10 µl reaction was incubated on the pre-heated thermal cycler at 28°C for 1 hour. With the reaction tube remaining on the thermal cycler, 1 µl Stop Solution (STP) (part#15016304, Illumina) was added to the reaction tube and mixed thoroughly by pipetting. Then the reaction mixture was incubated at 28°C for additional 15 minutes.

One microliter of RNA 5′ Adapter (RA5) (part#15013205, Illumina) was added into the 11 µl of the 3′ adapter ligation reaction mixture, and the sample was denatured at 70°C for 2 minutes and immediately cooled on ice. One microliter of 10 mM ATP (part#15007432, Illumina) and 1 µl of 10U T4 RNA ligase (part#1000587, Illumina) were added into the reaction to bring the final volume of 14 µl that was incubated at 28°C for another hour and then placed on ice.

For first strand cDNA synthesis, 6 µl of the 14 µl of 3′ and 5′ adapter ligated RNA samples was mixed with 1 µl RNA RT Primer (RTP) (part#15013981, Illumina), denatured at 70°C for 2 minutes, and then immediately cooled on ice. A 5.5 µl of master mix containing 2 µl 5X First Strand Buffer (part#18064-014, Invitrogen), 0.5 µl 12.5 mM dNTP mix (dilute from 25 mM dNTP mix, part #11318102, Illumina), 1 µl 100 mM DTT (part#18064-014, Invitrogen), 1 µl RNAse Inhibitor (part#15003548, Illumina), and 1 µl SuperScript II Reverse Transcriptase (part#18064-014, Invitrogen) was added and incubated at 50°C for 1 hour on a pre-heated thermal cycler.

A 50 µl PCR reaction was set up by adding 8.5 µl Ultra-Pure Water (part#1001913, Illumina), 25 µl PCR Mix (PML) (part#15022681, Illumina), 2 µl RNA PCR Primer (RP1) (part#15013198, Illumina), and 2 µl RNA PCR Primer Index (RPI1) (part#15013181, Illumina) into the 12.5 µl of the first strand cDNA reaction. PCR reaction was carried out in a thermal cycler with following profile: 98°C for 30 sec followed by 11 cycles of 98°C for 10 sec, 60°C for 30 sec, 72°C for 15 sec, and a final extension at 72°C for 10 min. The PCR product was held at 4°C until purification.

PCR products were purified using the Agencourt AMPure XP beads (part#A63881, Beckman Coulter Genomics) and verified with Agilent High Sensitivity DNA-1000 chip (part#5067-1504, Agilent), and the molar concentration for each sample was obtained. 10 pM of each cDNA library was used for clusters generation on cBot and sequencing was performed on Illumina sequencers (GAiix) with paired-end mode (2 x 50 bp). FASTQ files were generated using bcl2fastq script from CASAVA pipeline (Illumina).

#### Sequence alignment

For each of the 8 library data sets of Illumina RNA reads, the Bowtie 2 program [45] was used to map short reads in the data set onto the reference genome. Then the SAMtools program [46] was used to pile mapped reads with a mapping error rate of less than 1 in 10,000 along the reference genome. The pileup step allowed us to compute, for each position of each strand of the reference genome, its depth of coverage, which is the number of correctly mapped reads in sense orientation that cover the position.

### TSS Mapping for protein-coding genes

Transcriptional start sites (TSS) were identified as follows: 1) sequence reads were mapped to the *A. tumefaciens* reference genome (NC_003062.2, NC_003063.2, NC_003064.2, NC_003065.3) using the Bowtie 2 program [45], 2) depth of coverage (number of reads per nucleotide) for each nucleotide position on all four replicons were computed using SAMtools [46], 3) RPKM for all annotated protein-coding genes were computed, and 4) upstream regions of protein-coding genes that had expression levels greater than 50 RPKM were inspected to minimize erroneous annotations and a TSS was identified as a nucleotide position where the depth starts to steeply increase with a minimum value of 10.

### Identification of non-coding RNAs

Non-coding transcripts were identified as follows: 1) To find regions of much higher coverage depths by Illumina RNA reads, we selected the following regions sizes in bp: 800, 400, 200, 100, and 50. 2) For each of the above region sizes and each strand of the reference genome, a region of the size in the strand was reported to a file as having much higher coverage depths by Illumina RNA reads if the region has no overlap with any known protein coding region and the total sum of coverage depths of the region is at least 10 times higher than those of the non-overlapping regions of the same size right before and after the region, respectively. 3) Candidate ncRNAs were identified by manually examining the file of reported regions of much higher coverage depths and the file of all positions along with their coverage depths. 4) Transcriptional start and stop sites for each candidate ncRNA were determined based on the depth of coverage of upstream and downstream region of the search window with a minimum value of six.

### Identification of differentially expressed ncRNAs

Differentially expressed ncRNAs were identified using the Bioconductor DESeq package [61]. Firstly, the sequence reads were mapped against the reference genome using Bowtie 2 [45]. Secondly, the SAMtools program [46] was used to pile mapped reads and calculate depth of coverage for each nucleotide position. Thirdly, read counts per gene (annotated genes and identified ncRNAs) were computed for each sample (read count 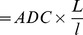; ADC, average depth of coverage  =  the number of reads that mapped to a nucleotide position on a given orientation, forward or reverse strand; *L*, length of a gene, *l*, length of sequence read  = 50). Lastly, read counts per gene were normalized by effective library sizes using DESeq package and differentially expressed ncRNAs were identified by comparing the full generalized model (GLM: ∼ treatment + TEX) against the null model (GLM: ∼ TEX) with a cut-off *P*-value of 0.05.

### RACE

3′ RACE (Rapid Amplification of cDNA Ends) were conducted as described previously [87]. Briefly, 10 µg of total RNA was ligated with 500 pmol of RNA oligonucleotide E1 using T4 RNA ligase (New England BioLabs Inc., USA) for 1 hr at 37°C and purified with phenol-chloroform extraction. First strand cDNA was synthesized using an oligonucleotide primer (5′-CATGCGGCCGCTAAGAAC-3′) specific to E1 and ThermoScript First strand synthesis kit (Invitrogen, USA), and PCR amplification was performed using E1-specific primer and a gene specific primer. As a control, duplicate samples were set up without reverse transcriptase (-RT). PCR products that were only obtained with +RT treatment were cloned into TA-cloning vector (5 PRIME Inc., USA) and their sequence was determined.

5′ RACE was also carried out similarly as described above, except that total RNA was treated with tobacco acid pyrophosphatase (TAP; Epicentre, USA) prior to 5′ RNA adapter ligation [88]. First strand cDNA was synthesized with either random hexamers or a gene-specific primer and PCR amplification was carried out with an adapter-specific primer and a gene-specific primer. PCR products obtained only after TAP treatment represent intact 5′ ends, thus they were cloned and sequenced to identify transcriptional start sites.

### Northern blot analysis

Northern blot analysis was carried out using NorthernMax®-Gly kit (Ambion, USA) according to the manufacturer's instruction. About 10 µg of total RNA was mixed with an equal volume of Glyoxal loading dye and incubated at 50°C for 30 min before loading. RNA Millennium^TM^ markers and RNA Century^TM^ markers (Ambion, USA) were loaded next to samples as size references. After electrophoresis, RNA was transferred to positively-charged membrane, UV cross linked, and hybridized overnight at 37–42°C with oligonucleotide probes end-labeled with ^32^P. Membranes were washed three times with washing buffers, and then exposed to X-ray films for 1–4 days at −80°C.

### Knock-out mutant generation

*Agrobacterium* knock-out mutant was generated as previously described [40]. Briefly, upstream and downstream flanking sequences of *atsD* were PCR amplified (upstream: atsD-UP-F1-SphI, atsD-UP-R1-SacII; downstream: atsD-DN-F1-SacII, atsD-DN-R1-EcoRI; Table S6 in File S1), cloned into cloning vector (5 PRIME, USA), and sequenced. Flanking sequences without point mutations were digested by restriction enzymes (upstream, SphI & SacII; downstream, SacII & EcoRI), separated on an agarose gel, and DNA bands were recovered from the gel and ligated to a suicide vector pK19mobsacB (ATCC 87098) [89] digested with SphI and EcoRI. After subcloning, *atsD* knockout plasmid was introduced into *A. tumefaciens* C58 by electroporation. Kanamycin resistant colonies were tested for sucrose sensitivity on LB medium (10 g tryptone, 5 g yeast extract, and 10 g NaCl per L) containing 10% sucrose. Two sucrose-sensitive colonies were picked and resuspended in 500 µL of LB broth, and 100 µL was spread on LB plate with 10% sucrose and incubated at 28°C for two days. Twenty to forty sucrose-resistant colonies were picked and tested for kanamycin susceptibility. Finally, sucrose-resistant and kanamycin-susceptible colonies were PCR screened using the upstream forward primer (atsD-UP-F1-SphI) and downstream reverse primer (atsD-DN-R1-EcoRI), and the fragment was cloned and sequenced to verify the deletion of *atsD*.

### Overexpression plasmid vector construction

The expression vector pTF505 (Figure S6 in File S2) was constructed as follows. Firstly, two replication origins (pBR322 and pVS1) were obtained from a binary vector pTF101.1 [90] by using restriction enzymes BssHII and SphI. Secondly, the selectable marker *aadA* (Sp^R^) was PCR-amplified from pL3 [91] (PaadAT-F2 and TpsbANT-R2; Table S6 in File S1) and digested with BssHII and KpnI. Thirdly, promotor-Multiple Cloning Sites (MCS)-terminator cassette was prepared as follows. A constitutive promoter P*rrnC* was predicted by a BLAST search using *Sinorhizobium meliloti* P*rrnC* sequence (AF252864) [92]. The 146 bp P*rrnC* promoter (NC_003305.1, from 1041328 to 1041473) was PCR amplified (AtuPrrnC-F1-KpnI and AtuPrrnC1-R-BglII; Table S6 in File S1), cloned into pPCV cloning vector (5 PRIME, USA), and sequenced at ISU DNA facility. Promoter activity was tested using mCherry [93] as a reporter gene, and the transcription start site was confirmed by 5′ RACE and sequencing. Oligonucleotides for multiple cloning sites (MCS) (MCS-BEF1 and MCS-BER1; Table S6 in File S1) were synthesized at ISU DNA facility, annealed, and treated with T4 PNK. Transcriptional terminator (T*psbA*) was PCR-amplified from pL3 using TpsbANT-F-EcoRI and TpsbANT-R-SphI (Table S6 in File S1), cloned and sequenced. P*rrnC* and TpsbANT were digested with restriction enzymes and ligated with MCS using the T4 DNA ligase (Promega, USA). Then P*rrnC*-MCS-TpsbANT cassette was PCR-amplified using AtuPrrnC-F1-KpnI and TpsbANT-R-SphI, cloned into pPCV cloning vector and sequenced. After KpnI and SphI digestion, P*rrnC*-MCS-TpsbANT cassette was recovered and ligated with the replication origin (pBR322 and pVS1) and *aadA* cassette using T4 DNA ligase (Promega, USA). To overexpress *atsD* and sense or antisense strands of pAt_157836F and pTi_191667R, PCR was carried out using the primers listed in Table S6 in File S1, and the fragments were cloned, sequenced, digested with restriction enzymes, and ligated to pTF505.

### Plant tumorigenicity assay

#### Tobacco leaf disk assay

Tobacco leaf disk assay was performed using *A. tumefaciens* C58 strains overexpressing an antisense RNA (pTi_191667R, antisense to *virB10*) or its complementary sequence (anti-pTi_191667R) with/without its native promoter (S/L) as previously described [69] with some modifications. Shortly, *Nicotiana tobacum* (Petit Havana) seeds were surface-sterilized using bleach and ethanol, and germinated on MS medium [69]. One to two weeks after germination, seedlings were transferred to magenta boxes containing germination medium and incubated up to 1 month, under 18-h light at 24–28°C.

*A. tumefaciens* strains were inoculated into 5 mL YEP medium and grown for 16 hours at 28°C (250 rpm). Two milliliters of overnight culture was transferred to 50 mL of YEP medium in a 250 mL flask and allowed to grow for 6 to 8 hours until OD_600_ reached 0.8. Cells were harvested by centrifugation at 4000×g, and resuspended to an OD_660_ of 0.5 in liquid MS medium. Inoculum was placed on ice until used.

For inoculation, tobacco leaf discs were prepared and inoculated as previously described [69]. After inoculation, eighteen leaf disks were carefully transferred onto MS medium and incubated for 48 hours. Leaf disks were then transferred onto a fresh MS medium containing 100 mg/L of cefotaxime. Tumor developments were monitored and the numbers of tumors on each leaf disk were recorded three weeks after inoculation.

#### *Arabidopsis* root segment transformation assay

*Arabidopsis* root segment transformation assay was conducted as described [68]. *A. tumefaciens* strains were inoculated into 5 mL YEP medium and grown overnight at 28°C. Two milliliter of overnight culture was transferred to 50 mL of YEP medium and allowed to grow for 6 to 8 hours until reached OD_600_ of 0.8 (10^9^ cells/mL). Cells were harvested by centrifugation at 4000×g, washed once in 0.9% NaCl, and then resuspended in 0.9% NaCl at a concentration of 10^8^ cells/mL. Inoculum was placed on ice until used.

For inoculation, *Arabidopsis* roots were cut into 0.3∼0.5 cm segments and transferred onto a Petri plate containing MS medium. Two to three drops of bacterial inoculum was placed onto root segments and left for 10 min. Bacterial suspension was removed by a pipette and the Petri plates were sealed and incubated for 48 hours in a growth chamber at 20°C. After cocultivation, root segments were rinsed with sterile water containing Timentin (100 mg/L). Root segments were transferred onto Petri plates containing fresh MS medium. Sixty root segments were used for each strain and the numbers of root segments with tumors were recorded three weeks after inoculation. Experiments were repeated 2–6 times for each strain.

#### Maize immature embryo transformation

Maize immature embryo transformation was conducted using disarmed *Agrobacterium* strain EHA105 and *atsD*/pAt_157836F knockout strain carrying a binary vector pTF101.1 [90] according to the published protocol [70]. For each infection experiment, 5 to 8 maize ears were used and 31 to 40 immature embryos were harvested from each ear; thus about 200 maize immature embryos were used for both strains. A total of three independent infection experiments were performed for this side-by-side comparison.

## Supporting Information

File S1 Table S1, Comparision of expression fold change of *vir* genes in a microarray study and two RNA-seq studies; Table S2, TSS-mapping; Table S3, List of identified candidate ncRNAs on all four replicons; Table S4, Differentially expressed candidate ncRNAs; Table S5, Selected ncRNAs for 5′ and 3′ RACE; Table S6, Oligonucleotides used in this study.
(PDF)

File S2 Figure S1, Effects of primary transcript enrichment by terminator 5′-phosphate-dependent exonuclease; Figure S2, 5′ and 3′ RACE for ncRNAs on Ti plasmid; Figure S3, Expression profiling of virD4* internal transcript with primary transcript enrichment (+TEX); Figure S4, Expression profiling of C3 and Ti2; Figure S5, Effects of two antisense RNAs (pTi_191667R and pAt_157836F) on *Agrobacterium* virulence; Figure S6, Map of the expression vector pTF505.
(PDF) Chi
