# Supplementary material for: A Genome-Wide Survey of Highly Expressed Non-Coding RNAs and Biological Validation of Selected Candidates in Agrobacterium tumefaciens
Source: PLoS One. 2013 Aug 8;8(8):e70720. doi: 10.1371/journal.pone.0070720 (PMC3738593; doi:10.1371/journal.pone.0070720)
Supplement: File S1 — Table S1, Comparision of expression fold change of vir genes in a microarray study and two RNA-seq studies; Table S2, TSS-mapping; Table S3, List of identified candidate ncRNAs on all four replicons; Table S4, Differentially expressed candidate ncRNAs; Table S5, Selected ncRNAs for 5′ and 3′ RACE; Table S6, Oligonucleotides used in this study. (PDF) [file pone.0070720.s001.pdf]

SUPPORTING INFORMATION FILE S1

A Genome-Wide Survey of Highly Expressed Non-Coding RNAs and Biological Validation of Selected Candidates in *Agrobacterium tumefaciens*

Keunsub Lee[1,2], Xiaoqiu Huang[3], Chichun Yang[1,2], Danny Lee[4], Vincent Ho[4], Kan Nobuta[4], Jian-Bing Fan[4], Kan Wang[1,2,*]

This file contains:

Table S1. Comparision of expression fold change of *vir* genes in a microarray study and two RNA-seq studies.

Table S2. TSS-mapping

Table S3. List of identified candidate ncRNAs on all four replicons

Table S4. Differentially expressed candidate ncRNAs

Table S5. Selected ncRNAs for 5' and 3' RACE

Table S6. Oligonucleotides used in this study

Table S1. Comparision of expression fold change of vir genes in a microarray study and two RNA-seq studies. The fold change of vir genes with and without AS induction was obtained and compared to the published results from a microarray study and a previous RNA seq study.

Locus tag	Gene name	Gene product	Fold change (+Vir vs. -Vir)		
			Klüsener et al. 2010* (microarray)	Wilms et al. 2012† (RNA seq)	This study (RNA seq)
Atu6166	*virA*	two-component VirA-like sensor kinase	16.26	5.29	22.64
Atu6167	*virB1*	type IV secretion system lytic transglycosylase VirB1	209.1	63.97	16603.07
Atu6168	*virB2*	type IV secretion system pilin subunit VirB2	209.2	29.78	6193.27
Atu6169	*virB3*	type IV secretion system protein VirB3	194.3	13.24	2356.47
Atu6170	*virB4*	type IV secretion system protein VirB4	217.3	48.31	1696.77
Atu6171	*virB5*	type IV secretion system protein VirB5	213.9	25	1582.04
Atu6172	*virB6*	type IV secretion system protein VirB6	147.5	39.71	1098.41
Atu6173	*virB7*	type IV secretion system lipoprotein VirB7	51.11	1	658.81
Atu6174	*virB8*	type IV secretion system protein VirB8	92.84	10	461.87
Atu6175	*virB9*	type IV secretion system protein VirB9	53.73	2.65	274.57
Atu6176	*virB10*	type IV secretion system protein VirB10	81.58	6.07	18.78
Atu6177	*virB11*	type IV secretion system protein VirB11	28.21	7.72	21.52
Atu6178	*virG*	two-component response regulator VirG	2.20	-	7.38
Atu6179	*virC2*	putative crown gall tumor protein VirC2	7.59	1.19	243.57
Atu6180	*virC1*	putative crown gall tumor protein VirC1	116	2.43	244.18
Atu6181	*virD1*	type IV secretion system T-DNA border endonuclease VirD1	169.2	15.44	1657.77
Atu6182	*virD2*	type IV secretion system T-DNA border endonuclease VirD2	134.7	48	1135.38
Atu6183	*virD3*	virA/G regulated protein	49.76	14.34	141.70
Atu6184	*virD4*	type IV secretion system protein VirD4	19.49	2.1	6.36
Atu6185	*virD5*	virA/G regulated protein	12.69	1.58	8.67
Atu6188	*virE0*	virA/G regulated protein	42.21	49.63	229.63
Atu6189	*virE1*	type IV secretion system chaperone	8.92	30.88	252.13
Atu6190	*virE2*	type IV secretion system single-stranded DNA binding protein	27.55	33.53	220.65
Atu6191	*virE3*	virA/G regulated protein	112.7	18.34	107.48
Atu6154	*virF*	exported virulence protein	42.2	6.62	22.88
Atu6150	*virH1*	P-450 monooxygenase	70.41	45.22	230.48
Atu6151	*virH2*	P-450 monooxygenase	89.28	67.28	308.59
Atu6156	*virK*	virA/G regulated gene	149.90	4.04	177.91
Atu6164	*tzs*	trans-zeatin secretion protein	68.34	116.91	614.98

*Microarray study: Klüsener *et al*. (2010). "Proteomic and transcriptomic characterization of a virulence-deficient phosphatidylcholine-negative *Agrobacterium tumefaciens* mutant." Molecular Genetics and Genomics 283(6): 575-589.

†RNA seq study: Wilms *et al*. (2012). "Deep sequencing uncovers numerous small RNAs on all four replicons of the plant pathogen *Agrobacterium tumefaciens*." RNA Biology 9(4): 22-21.

Table S2. TSS-mapping. Transcription start site (TSS) was mapped for 705 protein-coding genes. For each gene, start and stop positions for coding sequences were given along with the estimated 5' UTR lengths from this study and a previous study [1]. Gene product and conserved motif information is included in the last column.

Locus tag	Gene name	Strand	Coding region 5' end	Coding region 3' end	TSS	5' UTR length This study	5' UTR length Wilms et al. 2012 [1]	Gene product
**Circular chromosome**								
Atu0006	*secB*	-	4389	3907	4411	22	26	protein-export protein SECB
Atu0008	-	+	5215	5868	5108	107		hypothetical protein
Atu0012†	*gyrB*	+	8064	10499	7885	179	149	DNA gyrase subunit B
Atu0016	-	+	13605	14372	13603	2	0	hypothetical protein
Atu0022	*trxA*	-	19961	19641	20019	58		thioredoxin
Atu0029	*ahcY*	-	33282	31882	33333	51		S-adenosylhomocysteine hydrolase
Atu0031	-	-	34099	33698	34165	66	69	PTS system, IIA component
Atu0034	*chvI*	-	37250	36525	37316	66		two component response regulator
Atu0035	*pckA*	+	37589	39199	37549	40		phosphoenolpyruvate carboxykinase
Atu0048	-	-	50864	50151	50879	15		hypothetical protein
Atu0062	*frcR*	-	66286	65054	66388	102		transcriptional regulator, ROK family
Atu0063	*frcB*	+	66551	67573	66475	76		ABC transporter, substrate binding protein (sugar)
Atu0068	*nrdH*	+	70953	71174	70432	521	520	glutaredoxin protein; Cabalamin riboswitch (RF00174)
Atu0084	*rpsO*	-	87141	86872	87236	95		30S ribosomal protein S15
Atu0095	-	-	99469	99146	99517	48		hypothetical protein
Atu0101	*cycM*	+	104797	105375	104730	67		cytochrome c
Atu0106	*cspA*	+	109823	110032	109597	226	129	cold shock protein; thermoregulator (RF01766)
Atu0108	-	-	111835	110444	111908	73		putative metalloprotease M20 family
Atu0112	*rpmF*	+	117212	117397	117132	80		50S ribosomal protein L32
Atu0122	*dnaK*	-	128106	126205	128190	84	92	DNAK Protein
Atu0125	*msrA*	+	129114	129764	128913	201		peptide methionine sulfoxide reductase
Atu0126	-	+	129978	130970	129890	88		membrane lipoprotein
Atu0143	-	+	147053	148375	146993	60	70	MFS permease
Atu0151	*fabA*	-	160088	159573	160149	61		D-3-hydroxydecanoyl-(acyl carrier-protein) dehydratase
Atu0153	*irr*	+	160406	160825	160367	39	39	transcriptional regulator, Fur family
Atu0161†	*exbB*	+	166742	167764	166548	194		biopolymer transport protein
Atu0166*	-	-	171184	170930	171111	-73		hypothetical protein
Atu0186	*mepA*	-	189325	188270	189363	38		penicillin-insensitive murein endopeptidase
Atu0199	-	-	204114	203203	204171	57		ABC transporter, substrate binding protein (proline/glycine/betaine)
Atu0201	*aau3*	-	205950	205480	206096	146	146	hypothetical protein
Atu0224	*ctpA*	-	229818	229627	229879	61		components of type IV pilus, pilin subunit
Atu0228	-	-	231343	231038	231398	55		hypothetical protein
Atu0249	-	-	248527	247424	248557	30		ABC transporter, substrate binding protein (sugar)
Atu0250	-	-	249062	248655	249135	73		hypothetical protein
Atu0251	-	+	249269	250252	249248	21		hypothetical protein
Atu0260	-	-	259028	258027	259082	54		aldo-keto reductase
Atu0269	*xseA*	+	265041	266639	264956	85	88	exodeoxyribonuclease VII large subunit
Atu0277*	-	+	272422	272817	272543	-121		hypothetical protein
Atu0278*	-	-	273791	272904	273646	-145		hypothetical protein; N-terminus is 57 aa longer than homologs.
Atu0283	-	-	277818	277369	277839	21		hypothetical protein
Atu0288	-	-	283479	282202	283493	14		hypothetical protein
Atu0300	-	-	293563	292970	293614	51		methyltransferase
Atu0301	*dnaN*	-	294875	293757	294970	95		DNA polymerase III, beta chain

Locus tag	Gene name	Strand	Coding region		TSS	5' UTR length		Gene product
			5' end	3' end		This study	Wilms et al. 2012 [1]	
Atu0312	*cysK*	-	306061	305093	306093	32		cysteine synthase
Atu0322	*fadB*	+	315554	316315	315456	98		enoyl CoA hydratase
Atu0324†	*dnaA*	+	317547	319109	317178	369	370	chromosomal replication initiator protein DnaA
Atu0331	-	-	324938	324366	324966	28	156	sigma-54 modulation protein
Atu0338	*ihfB*	+	330144	330446	330112	32		integration host factor, beta subunit
Atu0344	-	+	335817	336698	335759	58		hypothetical protein
Atu8118	-	+	345749	346240	345688	61	48	putative universal stress protein
Atu0351	-	+	346383	346946	346347	36		hypothetical protein
Atu0354	*fur*	+	348239	348667	348190	49		ferric uptake regulator
Atu0356	*miaB*	+	349679	351046	349627	52		miaB protein
Atu0361	-	+	355747	356166	355647	100		transcriptional regulator
Atu0362	*metK*	+	356353	357615	356310	43		S-adenosylmethionine synthetase
Atu0365*	-	-	360735	359503	360728	-7		hypothetical protein; N-terminus is 13 aa longer than homologs.
Atu0371	*dapD*	-	365803	364949	365849	46		2,3,4,5-tetrahydropyridine-2-carboxylate N-succinyltransferase
Atu0375	*hspC*	+	370520	370945	370445	75		small heat shock protein
Atu0378	-	-	372713	372000	372713	0		hydrolase
Atu0382†	-	+	375023	375400	374933	90	90	hypothetical protein
Atu0396	-	+	389381	390877	389337	44		coenzyme A transferase
Atu0404	*pgi*	-	400057	398432	400080	23		glucose-6-phosphate isomerase
Atu0405	*fadD*	-	401900	400188	402101	201		long-chain-fatty-acid-CoA-ligase
Atu0406	*fbpA1*	+	402258	403307	402234	24		ABC transporter, nucleotide binding/ATPase protein
Atu0407	*fbpA*	+	403461	404477	403431	30		ABC transporter, substrate binding protein (iron)
Atu8120	-	-	410443	410201	410653	210		hypothetical protein
Atu0420	-	+	416278	417312	416237	41		ABC transporter, substrate binding protein (phosphate)
Atu0428	*argD*	+	424029	425228	423946	83		acetylornithine aminotransferase
Atu0431	*apaG*	-	427893	427501	427919	26		apaG protein
Atu8124	-	-	437917	437495	438003	86		hypothetical protein
Atu0443	-	-	439111	438416	439111	0		putative phage repressor
Atu0458	-	-	454615	454247	454630	15		hypothetical protein
Atu0466	-	-	458753	458394	458785	32	40	hypothetical protein
Atu0471	-	-	460756	460517	460775	19	19	hypothetical protein
Atu0472	-	+	461200	461757	461126	74		hypothetical protein
Atu0473*	-	-	462760	461846	462693	-67		hypothetical protein; N-terminus is 21 aa longer than homologs.
Atu0476	*aqpZ*	+	464352	465077	464328	24		aquaporin
Atu0484	-	-	473660	473010	473732	72	89	two component response regulator
Atu0488	-	-	476159	475893	476245	86		hypothetical protein
Atu0496	-	-	483701	483264	483754	53		hypothetical protein
Atu0513	-	-	503625	503248	503682	57	71	hypothetical protein
Atu0523	*fliF*	+	512099	513799	512072	27		flagellar M-ring protein
Atu0524†	*luxR*	+	514221	514901	513900	321	334	transcriptional regulator, LuxR family
Atu0528	*mscL*	+	521539	521967	521486	53		large conductance mechanosensitive channel protein
Atu0542	*fla*	-	532877	531936	532991	114		flagellin
Atu0545†	*flaA*	-	535408	534488	535539	131	134	flagella associated protein
Atu0555	*flgB*	-	541874	541482	541893	19		flagellar basal-body rod protein FlgB
Atu0560	*motA*	+	545748	546620	545724	24		flagellar motor protein
Atu0565	-	+	550470	550907	550391	79		hypothetical protein
Atu0573	-	+	558161	558832	558126	35	35	transcriptional regulator

Locus tag	Gene name	Strand	Coding region 5' end	Coding region 3' end	TSS	5' UTR length This study	5' UTR length Wilms et al. 2012 [1]	Gene product
Atu0590	*aglR*	-	574226	573201	574294	68		transcriptional regulator repressor
Atu0591	*aglE*	+	574504	575865	574444	60		ABC transporter, substrate binding protein (alpha-glucoside)
Atu8133	-	+	581170	581589	581151	19		hypothetical protein
Atu0600	*zwf*	-	587324	585849	587425	101		glucose-6-phosphate 1-dehydrogenase
Atu0607	*aceA*	-	594572	593283	594641	69		isocitrate lyase
Atu0609	*potF*	+	596390	597487	596363	27		ABC transporter, substrate binding protein (putrescine)
Atu0619*	-	+	607141	608841	607199	-58	-59	exopolyphosphatase; poorly conserved N-terminus.
Atu0622	-	+	611204	612187	611142	62		metallo-beta-lactamase superfamily protein
Atu0626	*adhP*	+	614537	615565	614497	40		alcohol dehydrogenase
Atu0634	*ispB*	-	623781	622765	623805	24		octaprenyl-diphosphate synthase
Atu0639	*glyQ*	+	626233	627165	626160	73		glycyl-tRNA synthetase, alpha subunit
Atu0640*	-	+	627263	627580	627273	-10		hypothetical protein
Atu0641	-	+	627774	628325	627751	23		hypothetical protein
Atu0652	-	-	645376	644810	645444	68	70	hypothetical protein
Atu8135†	-	+	649376	649669	649270	106		hypothetical protein
Atu0659	-	-	654237	653758	654278	41	31	NTP pyrophosphohydrolase, MutT family
Atu0661	-	-	656575	655238	656607	32		glycosyltransferase
Atu0665	*glcD*	+	660204	661637	660171	33	33	glycolate oxidase subunit
Atu0671	-	-	668335	667418	668461	126	126	hypothetical protein
Atu0675	-	-	670737	670510	670767	30		hypothetical protein
Atu0683	*groES*	-	678334	678038	678420	86		co-chaperonin GroES
Atu0687†	-	+	683918	684547	683833	85		hypothetical protein
Atu8086	-	+	692997	693236	692967	30		hypothetical protein
Atu0695	*kdtA*	+	693451	694773	693317	134		3-deoxy-D-manno-octulosonic acid transferase-like protein
Atu0710	*corA*	+	710699	711727	710591	108		magnesium/cobalt transport protein
Atu0713	*atpI*	+	715457	715822	715358	99		ATP synthase, subunit I
Atu0721	-	-	721838	720729	721979	141	139	hypothetical protein
Atu0726	-	+	726173	727417	726116	57		ring hydroxylating dioxygenase, alpha-subunit
Atu0728	-	+	728682	729143	728598	84	94	hypothetical protein
Atu0730	-	+	729366	729890	729215	151		hypothetical protein
Atu0738	*mcpG*	+	736601	738622	736510	91		chemotaxis methyl-accepting protein
Atu0740	-	+	739774	740226	739774	0		transcriptional regulator, AsnC family
Atu0752	-	+	749139	750335	749139	0	0	hypothetical protein
Atu0762	-	+	758574	758882	758553	21		hypothetical protein
Atu0764	-	-	759989	759552	760036	47	51	hypothetical protein
Atu0766	*ialB*	-	762203	761679	762252	49	25	invasion associated locus B
Atu0767	*coxB*	+	762636	763499	762542	94	96	cytochrome c oxidase subunit II
Atu0779	-	-	776147	775662	776172	25		peroxiredoxin
Atu0781	-	+	777258	777863	777197	61	60	hypothetical protein
Atu0788	-	+	786159	786731	786099	60		hypothetical protein
Atu0790	-	-	787829	787140	787858	29		hypothetical protein
Atu0794	*ccrM*	+	790755	791900	790724	31	31	cell cycle regulated site-specific DNA-methyltransferase protein
Atu0803	*ppdK*	+	801471	804197	801397	74		pyruvate,orthophosphate dikinase
Atu0804	-	-	806516	805152	806571	55		proton/glutamate transport protein
Atu0818	*cysH*	-	817254	816493	817275	21	21	phosphoadenosine phosphosulfate reductase
Atu0819	-	+	817480	817920	817445	35	3	hypothetical protein
Atu0820	-	+	818166	819191	818054	112		ABC transporter, substrate binding protein (sulfate)

Locus tag	Gene name	Strand	Coding region 5' end	Coding region 3' end	TSS	5' UTR length This study	5' UTR length Wilms et al. 2012 [1]	Gene product
Atu0824	-	-	822823	822482	822887	64	64	hypothetical protein; caulobacter sRNA CC2171 (RF01867)
Atu0827	-	-	826254	826018	826485	231		hypothetical protein
Atu8138	-	-	831188	830913	831216	28		hypothetical protein
Atu0835	*gst*	+	834645	835265	834557	88	96	glutathione S-transferase
Atu0837	-	-	836754	836239	836839	85		transcriptional regulator, MarR family
Atu0845	-	+	847373	848146	847340	33	24	hypothetical protein
Atu0848	-	+	849385	849768	849369	16	18	hypothetical protein
Atu0862	-	+	860240	860635	860041	199		hypothetical protein
Atu0870	*tam*	-	866782	866012	866806	24		trans-aconitate methyltransferase
Atu0876	*sodBI*	+	872849	873451	872802	47		superoxide dismutase
Atu0881	-	+	876145	876552	876072	73		cytochrome C-556
Atu0883	-	+	878016	878198	877640	376	376	hypothetical protein
Atu0887†	-	-	880420	879932	880573	153	153	acetyltransferase
Atu0898	-	+	889694	890095	889613	81		hypothetical protein
Atu0904	-	+	895877	896287	895692	185	257	hypothetical protein
Atu0916	*ros*	+	905301	905729	905031	270		COG4957 transcriptional regulator
Atu0920	-	-	908359	907934	908549	190		hypothetical protein
Atu0924†	-	+	913430	913624	913195	235		hypothetical protein
Atu0935	-	-	924748	924473	924766	18	18	hypothetical protein
Atu0939	*pemI*	+	928967	929233	928946	21		PemI protein
Atu0946	-	-	936337	935237	936363	26		dehydrogenase
Atu0962	-	-	951378	950974	951593	215		hypothetical protein
Atu0968	-	+	958125	958364	958051	74		hypothetical protein
Atu8142	-	+	958504	958758	958458	46		hypothetical protein
Atu0972	*lipA*	+	961121	961549	960941	180		lipA protein
Atu0977	*dop*	+	966101	967672	966054	47	57	serine protease DO-like protease
Atu0982	-	-	976886	974550	976934	48	50	two component sensor kinase
Atu0983	-	-	977433	977113	977472	39		hypothetical protein
Atu0995	-	+	991737	992480	991610	127		hypothetical protein
Atu0998	*bacA*	+	994197	995033	994164	33		undecaprenyl pyrophosphate phosphatase, possible bacitracin resistance protein
Atu0999	*mgtE*	-	996475	995102	996645	170		magnesium transport protein
Atu1002*	-	+	999240	1000670	999262	-22		Aminotransferase class-III; N-terminus is 20 aa longer than homologs
Atu1009	-	-	1005095	1004757	1005270	175		hypothetical protein
Atu8144	-	+	1005698	1005961	1005573	125	125	hypothetical protein
Atu8146	-	-	1010849	1010508	1011132	283		hypothetical protein
Atu1018	-	+	1011390	1011698	1011375	15		hypothetical protein
Atu1020	-	+	1012325	1013434	1012212	113		outer membrane protein
Atu1021	-	+	1013942	1015039	1013869	73	73	outer membrane protein
Atu1024	*dapA*	+	1017629	1018513	1017576	53	55	dihydrodipicolinate synthase
Atu1025	*smpB*	+	1018677	1019159	1018642	35		SSRA-binding protein
Atu1028	-	-	1021746	1021177	1021836	90	102	hypothetical protein
Atu1029†	*rnpO*	+	1022139	1022528	1021931	208		DNA-directed RNA polymerase omega subunit
Atu1031	-	+	1025053	1025238	1025014	39	45	hypothetical protein
Atu1056	-	-	1048445	1048008	1048507	62		COG5451 hypothetical protein
Atu1066*	-	+	1058145	1058780	1058160	-15	-15	hypothetical protein; N-terminus is 5 aa longer than homologs
Atu1068	-	-	1061748	1060840	1061773	25	25	hypothetical protein

Locus tag	Gene name	Strand	Coding region 5' end	Coding region 3' end	TSS	5' UTR length This study	5' UTR length Wilms et al. 2012 [1]	Gene product
Atu1088	*rplI*	-	1080619	1080044	1080720	101		50S ribosomal protein L9
Atu1091	*rpsF*	-	1082478	1082017	1082703	225		30S ribosomal protein S6
Atu1096	*acpP*	+	1086146	1086382	1085929	217		acyl carrier protein
Atu1105	-	-	1094625	1093678	1094757	132		hypothetical protein
Atu1108	-	-	1099556	1098387	1099654	98		hypothetical protein
Atu1110	*pepA*	+	1099854	1101347	1099811	43		cytosol aminopeptidase
Atu1112	-	+	1101977	1103005	1101916	61		hypothetical protein
Atu1131	*ropB*	+	1120603	1121259	1120547	56		outer membrane protein
Atu1142	-	+	1130984	1132141	1130953	31		permease
Atu1160	-	-	1155583	1155125	1155643	60	81	hypothetical protein
Atu1162*	-	+	1156778	1157305	1156835	-57		hypothetical protein; N-terminus is 30 aa longer than homologs.
Atu1164	-	+	1158423	1159754	1158248	175		hypothetical protein
Atu1165	*glyA*	+	1160109	1161398	1160085	24		serine hydroxymethyltransferase
Atu1174	*rrpP*	+	1167264	1169402	1167138	126	134	H+ translocating pyrophosphate synthase
Atu1175	-	-	1170010	1169477	1170153	143		hypothetical protein
Atu1176	-	+	1170283	1170819	1170226	57		COG5452 hypothetical protein
Atu1180	*ihfA*	+	1173774	1174112	1173737	37	37	integration host factor alpha subunit
Atu1181	-	+	1174387	1174920	1174257	130		hypothetical protein
Atu1210	*cpdB*	-	1205715	1203727	1205753	38		putative 2,3-cyclic nucleotide 2-phosphodiesterase/3-nucleotidase
Atu8149	-	-	1206234	1205899	1206263	29		hypothetical protein
Atu1217	*cysK*	-	1211990	1210953	1212050	60		cysteine synthase
Atu1221	-	+	1214843	1215286	1214823	20		hypothetical protein
Atu1222*	-	+	1215424	1216155	1215730	-306		hypothetical protein; N-terminus is 109 aa longer than homologs.
Atu1225	-	+	1217373	1217645	1217340	33		hypothetical protein
Atu1232	-	+	1221425	1221961	1221385	40	27	hypothetical protein
Atu1244	*argC*	-	1234878	1233946	1234971	93		N-acetyl-gamma-glutamyl-phosphate reductase
Atu1247	*rplM*	-	1236968	1236504	1237087	119		50S ribosomal protein L13
Atu1250	-	+	1238961	1239395	1238833	128		hypothetical protein
Atu1258	*clpP*	+	1246266	1246898	1246154	112	63	ATP-dependent Clp protease, proteolytic subunit
Atu1261	*lon*	+	1248881	1251298	1248621	260		ATP-dependent protease LA
Atu1262	*hupA*	+	1251588	1251863	1251522	66		histone-like protein
Atu1268	*nuoA*	+	1256220	1256585	1256137	83	78	NADH ubiquinone oxidoreductase chain A
Atu1292	*dnaE*	+	1279704	1283207	1279441	263	213	DNA polymerase III, alpha chain
Atu8152	-	-	1286275	1285967	1286337	62		hypothetical protein
Atu1296	*divK*	+	1286407	1286778	1286327	80		two component response regulator
Atu1299	*rpmG*	-	1289243	1289076	1289298	55		50S ribosomal protein L33
Atu1312	-	+	1304715	1305011	1304700	15		hypothetical protein
Atu1322	-	+	1311479	1311988	1311465	14		acetyltransferase
Atu1324	*gatB*	+	1312454	1313959	1312430	24		glutamyl-tRNA amidotransferase subunit B
Atu1327	-	+	1315245	1315643	1315192	53	53	NADH-ubiquinone oxidoreductase
Atu1332	*aroQ*	-	1319157	1318720	1319178	21		3-dehydroquinate dehydratase
Atu1338	*fabG*	+	1322983	1323687	1322938	45		COG1028IQR 3-oxoacyl-(acyl carrier protein) reductase
Atu8016	-	-	1327134	1326994	1327159	25		hypothetical protein
Atu1341	*mrcA*	+	1328956	1331403	1328765	191		penicillin-binding protein 1a
Atu1342	*prfB*	+	1331605	1332633	1331475	130		peptide chain release factor 2
Atu1346	-	+	1334804	1335721	1334772	32		phenazine antibiotic biosynthesis related protein
Atu1347	-	+	1335943	1337892	1335827	116		Tetracycline resistance protein, tetM/tetO subfamily

Locus tag	Gene name	Strand	Coding region 5' end	Coding region 3' end	TSS	5' UTR length This study	5' UTR length Wilms et al. 2012 [1]	Gene product
Atu1348	-	-	1344332	1337985	1344670	338		hypothetical protein
Atu1355	-	-	1349065	1348505	1349096	31		hypothetical protein
Atu1358	-	-	1352387	1351878	1352453	66		hypothetical protein
Atu1359	*dksA*	+	1352657	1353076	1352568	89	28	dnaK deletion suppressor protein
Atu1372	-	-	1366852	1366382	1366918	66		hypothetical protein
Atu1373	-	+	1366969	1367811	1366642	327		hypothetical protein
Atu1381	*omp1*	+	1374514	1376838	1374375	139		group 1 outer membrane protein precursor
Atu1392	*cisY*	-	1386476	1385187	1386535	59		citrate synthase
Atu1395	*lexA*	-	1390892	1390170	1390931	39	47	LexA repressor
Atu1418	*metF*	-	1415290	1414370	1415308	18		methylenetetrahydrofolate reductase
Atu1428	-	+	1423178	1423495	1423148	30		hypothetical protein
Atu1429	*pdhA*	+	1423772	1424692	1423617	155		pyruvate dehydrogenase alpha subunit
Atu1450	*hfq*	+	1446124	1446366	1445978	146	148	putative RNA-binding protein Hfq
Atu1460	-	+	1456344	1456847	1456327	17		hypothetical protein
Atu1464	*gcvT*	-	1462163	1461090	1462469	306	239	glycine cleavage system T protein, aminomethyltransferase; Glycine riboswitch (RF00504)
Atu1467	-	+	1463055	1463435	1463013	42		hypothetical protein
Atu1469	-	-	1465039	1464719	1465070	31		hypothetical protein
Atu8157	-	+	1471602	1472054	1471577	25	25	hypothetical protein
Atu1500	*rlpA*	-	1490983	1489940	1491150	167	145	rare lipoprotein A
Atu1501	-	+	1491357	1491701	1491338	19		hypothetical protein
Atu1505	*dac*	+	1494021	1495520	1493984	37		penicillin-binding protein
Atu1507	*gyrA*	-	1498681	1495961	1498813	132		DNA gyrase subunit A
Atu1512	*ssb*	-	1502084	1501563	1502240	156		single-strand DNA binding protein
Atu1513	*uvrA*	+	1502391	1505312	1502272	119	123	ABC excinuclease subunit A
Atu1521	*znuA*	+	1510841	1511830	1510820	21		ABC transporter, substrate binding protein (zinc)
Atu1526	*gntZ*	-	1519041	1517611	1519068	27		6-phosphogluconate dehydrogenase, decarboxylating
Atu1537	*fixN*	-	1528520	1526898	1528599	79		cytochrome-c oxidase, FixN chain
Atu1546	-	-	1536566	1536180	1536620	54	20	transcriptional regulator, MerR family
Atu8158	-	+	1542139	1542306	1542102	37		hypothetical protein
Atu1554	-	+	1543000	1543188	1542976	24		hypothetical protein
Atu1563	*yajC*	-	1551372	1551025	1551410	38	55	preprotein tranlocase protein
Atu1567	*fdhA*	+	1553545	1554741	1553355	190		glutathione-independent formaldehyde dehydrogenase
Atu1571	*cysE*	-	1558205	1557381	1558250	45		serine acetyltransferase
Atu1573	-	+	1559353	1559598	1559300	53		hypothetical protein
Atu1577	-	+	1562468	1563406	1562290	178		ABC transporter, substrate binding protein (amino acid)
Atu1584	-	+	1567482	1567700	1567300	182	31	hypothetical protein
Atu1589	-	-	1572923	1571703	1573159	236		aminotransferase, class I
Atu1592	-	-	1575924	1575229	1576050	126	126	hypothetical protein
Atu1600	*acpP*	-	1581860	1581576	1581892	32		acyl carrier protein
Atu1602	*fnrN*	+	1583538	1584263	1583437	101		transcriptional activator, Crp family
Atu1608	-	+	1589763	1590185	1589709	54	54	hypothetical protein
Atu1610	*dhs*	-	1593372	1591996	1593420	48		2-dehydro-3-deoxyphosphoheptonate aldolase
Atu1613	*rpiA*	-	1596419	1595739	1596443	24		ribose 5-phosphate isomerase
Atu1615	-	-	1599456	1597528	1599580	124		hypothetical protein
Atu1616	*fumC*	+	1599895	1601286	1599839	56		fumarate hydratase
Atu1620	*tpiA*	-	1605674	1604904	1605758	84		triosephosphate isomerase
Atu1632	-	-	1620247	1617734	1620299	52		dimethylglycine dehydrogenase
Atu1634	-	+	1623203	1623457	1623186	17		hypothetical protein
Atu1637	-	+	1624601	1624933	1624577	24		hypothetical protein

Locus tag	Gene name	Strand	Coding region 5' end	Coding region 3' end	TSS	5' UTR length This study	5' UTR length Wilms et al. 2012 [1]	Gene product
Atu1640	-	-	1626651	1626403	1626676	25		hypothetical protein
Atu1643	-	+	1627179	1627616	1627113	66		hypothetical protein; mini-ykkC motif (RF01068)
Atu1647	-	-	1631682	1630522	1631812	130		ABC transporter, membrane spanning protein
Atu1657	*nadE*	-	1643323	1641644	1643512	189		NAD (+) synthetase
Atu1661	*sthA*	+	1646893	1648296	1646788	105		soluble pyridine nucleotide transhydrogenase
Atu1662	-	-	1649143	1648496	1649282	139	144	hypothetical protein
Atu1663	-	+	1649579	1650568	1649434	145		amidohydrolase
Atu1667*	-	+	1653073	1653306	1653079	-6	-7	hypothetical protein; N-terminus is 29 aa longer than homologs.
Atu8161	-	+	1653758	1653901	1653691	67		hypothetical protein
Atu1670	*adhC*	+	1655875	1657002	1655793	82		alcohol dehydrogenase class III
Atu8162	-	+	1658132	1658395	1658015	117		hypothetical protein
Atu1683	-	+	1669058	1669282	1668850	208	16	hypothetical protein
Atu1694	-	-	1680027	1679659	1680053	26		hypothetical protein
Atu1695	*moaA*	+	1680160	1681209	1680080	80	54	molybdenum cofactor biosynthesis protein A
Atu1700	-	-	1686421	1684808	1686521	100		lipoprotein
Atu1710	-	-	1697593	1694567	1697752	159		hypothetical protein
Atu1713	-	+	1700924	1701256	1700810	114		hypothetical protein
Atu1716	-	-	1703496	1703179	1703549	53		hypothetical protein
Atu1717	*fadL*	-	1705033	1703756	1705051	18		long-chain fatty acid transport protein
Atu1719	*valS*	-	1708330	1705487	1708428	98		valyl-tRNA synthetase
Atu1720	-	-	1709550	1708549	1709746	196		hypothetical protein
Atu1724*	-	+	1711857	1712057	1711877	-20	-20	hypothetical protein
Atu1733†	*nrdE*	+	1717324	1721142	1716989	335		ribonucleoside-diphosphate reductase 2 alpha chain
Atu1736*	*mntH*	-	1723365	1721995	1723355	-10		manganese transport protein; N-terminus is 15 aa longer than homologs.
Atu1739	-	+	1725944	1727182	1725885	59		permease
Atu1748	*nifU*	-	1736022	1735576	1736131	109		NIFU-like protein
Atu1749	*folE*	+	1736303	1736929	1736136	167		GTP cyclohydrolase I
Atu1751	-	+	1737604	1738542	1737553	51		hypothetical protein
Atu1752	-	-	1739057	1738623	1739114	57		hypothetical protein
Atu8163	-	+	1741044	1741661	1741028	16		hypothetical protein
Atu1757	-	+	1742384	1742992	1742368	16	18	hypothetical protein
Atu8019	-	-	1747199	1747038	1747250	51		hypothetical protein
Atu1764*	*metF*	+	1748259	1749179	1748267	-8		methylenetetrahydrofolate reductase
Atu1769	*glnB*	+	1753834	1754172	1753793	41	60	nitrogen regulatory protein PII
Atu1773	-	-	1757847	1757191	1757969	122		hypothetical protein
Atu1779	-	-	1766306	1765995	1766340	34		hypothetical protein
Atu1783	-	-	1769444	1769199	1769465	21		hypothetical protein
Atu8021	-	-	1785368	1785261	1785394	26		hypothetical protein
Atu1803	*cspA*	+	1788458	1789036	1788412	46		cold shock protein
Atu1805	-	-	1789827	1789579	1789954	127		hypothetical protein
Atu1810	-	+	1792062	1792367	1791975	87		hypothetical protein
Atu8022	-	-	1794795	1794640	1794860	65		hypothetical protein
Atu1824	*sufB*	-	1804578	1803109	1804747	169		ABC transporter subunit
Atu1825	*nifS*	-	1805946	1804777	1806050	104		cysteine desulfurase
Atu1830	*bcp*	+	1813147	1813614	1813067	80		bacterioferritin comigratory protein
Atu1832	-	+	1814546	1815844	1814531	15		membrane protein associated metalloendopeptidase
Atu1833	-	+	1816064	1817575	1816002	62		COG0513LKJ ATP-dependent RNA helicase
Atu1849	-	+	1829261	1829851	1829241	20	23	hypothetical protein

Locus tag	Gene name	Strand	Coding region 5' end	Coding region 3' end	TSS	5' UTR length This study	5' UTR length Wilms et al. 2012 [1]	Gene product
Atu1853	-	+	1833841	1834089	1833804	37		hypothetical protein
Atu1854	*grlA*	+	1834234	1834569	1834190	44		glutaredoxin-related protein
Atu1860	*rpsD*	-	1840307	1839690	1840511	204		30S ribosomal protein S4
Atu1862	-	-	1843326	1841782	1843346	20		hypothetical protein
Atu1864	-	-	1844991	1844353	1845032	41	22	putative homoserine/homoserine lactone efflux protein
Atu1866*	-	-	1845744	1845550	1845671	-73		hypothetical protein; N-terminus is 33 aa longer than homologs.
Atu1869	-	-	1848056	1847538	1848098	42		hypothetical protein
Atu1870	*icdA*	+	1848284	1849498	1848242	42		isocitrate dehydrogenase
Atu1874	*recA*	-	1855017	1853926	1855184	167	48	RecA protein
Atu1877	-	-	1859681	1857441	1859768	87		OmpA family protein
Atu1879	-	+	1860027	1860800	1859934	93		ABC transporter, substrate binding protein (amino acid)
Atu1880	-	+	1860913	1861716	1860872	41	41	ABC transporter, membrane spanning protein (amino acid)
Atu1882	*usg*	+	1862712	1862978	1862667	45		COG5425 usg protein
Atu1885	-	-	1865769	1865281	1865792	23	23	transcriptional regulator, AsnC family
Atu1895	*ribB*	+	1872469	1873110	1872201	268	267	3, 4-dihydroxy-2-butanone 4-phosphate synthase
Atu1913	-	+	1890366	1890680	1890339	27		hypothetical protein
Atu1947	*rpsJ*	-	1915718	1915410	1916070	352	182	30S ribosomal protein S10
Atu1951	*rpsL*	-	1920324	1919953	1920582	258		30S ribosomal protein S12
Atu1952	-	+	1920852	1921145	1920786	66		hypothetical protein
Atu1956	*rpoB*	-	1931334	1927198	1931474	140		DNA-directed RNA polymerase beta chain
Atu1962	*secE*	-	1934960	1934760	1935251	291		secretion protein
Atu8023	-	+	1938858	1938971	1938828	30		hypothetical protein
Atu1971	-	-	1940502	1940053	1940512	10		hypothetical protein
Atu2001*	*uvrB*	+	1962842	1965808	1962858	-16	0	excinuclease ABC subunit B
Atu2006	*dnaJ*	-	1969321	1968719	1969382	61		molecular chaperone, DnaJ family
Atu8168	-	+	1969706	1970059	1969679	27		hypothetical protein
Atu2015	-	-	1978604	1977360	1978747	143		aminotransferase
Atu2025	-	+	1986648	1987343	1986310	338		transcriptional regulator
Atu2045	*hflK*	-	2005187	2004069	2005244	57		HFLK protein
Atu8170	-	-	2007050	2006742	2007098	48		hypothetical protein
Atu8171	-	+	2007792	2008298	2007602	190	132	hypothetical protein
Atu2055	-	-	2019007	2017856	2019115	108		HlyD family secretion protein
Atu2067	-	-	2029464	2029228	2029521	57		hypothetical protein
Atu2073	*sco1*	-	2034701	2034093	2034744	43		membrane protein
Atu2075	*cheW*	+	2035226	2035693	2035122	104		chemotaxis protein
Atu2080	-	+	2039307	2039840	2039158	149	153	acetyltransferase
Atu2085	*lpxC*	-	2046848	2045892	2046951	103	102	UDP-3-0-(3-hydroxymyristoyl) N-acetylglucosamine deacetylase
Atu2089	*ddlB*	-	2052137	2051211	2052323	186		D-alanylalanine synthetase
Atu2099	*murE*	-	2065178	2063709	2065219	41		UDP-N-acetylmuramoylalanyl-D-glutamate-2,6-diaminopimelate ligase
Atu2103	-	-	2069026	2068586	2069193	167	171	hypothetical protein
Atu2117	-	+	2081194	2082270	2081113	81		lytic murein transglycosylase
Atu2124	-	-	2089316	2088303	2089347	31	31	transcriptional regulator, ArsR family
Atu2133	-	+	2098161	2099543	2098127	34		hypothetical protein
Atu2136	*parA*	-	2102372	2101656	2102432	60		chromosome partitioning protein
Atu2144	-	-	2110319	2109546	2110351	32	36	hypothetical protein
Atu2155	*metH*	-	2123879	2120106	2123929	50		methionine synthase

Locus tag	Gene name	Strand	Coding region 5' end	Coding region 3' end	TSS	5' UTR length This study	5' UTR length Wilms et al. 2012 [1]	Gene product
Atu2158	-	-	2126724	2126272	2126879	155		hypothetical protein
Atu2159	*omp*	+	2127166	2127795	2127131	35		outer membrane protein
Atu2163	-	-	2129980	2129645	2130035	55		hypothetical protein
Atu2169	-	-	2139804	2139352	2139851	47	266	hypothetical protein
Atu2170	*carA*	+	2140140	2141345	2140057	83		carbamoylphosphate synthase small chain
Atu2175	-	-	2149015	2148098	2149056	41	41	hypothetical protein
Atu2181	-	-	2158574	2157342	2158574	0		hypothetical protein
Atu2187	-	+	2163657	2163947	2163609	48		transcriptional regulator, ArsR family
Atu2193	-	-	2168257	2167991	2168355	98	98	hypothetical protein
Atu2196	*aatA*	-	2170821	2169619	2170864	43		aspartate aminotransferase A
Atu2198	-	+	2172107	2172418	2172080	27		hypothetical protein
Atu2200	*cspA*	+	2173922	2174137	2173700	222	222	cold shock protein
Atu8172†	-	-	2180250	2179750	2180407	157		hypothetical protein
Atu8173	-	-	2180940	2180677	2180989	49	49	hypothetical protein
Atu2218	*prsA*	+	2186332	2187264	2186239	93	123	ribose-phosphate pyrophosphokinase
Atu2224	*aldA*	-	2195107	2193590	2195150	43		aldehyde dehydrogenase
Atu2227†	*rplY*	+	2198732	2199340	2198627	105		50S ribosomal protein L25
Atu2232	*clpS*	-	2204396	2204085	2204455	59	79	ATP-dependent Clp protease adaptor protein ClpS
Atu2239	*fbcF*	-	2210565	2209987	2210679	114		ubiquinol-cytochrome C reductase iron-sulfur subunit
Atu2242†	-	-	2215719	2213869	2215957	238	238	ABC transporter, nucleotide binding/ATPase protein
Atu2248	-	+	2221017	2221283	2220991	26	26	hypothetical protein
Atu2268	*sigD*	-	2242481	2241972	2242802	321		ECF family sigma factor
Atu2270	*clpP*	-	2243652	2243071	2243698	46		ATP-dependent Clp protease, proteolytic subunit
Atu2276	*braC*	-	2248495	2247389	2248611	116		ABC transporter, substrate binding protein (branched chain amino acid)
Atu2278	*nolR*	-	2250397	2250077	2250498	101		transcriptional regulator, ArsR family
Atu2281	-	-	2254078	2253125	2254102	24		ABC transporter, substrate binding protein (proline/glycine betaine)
Atu2283	-	+	2255101	2255553	2255060	41		pseudoazurin
Atu2286	-	-	2258310	2257789	2258369	59		hypothetical protein
Atu2287	-	+	2258667	2260907	2258640	27	101	outer membrane heme receptor
Atu2317	-	-	2295875	2295279	2295886	11		hypothetical protein
Atu2320*	-	-	2298291	2297707	2298279	-12		transcriptional regulator, TetR family; N-terminus is 4 aa longer than homologs.
Atu2321	-	+	2298457	2299752	2298386	71		D-alanyl-D-alanine carboxypeptidase (penicillin binding protein)
Atu2335	-	-	2313679	2313284	2313845	166		hypothetical protein
Atu2354	-	-	2329612	2329106	2329773	161		transcriptional regulator, MarR family
Atu2374*	-	-	2347954	2347613	2347902	-52		hypothetical protein; N-terminus is 31 aa longer than homologs.
Atu2380	-	+	2352777	2353178	2352758	19		hypothetical protein
Atu2383	-	-	2354243	2353818	2354300	57	57	hypothetical protein
Atu2384	-	-	2355046	2354405	2355196	150		transcriptional regulator, TetR family
Atu2392	-	+	2362835	2364028	2362638	197	198	HlyD family secretion protein
Atu2394	*sinR*	+	2367708	2368397	2367585	123	123	Regulator of Biofilm formation, Fnr Family
Atu2409	-	-	2379182	2377926	2379296	114	116	hypothetical protein
Atu2415	-	-	2385994	2385797	2386050	56		glutamine synthetase translation inhibitor
Atu2416	*glnA*	+	2386484	2387518	2386442	42		glutamine synthetase
Atu8177	-	-	2396095	2395742	2396202	107		hypothetical protein
Atu2427	*livH*	-	2400188	2399286	2400248	60	81	ABC transporter, membrane spanning protein
Atu2428	*pssB*	+	2400483	2401325	2400446	37		exopolysaccharide production protein PssB

Locus tag	Gene name	Strand	Coding region 5' end	Coding region 3' end	TSS	5' UTR length This study	5' UTR length Wilms et al. 2012 [1]	Gene product
Atu2430	-	+	2402896	2403171	2402866	30		hypothetical protein
Atu2434	*ctrA*	+	2405136	2405840	2405104	32		two component response regulator
Atu2439	-	+	2409211	2409801	2409061	150		COG5400 hypothetical protein
Atu2447	*purA*	-	2420226	2418928	2420302	76		adenylosuccinate synthetase
Atu2450	-	-	2422307	2421984	2422413	106	125	hypothetical protein
Atu2454	-	-	2426342	2425266	2426360	18	18	iron-chelator utilization protein
Atu2456	*lguL*	-	2427540	2427145	2427587	47		lactoylglutathione lyase
Atu2460	*hmuT*	-	2430977	2430084	2431026	49		ABC transporter, substrate binding protein (hemin)
Atu2461	-	+	2431164	2431511	2431137	27		hypothetical protein
Atu2466	-	-	2436258	2435578	2436273	15		two component response regulator
Atu2469	-	+	2437809	2438753	2437606	203	214	hypothetical protein
Atu2477	*dps*	+	2445403	2445891	2445330	73		DNA oxidation protective protein, not DNA-binding
Atu2484	-	+	2453426	2453620	2453382	44	44	hypothetical protein
Atu2487	*pdxK*	+	2456221	2457096	2456201	20		pyridoxamine kinase
Atu2502	*hemO*	-	2471077	2470466	2471100	23		heme oxygenase
Atu2503	*dapA*	+	2471384	2472268	2471363	21		dihydrodipicolinate synthase
Atu2550	-	+	2521024	2522226	2520851	173		RND multidrug efflux membrane permease
Atu2552	*nodT*	+	2525519	2526973	2525471	48		nodulation protein T precursor
Atu2557†	-	+	2530420	2530608	2530190	230		hypothetical protein
Atu2561	*modA*	+	2533873	2534664	2533839	34		ABC transporter, substrate binding protein (molybdate)
Atu2569	*thiC*	-	2541730	2539907	2541935	205	205	thiamine biosynthesis protein; TPP riboswitch (RF00059)
Atu2604	-	-	2584466	2583285	2584671	205		oxidoreductase
Atu2613†	*hemA*	-	2593201	2592158	2593478	277	115	5-aminolevulinate synthase
Atu2614	-	+	2593746	2593997	2593532	214		hypothetical protein
Atu2615	-	+	2594173	2596098	2594116	57	57	hypothetical protein
Atu2617	*cheW*	+	2600470	2600949	2600366	104	115	chemotaxis protein
Atu2625	*atpH*	-	2609194	2608634	2609334	140		ATP Synthase delta chain
Atu2639	-	-	2625757	2624795	2625812	55		putative malate dehydrogenase
Atu2641	-	-	2627734	2627192	2627764	30		hypothetical protein
Atu2645	*sdhC*	-	2631369	2630977	2631444	75		succinate dehydrogenase cytochrome B-556 subunit
Atu2646	-	+	2631666	2632043	2631648	18		hypothetical protein
Atu2655	-	+	2637429	2638874	2637405	24	24	COG4223 hypothetical protein
Atu2660	-	+	2643260	2643811	2643209	51	68	hypothetical protein
Atu2661	*ppa*	-	2644350	2643817	2644380	30		inorganic pyrophosphatase
Atu2662	-	-	2645026	2644520	2645108	82		acetyltransferase
Atu2663	*typA*	-	2647062	2645242	2647117	55		GTP-binding tyrosin phosphorylated protein
Atu2667	*argG*	-	2652458	2651235	2652480	22		argininosuccinate synthase
Atu2668	-	+	2652774	2653628	2652671	103		oxidoreductase
Atu2678	*ligT*	-	2661331	2660738	2661360	29	29	2'-5' RNA ligase
Atu2682	-	+	2665628	2666410	2665550	78	78	hypothetical protein; ybhL leader (RF00520)
Atu2683	-	-	2666926	2666534	2666954	28	28	hypothetical protein
Atu2684	-	-	2667963	2667298	2668098	135		hypothetical protein
Atu2685†	*acnA*	-	2670922	2668229	2671071	149	170	aconitate hydratase
Atu2700	*rimM*	+	2684495	2685034	2684442	53		16S rRNA processing protein
Atu2703	*rplS*	+	2686734	2687273	2686617	117		50S ribosomal protein L19
Atu8028*	-	+	2693384	2693518	2693396	-12	-13	hypothetical protein
Atu2711	-	-	2696771	2696595	2696776	5		hypothetical protein
Atu2723	*gcpE*	-	2709467	2708217	2709585	118		4-hydroxy-3-methylbut-2-en-1-yl diphosphate synthase
Atu2726	*pycA*	-	2715180	2711719	2715308	128		pyruvate carboxylase

Locus tag	Gene name	Strand	Coding region 5' end	Coding region 3' end	TSS	5' UTR length This study	5' UTR length Wilms et al. 2012 [1]	Gene product
Atu2728	*chvA*	+	2716357	2718123	2716267	90		beta (1-->2) glucan export ATP-binding protein
Atu2730†	*chvB*	-	2727333	2718838	2727756	423		beta (1-->2) glucan biosynthesis protein
Atu2733	-	-	2729343	2729155	2729345	2		hypothetical protein
Atu2746	-	+	2744507	2745193	2744395	112	103	hypothetical protein
Atu2751†	*fdxA*	-	2750726	2750388	2750830	104		ferredoxin
Atu2753	*mgpS*	-	2754403	2751305	2754474	71		COG0513LKJ ATP-dependent helicase
Atu2760	-	+	2761964	2762638	2761931	33	16	hypothetical protein
Atu2763	*mtrA*	-	2764896	2764213	2764975	79		two component response regulator
Atu2765†	-	-	2766225	2765656	2766464	239	240	transcriptional regulator, CarD family
Atu2766	*gdh*	+	2766767	2771524	2766725	42		NAD-glutamate dehydrogenase
Atu2768	-	-	2773636	2773061	2773692	56		hypothetical protein
Atu2769	*phbA*	+	2773879	2775060	2773860	19		acetyl-CoA acetyltransferase; poly-beta-hydroxybutyrate biosynthesis
Atu2771	*bfr*	+	2776477	2776986	2776463	14		bacterioferritin
Atu2777*	-	-	2782854	2782366	2782835	-19		hypothetical protein
Atu2785	*rplU*	-	2789234	2788920	2789378	144		50S ribosomal protein L21
Atu2786	-	+	2789567	2790007	2789551	16	17	hypothetical protein
Atu2789	-	+	2791250	2791480	2791182	68	18	hypothetical protein
Atu2806	-	-	2809883	2809425	2810155	272	291	hypothetical protein; Cobalamin riboswitch (RF00174)
Atu2826	*htpx*	-	2832238	2831273	2832284	46		protease heat shock protein
Atu2827	-	+	2832355	2832567	2832312	43		hypothetical protein
Atu2833†	*rho*	-	2839612	2838347	2839721	109	109	transcription termination factor Rho
**Linear chromosome**								
Atu3014	-	-	9604	9341	9666	62	-5	hypothetical protein
Atu3015	-	+	9998	10549	9742	256		hypothetical protein
Atu3019	-	-	12239	11703	12356	117		hypothetical protein
Atu3077	*ampC*	-	84123	82966	84178	55		beta-lactamase
Atu3091	-	+	97368	98270	97348	20		hypothetical protein
Atu3121	*cspA*	+	131190	131399	131067	123	124	cold shock protein
Atu3140	-	-	149055	148144	149116	61		5-dehydro-4-deoxyglucarate dehydratase
Atu3185†	*ugpB*	+	194587	195906	194509	78		glycerol-3-phosphate ABC transporter substrate-binding protein
Atu3191	-	-	201242	200556	201331	89		outer membrane protein
Atu3196	*speF*	-	205382	204249	205784	402		ornithine decarboxylase; speF leader (RF00518)
Atu3198	-	-	207838	206885	207907	69		ribose ABC transporter substrate-binding protein
Atu3204*	-	-	217715	216138	217701	-14		diguanylate cyclase; N-terminus is upto 30 aa longer than homologs.
Atu3206	-	-	219772	218729	219792	20		hypothetical protein
Atu3222	-	+	235845	236804	235816	29		ribose ABC transporter substrate-binding protein
Atu3252	*exuR*	+	269049	269765	268989	60		GntR family transcriptional regulator
Atu3292	*alr*	+	317717	318883	317695	22	22	alanine racemase
Atu3295	-	+	321087	321599	321044	43		hypothetical protein
Atu3301	*fixR*	-	329244	328525	329294	50		COG1028IQR short-chain alcohol dehydrogenase
Atu3327	*exoY*	-	361776	361096	361876	100		succinoglycan exopolysaccharide synthesis protein
Atu3332	-	-	368317	367331	368450	133		hypothetical protein
Atu3336	-	-	371021	370824	371085	64		hypothetical protein
Atu3338	*thuE*	+	372478	373743	372420	58		trehalose/maltose ABC transporter substrate-binding protein
Atu3354	-	-	391253	388845	391351	98		pyrroloquinoline-quinone-dependent quinate dehydrogenase

Locus tag	Gene name	Strand	Coding region 5' end	Coding region 3' end	TSS	5' UTR length This study	5' UTR length Wilms et al. 2012 [1]	Gene product
Atu3357	-	+	395279	396118	395264	15		amino acid ABC transporter substrate-binding protein
Atu8094	-	-	405588	405343	405704	116		hypothetical protein
Atu3368	-	-	409510	408413	409574	64		periplasmic mannitol-binding protein
Atu3384	-	-	427538	426783	427583	45		hypothetical protein
Atu3392	-	+	436675	437499	436502	173		methyltransferase
Atu3413	*lrp*	-	460609	460133	460661	52		AsnC family transcriptional regulator
Atu3423	-	+	470628	471077	470577	51		hypothetical protein
Atu3475*	-	-	523862	522624	523857	-5		acetyl-CoA C-acetyltransferase; N-terminus is 41 aa longer than homologs.
Atu3496	-	-	548419	547913	548602	183		hypothetical protein
Atu3504	-	-	555612	554626	555634	22		sulfate ABC transporter substrate-binding protein
Atu3505	-	-	556834	555782	556878	44		3-hydroxyisobutyryl-CoA hydrolase
Atu3506*	-	+	556991	557392	557015	-24		hypothetical protein; N-terminus is 48 aa longer than homologs.
Atu3507	-	-	558241	557462	558378	137		hypothetical protein
Atu3519*	*ppiD*	-	567014	566148	567012	-2		peptidyl-prolyl cis-trans isomerase
Atu3525	-	-	575757	575095	575807	50		hypothetical protein
Atu3532	-	-	582314	581565	582344	30		GntR family transcriptional regulator
Atu3563	*greB*	-	616778	616296	616859	81		transcription elongation factor regulatory protein
Atu3564	*exsH*	+	617050	618447	617006	44	44	endo-1,3-1,4-beta-glycanase
Atu3576	*xylF*	-	630869	629829	630960	91		xylose ABC transporter substrate-binding protein
Atu3585	-	-	641218	640322	641271	53		glutathione S-transferase
Atu3586	-	-	642854	641322	642867	13		propionyl-CoA carboxylase
Atu3595	*etfB*	+	649675	650421	649556	119		electron transfer flavoprotein subunit beta
Atu3598	*dsbE*	-	653087	652419	653176	89		thiol:disulfide interchange protein
Atu3603	-	-	659833	659474	659880	47		two component response regulator
Atu3604	*hpt*	-	660518	659976	660570	52		hypoxanthine phosphoribosyltransferase
Atu3606	*ftsE*	+	661462	662121	661418	44		cell division ATP-binding protein
Atu3617	*rpmB*	+	671971	672261	671744	227	90	50S ribosomal protein L28
Atu3619	-	+	673177	673512	673152	25		hypothetical protein
Atu3631[†]	-	+	683199	684338	682930	269	286	hypothetical protein
Atu3635	-	+	687389	688552	687334	55		amidohydrolase
Atu3642	*vgrG*	-	697205	694941	697284	79		vgrG protein
Atu3666	-	+	715046	715504	714999	47		hypothetical protein
Atu3673	-	-	729447	727195	729575	128	130	siderophore biosynthesis protein
Atu3675	-	+	729769	734331	729630	139		peptide synthetase, siderophore biosynthesis protein
Atu3687	-	+	760897	763335	760844	53		TonB-dependent receptor
Atu3692	-	+	767474	768016	767289	185		sigma factor
Atu3704	-	-	783610	782420	783761	151		hypothetical protein
Atu3705[†]	-	-	784696	784049	785137	441		hypothetical protein
Atu3707	*serC*	-	788211	787033	788250	39		phosphoserine aminotransferase; serC leader (RF00517)
Atu3708	-	-	789270	788425	789329	59	58	outer surface protein
Atu3709	*glmM*	-	790811	789459	790879	68		phosphoglucosamine mutase
Atu3710	*ftsH*	-	792946	791000	792990	44	47	metalloprotease
Atu3712	-	-	795446	794457	795531	85		hypothetical protein
Atu3717	*tolQ*	-	800092	799373	800264	172	122	tolQ protein
Atu3724	*ruvC*	-	804615	804103	804628	13	0	Holliday junction resolvase
Atu3728	-	+	809350	809853	809304	46		hypothetical protein
Atu3733	-	-	813851	813639	814008	157		hypothetical protein

Locus tag	Gene name	Strand	Coding region 5' end	Coding region 3' end	TSS	5' UTR length This study	5' UTR length Wilms et al. 2012 [1]	Gene product
Atu8192	-	-	814749	814477	814871	122		hypothetical protein
Atu3736	*tktA*	+	815073	817055	815031	42		transketolase
Atu3740	-	+	821772	822797	821618	154	182	fructose bisphosphate aldolase
Atu3742	-	+	825027	825542	824956	71		COG5317 hypothetical protein
Atu3743	*rpmE*	-	825917	825696	826044	127	73	50S ribosomal protein L31
Atu3747	-	-	831869	830847	831934	65		hypothetical protein
Atu3752	-	-	835945	835769	836007	62	62	COG5570 hypothetical protein
Atu8067	*rpmJ*	+	838132	838257	838050	82	83	50S ribosomal protein L36
Atu3758	-	-	839305	839048	839390	85		hypothetical protein
Atu3761	-	+	840702	841241	840501	201		hypothetical protein
Atu3764	-	-	846383	844842	846407	24		sigma 54 dependent transcriptional activator
Atu3769	-	+	851898	852272	851795	103	36	outer membrane lipoprotein
Atu3770	-	+	852488	854398	852392	96		5'-nucleotidase
Atu3778	*galU*	-	861712	860825	862114	402		UTP-glucose-1-phosphate uridylyltransferase
Atu3779	*mltB*	+	861908	863128	861843	65		lytic murein transglycosylase
Atu3806	*mdeA*	-	904071	902788	904103	32		methionine gamma-lyase
Atu3813	*fabG*	+	909271	910017	909249	22		COG1028IQR 3-oxoacyl-ACP reductase
Atu3816†	*fdh*	-	912375	911329	912599	224		D-threo-aldose 1-dehydrogenase
Atu3817	-	+	912674	914185	912627	47	47	dehydratase
Atu3824	-	-	921407	920424	921525	118		hypothetical protein
Atu3826	-	+	922011	922841	921718	293	293	acyl-CoA hydrolase; Cobalamin riboswitch (RF00174)
Atu3835	-	-	932067	931282	932132	65		hypothetical protein
Atu3861	-	-	951403	951014	951431	28		acyl-CoA hydrolase
Atu3864	-	-	953697	953425	954092	395		hypothetical protein
Atu3873*	-	+	966601	967581	966617	-16		hypothetical protein; N-terminus is 13 aa longer than homologs.
Atu3883	-	-	978478	978116	978565	87		chemotaxis response regulator
Atu3886	*pldB*	-	981601	980660	981634	33		lysophospholipase
Atu3887	*hspL*	+	981981	982463	981890	91		small heat shock protein
Atu3897	*glpD*	-	993791	992268	993837	46		glycerol-3-phosphate dehydrogenase
Atu3909	-	+	1008352	1009593	1008286	66	47	hypothetical protein
Atu3924	*repA*	-	1026575	1025361	1026621	46		replication protein A
Atu3961	-	-	1066093	1065344	1066102	9		GntR family transcriptional regulator
Atu3963	-	-	1068233	1067535	1068248	15		GntR family transcriptional regulator
Atu3964	-	+	1068391	1069404	1068332	59		dehydrogenase
Atu8195*	-	-	1108294	1107884	1108291	-3	2	hypothetical protein
Atu3997	*bioB*	+	1111072	1112091	1111038	34		biotin synthetase
Atu4007	*arcA*	+	1119314	1120234	1119289	25	25	arginase
Atu4014	*exsI*	-	1127097	1126867	1127200	103		transcriptional regulator
Atu4026	-	+	1141762	1142289	1141625	137		hypothetical protein
Atu4033	-	-	1148869	1147853	1148976	107	108	sugar ABC transporter substrate-binding protein
Atu4034	-	+	1149178	1150413	1149008	170	74	two component sensor kinase
Atu4039*	-	+	1153658	1153948	1153745	-87		hypothetical protein; N-terminus is 35 aa longer than homologs.
Atu4046	-	-	1160337	1159339	1160377	40		glycine betaine ABC transporter substrate-binding protein
Atu4056	*exoH*	-	1174304	1173156	1174390	86	91	succinoglycan biosynthesis protein
Atu4058	*exoW*	+	1176272	1177237	1176139	133		succinoglycan biosynthesis protein
Atu4064	*rpsU*	-	1181811	1181584	1181901	90		30S ribosomal protein S21
Atu4078	*glgP*	-	1200318	1197856	1200380	62		glycogen phosphorylase

Locus tag	Gene name	Strand	Coding region 5' end	Coding region 3' end	TSS	5' UTR length This study	5' UTR length Wilms et al. 2012 [1]	Gene product
Atu4081	-	-	1202287	1201574	1202313	26		aspartate racemase
Atu4095	-	-	1215363	1215112	1215424	61		hypothetical protein
Atu8037	-	-	1221727	1221338	1221799	72		COG4530 hypothetical protein
Atu4123	-	+	1241719	1242927	1241611	108		branched chain amino acid ABC transporter substrate-binding protein
Atu4136	-	-	1256701	1256201	1256749	48		hypothetical protein
Atu4143	-	-	1261246	1261025	1261393	147		hypothetical protein
Atu4153	-	+	1270148	1271677	1270035	113		aldehyde dehydrogenase
Atu4154	-	+	1271907	1272860	1271868	39		aliphatic sulfonate ABC transporter substrate-binding protein
Atu4161	-	-	1279712	1279506	1279737	25		hypothetical protein
Atu4162	-	+	1279850	1280644	1279816	34		two-component response regulator
Atu4164	-	-	1283729	1281990	1283742	13		two component sensor kinase
Atu4165	-	+	1283944	1284519	1283901	43		hypothetical protein
Atu4168	-	-	1286665	1286219	1286777	112		hypothetical protein
Atu4173	*ptsI*	+	1293152	1295419	1293096	56	61	phosphoenolpyruvate-protein phosphotransferase
Atu4176†	-	+	1297793	1298578	1297495	298		hypothetical protein
Atu4177	*clpB*	+	1298943	1301567	1298779	164		ATP-dependent Clp protease, ATP-binding subunit
Atu4192	-	+	1313719	1315668	1313623	96		oligopeptide ABC transporter substrate-binding protein
Atu4214	*cspA*	-	1337029	1336799	1337029	0		cold shock protein
Atu4259	-	-	1386341	1384746	1386361	20		oligopeptide ABC transporter substrate-binding protein
Atu4299	-	+	1423945	1424484	1423945	0		hypothetical protein
Atu4319	-	+	1447208	1448092	1447109	99		AraC family transcriptional regulator
Atu4329	*bexD*	+	1457455	1458357	1457195	260		polysaccharide export protein
Atu4343	*impA*	-	1477039	1475984	1477100	61	65	hypothetical protein
Atu4344	*clpB*	+	1477260	1479938	1477221	39		ATP-dependent Clp protease, ATP-binding subunit
Atu4368	-	-	1505385	1504990	1505409	24		hypothetical protein
Atu4380	*nnrR*	-	1518115	1517414	1518186	71		Crp family transcriptional regulator
Atu4405	*napE*	+	1541384	1541569	1541362	22	22	periplasmic nitrate reductase NapE
Atu4418	*gcdH*	-	1560730	1559543	1560750	20		glutaryl-CoA dehydrogenase
Atu4420	-	+	1561882	1562943	1561858	24	28	LacI family transcriptional regulator
Atu4443	-	+	1587355	1587705	1587339	16		hypothetical protein
Atu4447	-	+	1592333	1593646	1592309	24		sorbitol ABC transporter substrate-binding protein
Atu4458	-	+	1603260	1603742	1603210	50	51	hypothetical protein
Atu4464	*talB*	+	1606687	1607652	1606510	177	97	transaldolase B
Atu4471	*sitA*	-	1613387	1612488	1613405	18		ABC transporter substrate-binding protein
Atu4472	-	+	1613672	1614580	1613567	105		hypothetical protein
Atu4476	-	-	1621017	1620256	1621058	41		hypothetical protein
Atu4493	-	-	1641077	1640589	1641095	18	19	hypothetical protein
Atu4499*	-	-	1645778	1645011	1645685	-93		hypothetical protein; N-terminus is 42 aa longer than homologs.
Atu4504	-	+	1650895	1651404	1650832	63		MarR family transcriptional regulator
Atu4600	-	-	1747484	1745721	1747712	228		ABC transporter permease
Atu4643	-	-	1796744	1796568	1796806	62		hypothetical protein
Atu4647	-	+	1800329	1801282	1800311	18		hypothetical protein
Atu4673†	*ftsZ1*	+	1832465	1833427	1832224	241		cell division protein FtsZ
Atu4678	-	+	1836914	1837720	1836879	35		amino acid ABC transporter substrate-binding protein
Atu4706	*nuoE*	+	1867324	1867803	1867248	76	76	formate dehydrogenase subunit gamma
Atu8200	-	+	1888598	1888918	1888576	22	22	hypothetical protein
Atu4727	-	+	1889922	1890911	1889892	30		hypothetical protein

Locus tag	Gene name	Strand	Coding region		TSS	5' UTR length		Gene product
			5' end	3' end		This study	Wilms et al. 2012 [1]	
Atu4728†	*exsA*	+	1891371	1893173	1891118	253		ABC transporter permease
Atu4733	-	-	1899541	1899044	1899600	59		hypothetical protein
Atu4734	*acnB*	+	1899905	1902694	1899811	94		bifunctional aconitate hydratase 2/2-methylisocitrate dehydratase
Atu4744	-	+	1913329	1914183	1913060	269		sugar ABC transporter substrate-binding protein
Atu4769	-	-	1938524	1937805	1938634	110		hypothetical protein
Atu4780	-	+	1949708	1950796	1949558	150		hypothetical protein
Atu4782	*drrA*	-	1953847	1953101	1953862	15		two component response regulator
Atu4783	*afuA*	+	1954065	1955093	1954030	35	35	ABC transporter substrate-binding protein
Atu8042*	-	+	1998786	1999451	1998858	-72		hypothetical protein; N-terminus is 33 aa longer than homologs.
Atu4831	-	-	2011265	2010876	2011277	12		hypothetical protein
Atu4842	-	+	2021679	2022725	2021459	220		sugar ABC transporter substrate-binding protein
Atu4856	-	+	2040246	2041172	2039777	469		nucleotidyltransferase
Atu8201	-	+	2064459	2064656	2064393	66	29	hypothetical protein
Atu4888	-	+	2065436	2065726	2065410	26		hypothetical protein

pAt plasmid

Atu8044	-	+	10306	10611	10286	20	20	hypothetical protein
Atu5013	-	+	14416	14664	14400	16		hypothetical protein
Atu5052	*hspAT1*	+	51311	51787	51233	78		small heat shock protein
Atu5071	*dppA*	-	71549	70032	71690	141		ABC transporter substrate binding protein (dipeptide)
Atu5072	-	+	71901	73091	71887	14		hypothetical protein
Atu5089	-	+	92148	93263	92131	17		hypothetical protein
Atu5091	*rcdB*	-	96448	95255	96535	87		Curdlan synthesis protein
Atu5125	*atrA*	-	133054	132365	133166	112		GntR family transcriptional regulator
Atu5136	*blcR*	-	143179	142352	143207	28	28	transcriptional repressor of the blcABC operon
Atu5137	*blcA*	+	143235	144689	143190	45		NAD-dependent succinyl-semialdehyde dehydrogenase
Atu5148	*attW*	-	152899	152495	152923	24		hypothetical protein
Atu5159	*atsE*	-	159149	158745	159205	56		hypothetical protein
Atu8203	-	+	171061	171345	171013	48		hypothetical protein
Atu5449	*hspAT2*	+	442069	442545	441990	79		heat-shock protein
Atu5467	-	+	461363	461674	461290	73	93	hypothetical protein
Atu5469	*dinP*	+	461941	463014	461887	54	55	DNA polymerase IV
Atu5484	-	-	476673	475927	476706	33	43	hypothetical protein
Atu5487	-	+	477832	478152	477748	84		hypothetical protein
Atu5489	-	+	479072	479299	479025	47		hypothetical protein
Atu5493	-	-	483498	483181	483566	68		hypothetical protein
Atu5516	-	-	508975	507596	509021	46	52	hypothetical protein
Atu5543	-	+	537883	538530	537729	154		AraC family transcriptional regulator

Ti plasmid

Atu6043	*repA*	+	52233	53450	52203	30		plasmid-partitioning protein RepA
Atu6050	-	+	62881	63597	62795	86		hypothetical protein
Atu6082	-	+	97822	98859	97755	67		AAA ATPase family protein
Atu6091	-	+	109282	110034	109222	60	61	COG4544 hypothetical protein
Atu6114	-	-	135138	134896	135163	25		hypothetical protein
Atu6131†	*traM*	+	150171	150479	149937	234	234	TraR antiactivator
Atu6150	*virH1*	+	167883	169142	167798	85		P-450 monooxygenase
Atu6154†	*virF*	+	172489	173427	172067	422	27	exported virulence protein
Atu6155†	-	+	173878	174306	173671	207		hypothetical protein

Locus tag	Gene name	Strand	Coding region 5' end	3' end	TSS	5' UTR length This study	Wilms et al. 2012 [1]	Gene product
Atu6164	*tzs*	-	179993	179262	180071	78		trans-zeatin secretion protein
Atu6166	*virA*	+	180831	183332	180595	236		two-component VirA-like sensor kinase
Atu6167	*virB1*	+	183877	184614	183812	65	65	type IV secretion system lytic transglycosylase VirB1
Atu6178	*virG*	+	193546	194307	193456	90	60	two-component response regulator VirG
Atu6180	*virC1*	-	195755	195060	195825	70		putative crown gall tumor protein VirC1
Atu6181	*virD1*	+	196026	196469	195961	65		type IV secretion system T-DNA border endonuclease VirD1
Atu6188	*virE0*	+	205684	205935	205615	69		virA/G regulated protein

[1] Wilms et al. Deep sequencing uncovers numerous small RNAs on all four replicons of the plant pathogen *Agrobacterium tumefaciens* . RNA Biology 2012, 9:446-457
*TSS mapped within the coding sequence.
†Includes candidate sRNAs identified by Wilms et al. [1].

Table S3-A. List of identified candidate ncRNAs on the circular chromosome. Highly expressed putative ncRNAs were identified when the average depth of coverage of the non-gene-coding region was at least 10 times higher than that in adjacent regions. ncRNA tag was named with the replicon (C1, circular chromosome; C2, linear chromosome; pAt, pAt plasmid; pTi, plasmid Ti) and 5' End position with strand information (F, forward strand; R, reverse strand). RPKM (Reads Per Kilobase per Million mapped reads) in 8 different cDNA libraries were listed along with the information of genes encoded on the opposite strand.

	Circular chromosome	Strand	ncRNA position		RPKM (-TEX)				RPKM (+TEX)				
	ncRNA name		5' end	3' end	YEP-L	YEP-S	AB	IND	YEP-L	YEP-S	AB	IND	antisense to
1	C1_8788R	-	8788	8649	0	0	7	0	23	7	43	24	*Atu0012 (gyrB)*
2	C1_9609R	-	9609	9263	5	28	31	4	89	67	114	55	*Atu0012 (gyrB)*
3	C1_31881F	+	31881	32237	10	23	17	11	39	41	40	26	*Atu0029 (ahcY)*
4	C1_34272F	+	34272	34352	0	0	21	25	23	11	27	85	*Atu0032*
5	C1_41617F	+	41617	41785	0	0	10	0	19	59	45	14	*Atu0039 (hisF)*
6	C1_70266R	-	70266	70047	5	28	59	25	58	81	110	41	*Atu0066*
7	C1_72361R	-	72361	72248	5	0	0	0	105	59	47	14	*Atu0070 (nrdE)*
8	C1_101545R[*†]	-	101545	101446	10697	14477	13806	9254	6999	5692	3723	3674	*Small SRP*
9	C1_109477F[*†]	+	109477	109594	1503	413	15354	24677	16287	3009	37950	34389	*Atu0105*
10	C1_109596F	+	109596	109822	719	377	868	802	1346	1098	1443	1275	*Thermosensor*
11	C1_110258F[*]	+	110258	110380	5420	5444	2955	3636	559	381	220	983	*Intergenic*
12	C1_112676R[*†]	-	112676	112535	541	402	819	8686	5028	3404	3819	23172	*Intergenic*
13	C1_166980R	-	166980	166594	5	11	3	0	35	67	45	14	*Atu0161 (exbB)*
14	C1_204292F	+	204292	204472	0	0	14	0	39	41	45	16	*Atu0200*
15	C1_227050F	+	227050	227161	10	11	0	25	128	59	58	37	*Atu0221 (ctpD)*
16	C1_228801F	+	228801	228927	0	0	49	18	58	44	65	49	*Atu0222 (ctpC)*
17	C1_232821R	-	232821	232747	5	0	0	0	85	44	31	24	*Atu0231 (guaA)*
18	C1_241360F	+	241360	241786	0	6	10	4	23	48	81	20	*Atu0241 (lepA)*
19	C1_247797F	+	247797	247930	0	11	52	0	124	137	168	45	*Atu0249*
20	C1_256373R	-	256373	256110	0	0	17	7	93	92	148	49	*Atu0259 (pheT)*
21	C1_293634F	+	293634	293992	15	119	14	18	50	251	20	24	*Atu0301 (dnaN)*
22	C1_298719R	-	298719	298560	69	45	90	60	128	118	63	59	*Atu0306 (ugpA)*
23	C1_300504R	-	300504	300365	20	85	83	11	43	41	83	24	*Atu0308 (ugpC)*
24	C1_326637F	+	326637	326841	15	28	42	14	113	92	110	37	*Atu0332 (rpoN)*
25	C1_327010F	+	327010	327131	0	11	24	75	0	4	25	85	*Atu0333*
26	C1_367107R	-	367107	366893	0	0	28	4	66	67	96	43	*Atu0373*
27	C1_410585F	+	410585	410748	64	28	111	39	8	0	20	14	*Intergenic*
28	C1_423383R	-	423383	423096	496	600	493	273	384	336	876	483	*Atu0426*
29	C1_423713R[†]	-	423713	423410	1746	1851	1531	881	667	651	1375	794	*Atu0426*
30	C1_433891F	+	433891	434037	35	40	38	39	113	181	141	49	*Atu0438 (intD)*
31	C1_438132F[†]	+	438132	438614	30	34	274	202	27	22	262	187	*Atu0443*
32	C1_439446F	+	439446	439511	45	192	274	224	0	170	103	215	*Intergenic*
33	C1_445306F[*]	+	445306	445498	307	577	538	313	1377	2210	1519	776	*Intergenic*
34	C1_459703R[†]	-	459703	459395	109	164	260	170	206	288	206	150	*Atu0468*
35	C1_471085R	-	471085	470989	0	0	21	21	58	18	49	47	*Atu0481 (exoQ)*
36	C1_500419F	+	500419	500832	0	0	21	7	19	52	72	22	*Atu8131 (phaA)*
37	C1_532642F	+	532642	533033	10	6	28	14	62	78	87	47	*Atu0542 (fla)*
38	C1_541674F	+	541674	542312	0	17	38	21	50	41	72	35	*Atu0555 (flaB); Atu0556*
39	C1_547546R	-	547546	547397	0	6	7	0	54	59	112	47	*Atu0561 (fliM)*
40	C1_562840R	-	562840	562418	15	45	63	36	70	89	112	47	*Atu0576 (flgL)*
41	C1_581234R	-	581234	581152	0	23	49	7	23	22	45	30	*Atu8133*
42	C1_581451R	-	581451	581389	0	0	7	7	0	0	45	12	*Atu8133*
43	C1_581692R	-	581692	581533	0	28	38	14	27	15	43	49	*Atu8133*
44	C1_591560F[†]	+	591560	592001	69	147	76	18	341	492	177	57	*Atu0604*

	Circular chromosome	Strand	ncRNA position		RPKM (-TEX)				RPKM (+TEX)				
	ncRNA name		5' end	3' end	YEP-L	YEP-S	AB	IND	YEP-L	YEP-S	AB	IND	antisense to
45	C1_600635F[†]	+	600635	600750	164	481	385	249	353	699	625	487	*Intergenic*
46	C1_605467F	+	605467	605693	0	6	14	21	66	55	49	28	*Atu0614*
47	C1_665234R[†]	-	665234	665117	109	170	531	252	16	41	78	136	*Intergenic*
48	C1_693513R	-	693513	693457	0	0	0	0	47	0	0	0	*Atu0695 (kdtA)*
49	C1_711354R	-	711354	711136	0	11	24	7	16	63	85	28	*Atu0710 (corA)*
50	C1_717755R	-	717755	717613	0	0	24	14	31	133	164	77	*Atu0716 (atpB); Atu0717 (atpF)*
51	C1_720637R[†]	-	720637	720565	818	1019	13045	11871	159	314	6994	22173	*Intergenic*
52	C1_728732R	-	728732	728635	0	0	0	0	78	55	65	12	*Atu0728*
53	C1_733937R	-	733937	733765	10	23	24	14	19	55	63	20	*Atu0734*
54	C1_782205F	+	782205	782528	0	0	10	0	19	22	54	14	*Atu0784; Atu0785 (rimJ)*
55	C1_792114F	+	792114	792215	1180	1256	507	217	190	163	56	35	*Atu0795*
56	C1_792154F	+	792154	792215	1661	1930	726	327	175	177	58	28	*Atu0795*
57	C1_805001F	+	805001	805137	69	57	101	53	66	118	85	49	*Intergenic*
58	C1_813457R	-	813457	813303	25	28	7	0	244	270	56	26	*Intergenic*
59	C1_813546R[*†]	-	813546	813485	630	1336	1118	2013	70	67	18	114	*Intergenic*
60	C1_813854F[†]	+	813854	814010	60	328	201	167	74	429	379	331	*Intergenic*
61	C1_834982R	-	834982	834598	0	6	0	0	39	52	76	22	*Atu0835 (gst)*
62	C1_852289R	-	852289	852054	268	656	531	380	1020	1674	927	532	*Intergenic*
63	C1_856957R	-	856957	856860	0	0	0	21	66	15	58	30	*Atu0857*
64	C1_883375F	+	883375	883594	40	23	63	4	194	92	94	16	*Intergenic*
65	C1_907861R	-	907861	907800	25	57	21	39	0	52	112	317	*Intergenic*
66	C1_925478R	-	925478	925368	30	0	10	0	182	59	13	18	*Atu0936*
67	C1_942251R[*†]	-	942251	942016	233	1171	830	1080	636	3179	1046	1109	*Intergenic*
68	C1_944979R	-	944979	944834	20	28	0	0	78	129	36	4	*Atu0954 (gp34)*
69	C1_960996R	-	960996	960851	25	17	94	46	128	37	367	128	*Atu0971*
70	C1_982030R[*†§]	-	982030	981727	337	57	3986	5579	962	229	5155	4584	*Atu0986*
71	C1_998095R[†]	-	998095	998034	0	113	497	277	0	30	29	89	*Intergenic*
72	C1_1003889R[†]	-	1003889	1003434	0	11	87	277	12	7	110	357	*Atu1007*
73	C1_1052093R[†]	-	1052093	1051804	600	787	149	366	644	1294	211	443	*5' UTR of Atu1061*
74	C1_1052333F	+	1052333	1052534	5	0	3	7	70	22	27	14	*Atu1062 (pssA)*
75	C1_1070866F	+	1070866	1071043	25	28	59	32	97	81	110	55	*Atu1078; Atu1079*
76	C1_1080618F	+	1080618	1080805	10	74	59	39	47	155	128	81	*Atu1088 (rplI); Atu1089*
77	C1_1121350R	-	1121350	1121278	15	0	104	67	31	11	105	148	*Intergenic*
78	C1_1122440F[†]	+	1122440	1122662	30	141	181	114	213	558	520	201	*Atu1133 (rns)*
79	C1_1127982F	+	1127982	1128192	20	62	28	21	190	211	190	43	*Atu1138*
80	C1_1158155R	-	1158155	1158065	0	0	24	28	43	55	69	47	*Intergenic*
81	C1_1164583R	-	1164583	1164435	0	0	10	14	58	59	65	39	*Atu1170*
82	C1_1173317R	-	1173317	1173058	15	62	90	67	147	310	338	110	*Atu1179 (fabH)*
83	C1_1175776F	+	1175776	1175977	89	74	94	64	369	314	356	118	*Intergenic*
84	C1_1181830R	-	1181830	1181751	0	6	0	0	23	11	40	32	*Atu1188*
85	C1_1182530F	+	1182530	1182608	30	17	0	0	182	52	4	14	*C1_1182425R*
86	C1_1182639R	-	1182639	1182425	5	11	49	28	8	59	38	24	*C1_1182530F*
87	C1_1199763R	-	1199763	1199639	5	6	10	4	12	0	38	18	*Atu1205 (ilvA)*
88	C1_1200302R	-	1200302	1200169	69	57	80	71	113	81	152	102	*Atu1206*
89	C1_1215541R	-	1215541	1215351	40	192	444	295	16	81	323	368	*Atu1222*
90	C1_1216665R[†]	-	1216665	1216249	64	232	802	863	50	229	405	591	*Atu1223*
91	C1_1219871R	-	1219871	1219610	10	23	31	25	105	137	170	59	*Atu8150*
92	C1_1221112R	-	1221112	1220744	89	62	97	71	268	203	215	104	*Atu1230*
93	C1_1226081F	+	1226081	1226180	15	0	38	43	39	48	16	8	*Atu1237*

Circular chromosome		Strand	ncRNA position		RPKM (-TEX)				RPKM (+TEX)				
	ncRNA name		5' end	3' end	YEP-L	YEP-S	AB	IND	YEP-L	YEP-S	AB	IND	antisense to
94	C1_1253630R	-	1253630	1253542	20	40	17	32	229	185	116	51	*Atu1263*
95	C1_1254774R	-	1254774	1254429	79	243	247	181	392	1294	824	307	*Atu1264*
96	C1_1275443R[*†§]	-	1275443	1275297	2713	12965	25899	11016	8574	36917	30473	16440	*Atu1287*
97	C1_1288371R	-	1288371	1288281	25	6	80	50	50	63	67	53	*Atu1298*
98	C1_1288586F[†]	+	1288586	1288696	1736	7046	7156	3782	1121	3223	2251	2443	*Intergenic*
99	C1_1303919F	+	1303919	1304044	253	221	233	128	427	285	329	120	*5' UTR of Atu1311*
100	C1_1329294R	-	1329294	1329188	0	6	24	0	74	81	125	43	*Atu1341 (mrcA)*
101	C1_1332771R	-	1332771	1332672	0	0	0	0	12	15	16	22	*Atu1343*
102	C1_1345805R[*†]	-	1345805	1345651	471	1602	3667	1634	454	1593	7684	4123	*Intergenic*
103	C1_1352134F	+	1352134	1352305	0	0	52	32	70	63	63	61	*Atu1358*
104	C1_1357826R	-	1357826	1357718	0	17	66	18	4	18	45	45	*Intergenic*
105	C1_1370244R	-	1370244	1370071	30	28	28	25	109	96	139	97	*Atu1376 (pyrH)*
106	C1_1375298R	-	1375298	1375192	0	0	28	21	0	0	58	51	*Atu1381 (omp1)*
107	C1_1376106R	-	1376106	1375826	0	0	10	0	4	18	69	16	*Atu1381 (omp1)*
108	C1_1407183F	+	1407183	1407274	0	28	35	25	58	107	148	65	*Atu1412*
109	C1_1429178R	-	1429178	1429058	20	0	45	18	23	44	83	55	*Atu1434 (lpdA)*
110	C1_1449058F	+	1449058	1449327	35	34	63	39	97	33	125	69	*Atu1453*
111	C1_1450700R	-	1450700	1450543	30	85	76	18	151	181	150	57	*Atu1454 (cysG)*
112	C1_1462469R	-	1462469	1462230	3080	2615	2861	1563	11468	8527	7047	3241	*Glycine riboswitch (Atu1464, gcvT)*
113	C1_1464632R[†]	-	1464632	1464500	84	255	309	1392	54	336	152	1202	*Intergenic*
114	C1_1474436R	-	1474436	1474256	79	57	118	82	113	59	74	51	*Intergenic*
115	C1_1488589R	-	1488589	1488446	109	130	181	178	163	89	242	262	*5' UTR of Atu1498 (tmk)*
116	C1_1518052F	+	1518052	1518312	0	0	0	0	12	30	34	14	*Atu1526 (gntZ)*
117	C1_1528040F[†]	+	1528040	1528167	69	238	87	67	136	211	105	97	*Atu1537 (fixN)*
118	C1_1528425F	+	1528425	1528502	0	0	24	46	0	0	20	37	*Intergenic*
119	C1_1533961R[†‡]	-	1533961	1533762	932	1551	670	444	1032	2225	457	256	*Intergenic*
120	C1_1534073R	-	1534073	1534018	25	0	31	60	62	0	20	10	*Intergenic*
121	C1_1536839F	+	1536839	1536902	0	0	59	96	0	0	74	130	*Atu1547 (mgtE)*
122	C1_1581877F	+	1581877	1582033	30	91	90	50	210	240	130	87	*Atu1601 (hemN)*
123	C1_1589675R[†]	-	1589675	1589365	10	108	52	21	39	140	76	28	*Atu1607 (radC)*
124	C1_1612544R	-	1612544	1612372	25	34	52	32	120	133	87	55	*Intergenic*
125	C1_1625426F[†§]	+	1625426	1625895	179	289	368	320	1001	1663	1293	745	*Atu1638*
126	C1_1626061F[†]	+	1626061	1626474	55	119	1247	849	12	30	509	43	*Atu1638; Atu1639*
127	C1_1628381F	+	1628381	1628482	0	0	14	36	0	44	0	26	*Atu1645*
128	C1_1640604R	-	1640604	1640371	64	28	21	11	431	81	74	35	*Cobalamin riboswitch (Atu1654)*
129	C1_1643533F[*]	+	1643533	1643599	84	260	274	298	101	74	246	504	*Intergenic*
130	C1_1653630R	-	1653630	1653531	0	0	0	0	78	44	34	6	*Intergenic*
131	C1_1667597F	+	1667597	1667843	164	17	0	0	493	59	16	8	*Atu1680 (ppiB)*
132	C1_1670494R	-	1670494	1670352	0	0	24	11	66	74	69	16	*Atu1686 (ppiD)*
133	C1_1705208F	+	1705208	1705458	0	23	14	131	66	92	29	236	*Intergenic*
134	C1_1711559R	-	1711559	1711329	79	928	840	497	23	159	179	248	*Intergenic*
135	C1_1713219F[†]	+	1713219	1713417	74	68	170	131	140	251	383	191	*Atu1727*
136	C1_1731976R	-	1731976	1731772	10	0	3	32	132	30	65	77	*Atu1745*
137	C1_1738833F	+	1738833	1738914	5	0	24	28	62	7	36	63	*Atu1752*
138	C1_1745471R[*†]	-	1745471	1745262	213	108	677	646	966	403	1676	851	*Atu1760*
139	C1_1754533R	-	1754533	1754435	0	0	3	4	19	55	45	4	*Atu1770 (glnA)*
140	C1_1755145R	-	1755145	1754986	0	6	7	0	47	67	45	14	*Atu1770 (glnA)*
141	C1_1759857R	-	1759857	1759539	0	0	17	7	47	48	72	26	*Atu1774*

Circular chromosome		Strand	ncRNA position		RPKM (-TEX)				RPKM (+TEX)				
	ncRNA name		5' end	3' end	YEP-L	YEP-S	AB	IND	YEP-L	YEP-S	AB	IND	antisense to
142	C1_1780283F	+	1780283	1780441	40	28	87	71	101	115	146	67	*Atu1793 (pcs)*
143	C1_1792765R[§]	-	1792765	1792443	55	51	97	50	136	229	240	120	*Atu1811; Atu1812*
144	C1_1818346R[†]	-	1818346	1818206	233	300	1007	1090	504	584	979	1338	*Intergenic*
145	C1_1849636R	-	1849636	1849535	30	62	156	46	23	63	65	61	*Intergenic*
146	C1_1883128R	-	1883128	1883063	0	0	0	0	23	48	36	24	*Atu1905*
147	C1_1885370F	+	1885370	1885491	20	23	21	25	62	37	52	67	*Atu1908*
148	C1_1902803F	+	1902803	1902940	0	11	35	14	0	7	34	30	*Atu1924 (rpsK)*
149	C1_1908143F[†]	+	1908143	1908306	30	17	101	46	109	96	128	67	*Atu1931 (rplF); Atu1932 (rpsH)*
150	C1_1941897F	+	1941897	1942028	10	23	14	0	78	11	52	18	*Atu1974 (cfa)*
151	C1_1963614R	-	1963614	1963522	0	11	7	0	50	37	67	14	*Atu2001 (uvrB)*
152	C1_1977199F[†]	+	1977199	1977306	40	147	497	199	39	85	417	276	*Intergenic*
153	C1_1990387R	-	1990387	1990304	25	6	42	57	54	55	85	35	*Atu2030*
154	C1_2003233F	+	2003233	2003465	25	28	59	21	120	133	172	75	*Atu2044 (hflC)*
155	Atu2049[†]	+	2007231	2007563	22440	40606	13906	8665	19088	28960	9019	5372	*tmRNA*
156	C1_2009030F	+	2009030	2009228	74	141	63	50	500	754	323	193	*Intergenic*
157	C1_2009256F	+	2009256	2009424	134	96	59	71	458	303	128	79	*Atu2052*
158	C1_2032975F[†]	+	2032975	2033106	144	28	1104	3619	78	15	318	2193	*Atu2071*
159	C1_2056095F	+	2056095	2056151	0	62	14	4	0	44	56	30	*Atu2093 (murC)*
160	Atu2109[†]	-	2073888	2073552	60912	69740	43229	44272	20652	18237	12679	13050	*rnpB*
161	C1_2085454R	-	2085454	2085321	0	34	42	14	50	63	99	30	*Atu2121 (lyc)*
162	C1_2087200F[*†]	+	2087200	2087384	1661	8580	6413	1243	6545	20617	11237	2333	*Intergenic*
163	C1_2139202F[*†]	+	2139202	2139332	818	1387	1597	7564	644	902	2166	21501	*Intergenic*
164	C1_2143068R	-	2143068	2142759	64	96	135	89	194	218	311	124	*Atu2171*
165	C1_2163256F[*†]	+	2163256	2163370	5043	1273	28677	25589	17172	4036	27964	27289	*suhB (=AbcR1)*
166	Atu8103[†]	+	2163483	2163561	0	23	73	75	0	22	25	12	*suhB (=AbcR2)*
167	C1_2180601F	+	2180601	2180650	0	0	69	25	0	0	0	0	*Intergenic*
168	C1_2194376F	+	2194376	2194558	0	6	21	4	8	33	60	10	*Atu2224 (aldA)*
169	C1_2205841R	-	2205841	2205788	0	11	35	50	4	0	36	63	*Intergenic*
170	C1_2207621F	+	2207621	2207759	0	6	49	28	31	37	31	41	*Intergenic*
171	C1_2208947F	+	2208947	2209298	5	40	73	60	31	159	233	93	*Atu2238 (fbcB)*
172	C1_2235590R	-	2235590	2235469	0	11	0	21	81	115	18	45	*Atu2263*
173	C1_2248157F	+	2248157	2248297	0	6	0	7	12	18	54	30	*Atu2276 (braC)*
174	C1_2250226F	+	2250226	2250306	0	34	21	25	35	15	47	47	*Atu2278 (nolR)*
175	C1_2250953F	+	2250953	2251126	0	0	17	11	43	37	81	47	*Atu2279*
176	C1_2260272R	-	2260272	2260117	5	23	21	0	101	67	92	30	*Atu2287*
177	C1_2273315F[*†]	+	2273315	2273376	16152	21132	14788	16019	70	129	121	288	*Intergenic*
178	C1_2290013R[†]	-	2290013	2289373	620	1013	28	67	248	333	2	30	*Atu2311 (xdhC)*
179	C1_2310423F	+	2310423	2310601	60	28	267	281	450	237	614	516	*5' UTR of Atu2333*
180	C1_2313131F	+	2313131	2313194	10	45	38	18	39	96	65	43	*Intergenic*
181	C1_2315784R	-	2315784	2315470	40	51	35	18	155	214	168	65	*5' UTR of Atu2337*
182	C1_2335684F	+	2335684	2335768	40	23	90	39	101	70	65	49	*Atu2360 (mcpA)*
183	C1_2393362R	-	2393362	2392770	60	74	87	43	264	377	459	132	*Atu2419; Atu2420*
184	C1_2402894R	-	2402894	2402680	0	6	35	4	151	103	56	12	*Atu8178*
185	C1_2404807R	-	2404807	2404219	248	441	201	92	1214	2358	757	256	*Atu2433*
186	C1_2416295F	+	2416295	2416384	278	272	410	291	338	170	298	366	*Intergenic*
187	C1_2437621F	+	2437621	2437761	1572	402	1319	92	1036	336	1660	142	*Intergenic*
188	C1_2446240R[*†]	-	2446240	2445919	476	1551	2583	1261	1032	3072	4164	1903	*Atu2478*
189	C1_2447317F	+	2447317	2447463	55	51	97	46	415	499	524	215	*Atu2480*
190	C1_2452151R	-	2452151	2451987	15	0	3	4	54	70	49	28	*Atu2483 (mexF)*

Circular chromosome		Strand	ncRNA position		RPKM (-TEX)				RPKM (+TEX)				
	ncRNA name		5' end	3' end	YEP-L	YEP-S	AB	IND	YEP-L	YEP-S	AB	IND	antisense to
191	C1_2458707F	+	2458707	2458871	10	6	14	32	85	177	63	169	*Atu2489*
192	C1_2479274R	-	2479274	2478800	35	51	111	85	124	266	385	177	*Atu2510*
193	C1_2494680R	-	2494680	2494597	0	0	24	0	16	15	31	35	*Atu2523 (telA)*
194	C1_2496327R[†]	-	2496327	2496228	40	147	142	50	50	59	72	28	*Atu2524*
195	C1_2523992R	-	2523992	2523809	30	17	31	4	140	115	146	45	*Atu2551*
196	C1_2541934R[*]	-	2541934	2541832	6422	13871	12566	5053	753	909	849	674	*TPP riboswitch (Atu2569, thiC)*
197	C1_2544091R	-	2544091	2543878	10	0	14	7	74	111	141	43	*Atu2573*
198	C1_2557040F	+	2557040	2557179	0	0	35	11	89	103	83	22	*Atu2582*
199	C1_2567189R	-	2567189	2567092	30	23	28	0	93	41	29	43	*Atu2589*
200	C1_2601060R	-	2601060	2600812	0	91	42	18	12	126	20	24	*Atu2617 (cheW)*
201	C1_2618039F	+	2618039	2618177	0	0	0	4	19	0	22	53	*Atu2634*
202	C1_2618930F	+	2618930	2619044	0	0	0	0	0	26	52	18	*Atu2635 (sucB)*
203	C1_2645312F	+	2645312	2645708	0	0	3	7	4	18	58	14	*Atu2663 (typA)*
204	C1_2667196F[*†]	+	2667196	2667281	977	3379	1448	2276	2537	6206	4108	5534	*Intergenic*
205	C1_2670018F	+	2670018	2670091	0	0	0	0	58	26	34	12	*Atu2685 (acnA)*
206	C1_2670880F	+	2670880	2671003	5	51	7	0	105	100	43	37	*Intergenic*
207	C1_2681903F[†]	+	2681903	2681962	0	0	288	128	31	4	13	4	*ffh riboswitch (Atu2697, ffh)*
208	C1_2693188F	+	2693188	2693266	45	79	243	174	50	126	121	286	*Intergenic*
209	C1_2693475R	-	2693475	2693366	5	0	3	0	43	81	52	18	*Atu8028*
210	C1_2707755F	+	2707755	2707951	94	6	10	0	74	52	43	14	*Atu2722 (ompA)*
211	C1_2716245R	-	2716245	2715987	89	51	108	43	411	285	361	122	*5' UTR of Atu2727*
212	C1_2717022R	-	2717022	2716803	0	0	3	4	31	22	58	35	*Atu2728 (chvA)*
213	C1_2741942R	-	2741942	2741761	0	0	7	0	31	7	29	12	*Atu2744*
214	C1_2743541F	+	2743541	2743668	0	0	0	0	27	4	54	20	*Atu2745 (acs)*
215	C1_2767591R	-	2767591	2767377	0	6	3	0	97	59	69	24	*Atu2766 (gdh)*
216	C1_2820698F	+	2820698	2821003	20	0	14	11	50	41	78	35	*Atu2817 (rbsB)*
217	C1_2821322F	+	2821322	2821478	0	0	0	0	43	55	56	8	*Atu2818 (rbsC)*
218	C1_2830437F	+	2830437	2830603	0	17	21	11	140	170	190	47	*Atu2825 (sun)*
219	C1_2834554F	+	2834554	2834648	5	11	24	18	54	55	43	14	*Atu2830 (gidB)*
220	C1_2838704F	+	2838704	2838763	0	0	0	43	31	0	38	45	*Atu2833 (rho)*
221	C1_2839679F	+	2839679	2839879	0	0	3	18	23	41	52	20	*Atu2834*

[*]ncRNAs have been validated with Northern blot analysis and/or 5' and 3' RACE.
[†]ncRNAs have been previously identified or detected by Wilms et al. 2012.
[‡]C1_1533961R corresponds to two sRNAs from Wilms et al. 2012: 1533826-1533764 and 1533957-1533833.
[§]Candidate ncRNAs include a putative Open Reading Frame (ORF).

Table S3-B. List of identified candidate ncRNAs on the linear chromosome. Highly expressed putative ncRNAs were identified when the average depth of coverage of the non-gene-coding region was at least 10 times higher than that in adjacent regions. ncRNA tag was named with the replicon (C1, circular chromosome; C2, linear chromosome; pAt, pAt plasmid; pTi, plasmid Ti) and 5' End position with strand information (F, forward strand; R, reverse strand). RPKM (Reads Per Kilobase per Million mapped reads) in 8 different cDNA libraries were listed along with the information of genes encoded on the opposite strand.

Linear chromosome		Strand	ncRNA position		RPKM (-TEX)				RPKM (+TEX)				
	ncRNA name		5' end	3' end	YEP-L	YEP-S	AB	IND	YEP-L	YEP-S	AB	IND	antisense to
1	C2_2541R	-	2541	2161	30	85	63	36	151	211	175	63	*Intergenic*
2	C2_8435F	+	8435	8665	15	28	38	21	147	152	217	65	*Atu3012*
3	C2_37894R	-	37894	37539	0	6	10	0	35	33	87	18	*Atu3041*
4	C2_56733F	+	56733	56842	10	17	28	14	31	67	29	16	*Atu3056 (crdS)*
5	C2_71955R	-	71955	71851	0	28	24	7	27	37	78	18	*Atu3069*
6	C2_105462R	-	105462	105130	10	11	24	11	43	118	69	35	*Atu3097 (ugpA); Atu3098 (ugpE)*
7	C2_132427R	-	132427	132326	0	6	111	110	27	55	128	207	*Intergenic*
8	C2_132595F	+	132595	132735	679	572	3115	11914	326	362	1770	10380	*Intergenic*
9	C2_133474F[†]	+	133474	133761	169	849	531	224	1470	4945	3129	741	*Atu3127 (rspF)*
10	C2_140230F	+	140230	140473	64	68	83	39	225	192	244	93	*Atu3132*
11	C2_162750F	+	162750	162862	0	11	31	11	19	126	40	55	*5' UTR of Atu3154 (fsr)*
12	C2_175078F	+	175078	175277	0	17	21	7	47	59	52	30	*Atu3165*
13	C2_188494F	+	188494	188687	0	0	0	0	58	4	34	8	*Atu3178*
14	C2_190347F	+	190347	190536	10	11	14	18	62	122	67	41	*Atu3180*
15	C2_204149R[†]	-	204149	204082	174	164	1330	1183	70	15	152	522	*Intergenic*
16	C2_205780R	-	205780	205499	193	147	4215	3587	1602	736	11794	6619	*5' UTR of Atu3196 (speF)*
17	C2_213622F	+	213622	213772	0	0	3	0	12	33	52	16	*Atu3202*
18	C2_232866F[§]	+	232866	233235	253	351	788	408	543	1172	1790	593	*Atu3218*
19	C2_242361F	+	242361	242456	0	23	49	32	19	52	34	41	*Atu3228 (dnaE)*
20	C2_291563F	+	291563	291905	129	323	1125	806	120	163	786	989	*5' UTR of Atu3272*
21	C2_292500R	-	292500	292296	0	0	35	0	62	55	69	35	*Atu3272 (pssN)*
22	C2_312778F[*]	+	312778	312932	337	566	510	281	582	481	636	463	*TPP riboswitch (Atu3286)*
23	C2_346356F	+	346356	346515	10	6	14	0	16	33	52	26	*Atu3314*
24	C2_350061F	+	350061	350268	10	34	31	7	163	281	83	10	*Atu3318*
25	C2_351463R	-	351463	350985	20	23	66	50	85	70	103	43	*Atu3320*
26	C2_352528R	-	352528	352361	45	57	69	32	155	133	150	47	*Atu3321*
27	C2_368090F	+	368090	368221	0	17	10	14	43	70	74	45	*Atu3332*
28	C2_408068F[†]	+	408068	408161	114	232	326	135	105	177	155	112	*Intergenic*
29	C2_408214F	+	408214	408389	104	272	1045	682	279	1401	1075	733	*Intergenic*
30	C2_444544R	-	444544	444346	60	204	167	82	101	107	186	130	*Atu3399*
31	C2_453131R	-	453131	453034	0	23	35	0	81	103	123	45	*Atu3407*
32	C2_465314F	+	465314	465482	0	0	10	11	47	22	36	37	*Atu3417*
33	C2_485721R	-	485721	485439	0	0	10	7	35	67	99	30	*Atu3438*
34	C2_548356F	+	548356	548424	30	0	38	0	97	92	119	69	*Atu3496*
35	C2_552560F	+	552560	552725	0	0	0	0	81	0	16	0	*Atu3501*
36	C2_570047R	-	570047	569977	20	0	21	25	0	4	38	81	*Intergenic*
37	C2_581359F	+	581359	581453	0	0	0	0	54	41	31	10	*Atu3531 (fabG)*
38	C2_596390R	-	596390	596296	0	23	7	0	47	81	22	18	*Atu3546*
39	C2_598167F	+	598167	598339	10	40	56	28	70	159	128	61	*Atu3548*
40	C2_601663F	+	601663	601754	10	23	56	57	19	41	40	67	*Atu3550*
41	C2_603468F	+	603468	603630	64	198	149	75	190	170	69	65	*Intergenic*
42	C2_615336R	-	615336	615055	104	147	135	75	256	262	276	152	*Intergenic*

	Linear chromosome	Strand	ncRNA position		RPKM (-TEX)				RPKM (+TEX)				
	ncRNA name		5' end	3' end	YEP-L	YEP-S	AB	IND	YEP-L	YEP-S	AB	IND	antisense to
43	C2_619696F	+	619696	619837	25	17	10	0	50	44	54	16	*Intergenic*
44	C2_622977R	-	622977	622853	35	141	56	107	147	277	148	124	*Atu3568*
45	C2_630320F	+	630320	630564	0	0	10	0	23	37	85	30	*Atu3576 (xylF)*
46	C2_661206F	+	661206	661351	40	136	59	107	345	776	282	297	*Atu3605*
47	C2_668272F	+	668272	668380	45	40	56	50	113	118	146	77	*Atu3612 (hisC)*
48	C2_684674F	+	684674	684738	55	6	69	71	8	0	20	67	*Intergenic*
49	C2_699418R	-	699418	699029	203	509	497	384	784	1475	1082	747	*Atu3645*
50	C2_710969R	-	710969	710824	0	28	28	7	50	44	63	10	*Atu3662*
51	C2_717107F	+	717107	717228	10	28	49	11	78	111	69	41	*Atu3669*
52	C2_718560F	+	718560	718670	10	6	21	7	62	96	43	28	*Atu3670*
53	C2_734305R	-	734305	734091	5	45	3	0	43	89	38	12	*Atu3675*
54	C2_735151R	-	735151	735047	25	6	0	0	27	48	29	10	*Atu3676*
55	C2_738154R	-	738154	738020	40	23	24	28	81	59	78	43	*Atu3680; Atu3681*
56	C2_759687R	-	759687	759561	0	0	3	21	62	100	38	51	*Atu3685*
57	C2_760607R	-	760607	760435	0	0	28	21	78	92	121	63	*Atu3686*
58	C2_761827R	-	761827	761689	10	0	3	7	109	33	52	18	*Atu3687*
59	C2_767943R	-	767943	767858	40	45	28	4	54	22	69	10	*Atu3692*
60	C2_780641F[†]	+	780641	780701	119	28	427	138	0	0	47	41	*Intergenic*
61	C2_808739F	+	808739	808899	0	0	0	0	39	18	58	32	*Atu3727*
62	Atu8108[†]	-	814020	813852	6387	7329	6712	5632	39686	36186	28844	22878	*6S RNA*
63	C2_824767F[*]	+	824767	824863	595	1432	642	259	5276	6043	2292	914	*Intergenic*
64	C2_825238R[†]	-	825238	825130	84	108	132	89	194	170	208	134	*Atu3742*
65	C2_827995F	+	827995	828097	183	79	142	170	101	52	49	83	*Atu3745*
66	C2_855409R	-	855409	855359	0	0	69	36	0	0	0	0	*Atu3771 (hemH)*
67	C2_868477F	+	868477	868542	0	23	14	11	101	59	52	18	*Atu3784 (gltB)*
68	C2_883060R	-	883060	882858	0	0	0	14	12	22	16	41	*Intergenic*
69	C2_885096F	+	885096	885198	0	0	0	0	12	11	11	20	*Atu3792 (ansP)*
70	C2_914238R[†]	-	914238	914053	0	6	10	7	0	37	60	26	*Atu3817*
71	C2_919257R	-	919257	918774	417	928	351	224	543	1593	607	307	*Atu3822*
72	C2_930384F[†]	+	930384	930457	60	147	576	657	182	96	233	615	*Atu3833*
73	C2_931790F	+	931790	931893	0	0	10	7	54	22	29	20	*Atu3835*
74	C2_938344R	-	938344	938093	5	62	28	0	113	63	43	28	*Atu3843*
75	C2_959190R	-	959190	959103	0	11	42	18	16	26	45	63	*Atu3866*
76	Atu8110	-	979666	979597	179	289	201	178	388	455	161	175	*suhB*
77	C2_985951F	+	985951	986063	0	0	3	0	78	115	114	22	*Atu3890 (glpK)*
78	C2_988978F	+	988978	989107	15	0	24	32	31	44	54	39	*Atu3893*
79	C2_1014267R	-	1014267	1013959	0	0	10	7	47	103	108	45	*Atu3913*
80	C2_1022833R	-	1022833	1022510	79	141	174	99	279	355	428	248	*Atu3920; Atu3921*
81	Atu8080	+	1024262	1024335	5	0	28	25	12	30	43	8	*ctRNA_p42d*
82	C2_1032604F	+	1032604	1032741	0	0	7	4	43	48	49	20	*Atu3930*
83	C2_1041313F	+	1041313	1041468	45	11	0	25	89	26	27	65	*Intergenic*
84	C2_1056686F	+	1056686	1056784	0	0	0	0	50	15	60	24	*Atu3953*
85	C2_1060144F	+	1060144	1060260	0	0	0	0	23	33	25	6	*Atu3956*
86	C2_1060978F[†]	+	1060978	1061205	30	23	42	32	163	144	206	89	*Atu3957*
87	C2_1087738F	+	1087738	1087972	0	0	0	4	23	52	54	12	*Atu3976*
88	C2_1179748F[†]	+	1179748	1179845	20	23	167	92	70	15	150	120	*Atu4061 (exoX)*
89	C2_1181179F	+	1181179	1181321	0	0	0	0	23	18	47	8	*Atu4063*
90	C2_1200650R[†]	-	1200650	1200485	352	311	403	295	314	299	410	181	*Atu4079*
91	C2_1230082R	-	1230082	1229852	0	0	7	0	31	4	52	20	*Atu4110 (sdh)*
92	C2_1261393R	-	1261393	1261273	20	357	1354	366	12	152	383	130	*Atu4144*

	Linear chromosome	Strand	ncRNA position		RPKM (-TEX)				RPKM (+TEX)				
	ncRNA name		5' end	3' end	YEP-L	YEP-S	AB	IND	YEP-L	YEP-S	AB	IND	antisense to
93	C2_1268008F	+	1268008	1268085	476	504	392	114	70	78	69	43	*Intergenic*
94	C2_1281891R†	-	1281891	1281788	10	453	431	281	101	1867	1658	666	*Intergenic*
95	C2_1302178F	+	1302178	1302353	10	68	38	18	43	43	49	28	*Atu4178*
96	C2_1303985F*	+	1303985	1304143	1597	430	1382	1800	7403	1648	4913	3853	*Atu4179*
97	C2_1305779F*	+	1305779	1305881	1726	543	2406	3228	5564	580	3553	5343	*Intergenic*
98	C2_1306368F	+	1306368	1306506	248	45	240	394	361	33	314	457	*Intergenic*
99	C2_1310192F	+	1310192	1310248	183	51	274	320	50	85	87	85	*3' UTR of Atu4186*
100	C2_1314323R	-	1314323	1314010	15	34	42	4	97	81	96	39	*Atu4192*
101	C2_1320369R	-	1320369	1320030	25	45	42	25	81	133	132	45	*Atu4196*
102	C2_1324083F*	+	1324083	1324449	69	108	101	43	178	144	206	91	*Intergenic*
103	C2_1327187F	+	1327187	1327351	25	57	35	0	93	152	85	22	*Intergenic*
104	C2_1327583F	+	1327583	1327677	5	6	3	11	54	37	69	41	*Atu4205 (tetR)*
105	C2_1368664R	-	1368664	1368573	0	0	24	0	43	26	34	32	*Atu4244*
106	C2_1369454R	-	1369454	1369355	0	0	0	0	27	63	22	0	*Atu4244; Atu4245*
107	C2_1383778F	+	1383778	1383948	55	11	66	46	54	33	54	53	*Atu4258*
108	C2_1386054F	+	1386054	1386105	0	0	0	21	0	0	34	26	*Atu4259*
109	C2_1386463F	+	1386463	1386602	79	91	52	28	105	115	38	22	*Intergenic*
110	C2_1410638F	+	1410638	1410734	0	0	0	0	54	0	4	2	*Atu4286*
111	C2_1418097R	-	1418097	1418040	20	28	69	36	81	52	74	51	*Intergenic*
112	C2_1446873R	-	1446873	1446634	0	0	17	21	19	67	67	41	*Atu4218*
113	C2_1456982R§	-	1456982	1456608	55	628	653	327	70	488	638	408	*Atu4327*
114	C2_1458121R	-	1458121	1458029	25	0	66	43	43	44	141	55	*Atu4329 (bexD)*
115	C2_1483623R	-	1483623	1483470	15	28	52	60	182	103	323	140	*Atu4348 (vgrG)*
116	C2_1485175R	-	1485175	1485081	0	0	0	0	4	18	47	8	*Atu4350*
117	C2_1492506R†	-	1492506	1492426	129	294	2045	1186	35	148	56	45	*Intergenic*
118	C2_1495977F	+	1495977	1496045	0	0	21	0	27	92	52	43	*Atu4359*
119	C2_1514611R	-	1514611	1514502	0	0	0	0	0	48	7	22	*Intergenic*
120	C2_1523357F	+	1523357	1523728	0	0	3	0	23	48	49	16	*Atu4386 (norD)*
121	C2_1525954F	+	1525954	1526054	0	0	3	0	50	26	45	18	*Atu4388 (norB)*
122	C2_1550733R	-	1550733	1550560	0	0	0	0	35	52	60	14	*Atu4412 (rzcA)*
123	C2_1572384R	-	1572384	1572295	0	0	0	0	12	33	60	20	*Atu4429*
124	C2_1587278R	-	1587278	1587149	0	28	31	50	0	0	0	12	*Atu4442*
125	C2_1619291F	+	1619291	1619430	0	17	21	11	78	70	38	45	*Atu4475*
126	C2_1623126R	-	1623126	1622953	15	23	42	43	163	207	152	156	*Atu4478 (emrA)*
127	C2_1623406R	-	1623406	1623345	0	17	24	0	54	11	45	16	*Atu4479 (emrB)*
128	C2_1630159F	+	1630159	1630428	144	277	63	32	1366	1656	197	254	*Atu4484*
129	C2_1633031F	+	1633031	1633243	55	91	205	323	357	495	710	875	*5' UTR of Atu4487*
130	C2_1636311R†	-	1636311	1636109	0	23	10	25	50	118	27	67	*Atu4490*
131	C2_1641838R	-	1641838	1641566	0	0	21	0	16	37	74	26	*Atu4494 (kdgA)*
132	C2_1730420F	+	1730420	1730554	30	51	80	53	241	262	159	83	*Atu4581*
133	C2_1747764F	+	1747764	1747960	0	28	59	64	47	85	49	61	*Intergenic*
134	C2_1751242F	+	1751242	1751325	20	51	198	398	62	74	148	154	*Atu4606*
135	C2_1753644F	+	1753644	1753983	45	96	122	110	31	137	155	100	*Atu4607 (nodX)*
136	C2_1758425F†	+	1758425	1758861	50	62	108	71	113	166	215	114	*Atu4611 (tnp); Atu4613*
137	C2_1763538F	+	1763538	1763873	0	0	10	11	58	41	52	20	*Atu4616 (rfbD)*
138	C2_1764441F	+	1764441	1764621	30	79	118	50	198	244	244	97	*Atu4617 (rffB)*
139	C2_1765302F	+	1765302	1765398	0	17	94	160	43	67	63	134	*Atu4618 (rfbC)*
140	C2_1765802F	+	1765802	1766034	0	34	31	4	54	70	90	39	*Atu4619 (uvrD)*
141	C2_1783758R	-	1783758	1783557	124	96	351	192	400	307	461	179	*Atu4632*
142	C2_1787601F	+	1787601	1787791	5	11	28	11	105	78	114	43	*Atu4636*

	Linear chromosome	Strand	ncRNA position		RPKM (-TEX)				RPKM (+TEX)				
	ncRNA name		5' end	3' end	YEP-L	YEP-S	AB	IND	YEP-L	YEP-S	AB	IND	antisense to
143	C2_1791414R[†]	-	1791414	1791186	10	45	52	18	47	59	58	45	*Atu4639*
144	C2_1804982F	+	1804982	1805039	0	0	17	0	0	0	18	28	*Atu4651 (argH)*
145	C2_1813420R	-	1813420	1813318	0	0	38	18	31	22	74	39	*Atu4657*
146	C2_1823501R	-	1823501	1823343	0	0	17	0	27	37	47	16	*Atu4664; Atu4665 (melA)*
147	C2_1830949F[†§]	+	1830949	1831215	4007	1449	32097	36026	6111	1848	17836	16227	*Atu4670*
148	C2_1831446F[*†]	+	1831446	1831607	689	272	3458	3970	10541	2865	25085	25357	*Intergenic*
149	C2_1847140R	-	1847140	1846793	25	51	59	57	85	115	166	97	*Atu4688*
150	C2_1849909R	-	1849909	1849688	5	23	52	28	43	52	56	47	*Atu4691*
151	C2_1871050R	-	1871050	1870903	30	45	7	4	47	81	63	14	*Atu4708 (fdhF)*
152	C2_1891119F[†]	+	1891119	1891229	317	532	1500	671	89	177	161	270	*Intergenic*
153	C2_1896022F	+	1896022	1896105	0	0	21	0	0	4	25	41	*Atu4731*
154	C2_1902921F	+	1902921	1903001	431	362	170	156	1191	835	309	169	*Intergenic*
155	C2_1911457F	+	1911457	1911660	0	6	3	18	23	22	20	51	*Atu4742*
156	C2_1929391F	+	1929391	1929590	0	11	28	4	35	85	76	28	*Atu4760*
157	C2_1952079F	+	1952079	1952343	0	0	3	0	31	7	45	16	*Atu4781*
158	C2_1966629F[*†]	+	1966629	1966687	0	102	479	163	27	0	25	8	*Intergenic*
159	C2_1972126F	+	1972126	1972388	10	11	21	4	35	18	43	26	*Atu4796*
160	C2_1982652R	-	1982652	1982418	10	11	90	57	182	211	282	116	*Atu4805*
161	C2_2019043R	-	2019043	2018972	25	6	28	28	81	55	105	47	*Atu4838*
162	C2_2038173F	+	2038173	2038349	0	17	14	11	35	100	83	16	*Atu4855 (traA)*
163	C2_2058235R	-	2058235	2058094	149	181	59	32	481	403	166	69	*Atu4876*
164	C2_2066496R	-	2066496	2066340	25	45	104	92	35	63	94	61	*Atu4889*

[*]ncRNAs have been validated with Northern blot analysis and/or 5' and 3' RACE.

[†]ncRNAs have been previously identified or detected by Wilms et al. 2012.

[§]Candidate ncRNAs include a putative Open Reading Frame (ORF).

Table S3-C. List of identified candidate ncRNAs on the pAt plasmid. Highly expressed putative ncRNAs were identified when the average depth of coverage of the non-gene-coding region was at least 10 times higher than that in adjacent regions. ncRNA tag was named with the replicon (C1, circular chromosome; C2, linear chromosome; pAt, pAt plasmid; pTi, plasmid Ti) and 5' End position with strand information (F, forward strand; R, reverse strand). RPKM (Reads Per Kilobase per Million mapped reads) in 8 different cDNA libraries were listed along with the information of genes encoded on the opposite strand.

pAt plasmid		Strand	ncRNA position		RPKM (-TEX)				RPKM (+TEX)				
	ncRNA name		5' end	3' end	YEP-L	YEP-S	AB	IND	YEP-L	YEP-S	AB	IND	antisense to
1	pAt_14036R	-	14036	13925	0	0	0	18	12	11	45	45	*Intergenic*
2	pAt_30777R	-	30777	30285	89	232	222	92	252	314	296	165	*Atu5029*
3	pAt_34379F	+	34379	34753	5	17	21	14	19	15	47	22	*Atu5033*
4	pAt_35484R	-	35484	35365	30	0	35	25	70	44	22	18	*Atu5034*
5	pAt_37435R	-	37435	37133	35	96	94	64	140	322	293	142	*Atu5037*
6	pAt_43691F†	+	43691	43880	69	170	247	82	419	410	477	193	*Atu5042*
7	pAt_45638F	+	45638	45894	15	34	35	21	50	133	150	43	*Atu5045*
8	pAt_49839R	-	49839	49756	0	6	21	0	35	41	54	14	*Atu5050*
9	pAt_55271R*†	-	55271	55154	45	866	3597	877	105	717	3728	1042	*Intergenic*
10	pAt_66465F	+	66465	66610	25	6	31	28	101	96	67	39	*Atu5068*
11	pAt_71130F	+	71130	71298	35	74	90	50	194	181	190	122	*Atu5071 (dppA)*
12	pAt_85273R	-	85273	84906	144	11	0	11	473	118	25	26	*Atu5083*
13	pAt_91346R	-	91346	91174	10	45	63	39	171	174	175	51	*Intergenic*
14	pAt_92195R	-	92195	92029	64	45	52	11	70	44	54	37	*Atu5088; Atu5089*
15	pAt_95607F	+	95607	95700	5	0	0	0	124	63	49	14	*Atu5091 (rcdB)*
16	pAt_98054F†	+	98054	98246	64	113	132	85	241	85	170	136	*5' UTR of Atu5094 (tnpA)*
17	pAt_111447R§	-	111447	111052	25	57	66	21	279	455	309	87	*Atu5104*
18	pAt_124194F†	+	124194	124309	40	51	66	28	89	89	110	41	*Atu5117*
19	pAt_130045F	+	130045	130243	0	6	17	0	101	85	85	30	*Atu5123 (atrC)*
20	pAt_142309R	-	142309	142169	347	1098	389	192	47	214	85	89	*Intergenic*
21	pAt_157836F*†	+	157836	158083	288	656	622	259	966	1279	1243	506	*Atu5157 (atsD); Atu5158*
22	pAt_161710R	-	161710	161585	0	0	0	0	12	4	25	32	*Atu5164 (avhB3)*
23	pAt_162266R	-	162266	162076	0	23	10	43	35	81	60	114	*Atu5165 (avhB4)*
24	pAt_177141R	-	177141	176948	20	40	28	7	43	126	54	18	*Atu5178*
25	pAt_183064F	+	183064	183229	5	11	0	7	78	74	47	26	*Atu5187*
26	pAt_185242R	-	185242	185114	0	0	0	0	8	22	45	12	*Atu5189*
27	pAt_187869F	+	187869	187966	0	0	0	0	116	74	69	28	*Atu5192*
28	pAt_414717R	-	414717	414510	74	136	115	50	392	292	334	122	*Atu5419*
29	pAt_424531F	+	424531	424701	25	17	31	4	101	70	63	28	*Intergenic*
30	pAt_434701R	-	434701	434536	0	6	10	7	16	18	58	18	*Atu5442*
31	pAt_437832R	-	437832	437658	20	34	52	28	206	229	204	106	*Atu5444*
32	pAt_441463R	-	441463	441377	30	0	66	0	12	22	31	47	*Intergenic*
33	pAt_444075R	-	444075	443752	25	45	59	43	81	129	157	69	*Atu5452 (attE)*
34	pAt_451816R	-	451816	451650	0	0	3	25	50	37	67	45	*Atu5459*
35	pAt_464614R	-	464614	464557	0	23	49	25	23	0	25	45	*Intergenic*
36	pAt_481533F	+	481533	481612	0	0	69	39	8	11	67	167	*Atu5491 (catE)*
37	pAt_497789R	-	497789	497683	0	17	21	36	8	92	47	104	*Atu5506*
38	pAt_500617R	-	500617	500541	0	0	69	298	136	44	49	345	*Atu5508 (gst)*
39	pAt_505815F	+	505815	505873	0	0	52	18	0	0	0	0	*Intergenic*
40	pAt_506281R	-	506281	506172	10	23	69	57	159	81	87	35	*5' UTR of Atu5513*
41	pAt_514334F	+	514334	514452	30	0	52	82	105	44	90	73	*Atu5522*
42	pAt_521235F	+	521235	521381	10	0	7	7	47	30	67	30	*Atu5528*
43	pAt_534208F	+	534208	534389	60	40	69	39	167	144	197	93	*Atu5539*

*ncRNAs have been validated with Northern blot analysis and/or 5' and 3' RACE.

†ncRNAs have been previously identified or detected by Wilms et al. 2012.

§Candidate ncRNAs include a putative Open Reading Frame (ORF).

Table S3-D. List of identified candidate ncRNAs on the Ti plasmid. Highly expressed putative ncRNAs were identified when the average depth of coverage of the non-gene-coding region was at least 10 times higher than that in adjacent regions. ncRNA tag was named with the replicon (C1, circular chromosome; C2, linear chromosome; pAt, pAt plasmid; pTi, plasmid Ti) and 5' End position with strand information (F, forward strand; R, reverse strand). RPKM (Reads Per Kilobase per Million mapped reads) in 8 different cDNA libraries were listed along with the information of genes encoded on the opposite strand.

Ti plasmid		Strand	ncRNA position		RPKM (-TEX)				RPKM (+TEX)				
	ncRNA name		5' end	3' end	YEP-L	YEP-S	AB	IND	YEP-L	YEP-S	AB	IND	antisense to
1	pTi_5440F[*]	+	5440	5738	99	255	576	692	163	303	410	573	*Intergenic*
2	pTi_5973R	-	5973	5766	10	11	21	50	97	115	72	89	*Intergenic*
3	pTi_17598R	-	17598	17400	0	0	7	32	85	85	87	83	*Atu6011 (tms1)*
4	pTi_23992F[†]	+	23992	25082	129	170	240	291	136	181	159	236	*Atu6015 (nos); Atu6016 (ocd)*
5	pTi_25540F	+	25540	25661	0	0	10	14	50	70	56	51	*Atu6016 (ocd)*
6	pTi_45456F	+	45456	45542	0	0	0	28	97	118	81	37	*Atu6035 (trbL)*
7	pTi_51539F	+	51539	51705	0	0	3	4	62	78	60	47	*Atu6042 (traI)*
8	pTi_54770R (rpeE)[†]	-	54770	54130	79	51	101	231	372	233	332	424	*Atu6044 (repB)*
9	pTi_65619R	-	65619	65425	64	141	233	295	547	643	571	626	*Atu6053*
10	pTi_79525F	+	79525	79908	10	34	45	78	43	52	45	73	*Atu6067*
11	pTi_82838F[*]	+	82838	83218	10	6	7	50	66	26	56	124	*Atu6069 (rbsA); Atu6070 (rbsB)*
12	pTi_83907F	+	83907	84012	0	0	0	4	4	0	9	41	*Atu6070 (rbsB)*
13	pTi_84241F	+	84241	84602	104	79	142	263	163	122	175	260	*Atu6071 (aiiB)*
14	pTi_87172F	+	87172	87423	50	79	42	60	268	222	206	152	*Atu6073 (dfpA)*
15	pTi_91643F[†]	+	91643	91798	15	62	31	67	78	85	121	126	*Atu6076 (dfpB)*
16	pTi_97611R	-	97611	97376	15	0	17	57	97	89	94	181	*Atu6081*
17	pTi_99872R	-	99872	99690	0	0	17	7	35	52	81	77	*Atu6083*
18	pTi_104793R	-	104793	104686	0	0	7	43	39	85	54	63	*Atu6086 (nrtB)*
19	pTi_105783R	-	105783	105658	0	0	0	0	19	0	36	20	*Atu6087 (nrtC); Atu6088*
20	pTi_106595R	-	106595	106271	5	6	21	43	27	11	49	81	*Atu6088*
21	pTi_107903R	-	107903	107560	15	40	45	60	39	59	52	102	*Atu6089*
22	pTi_116265R	-	116265	116124	10	23	24	25	35	63	25	47	*Intergenic*
23	pTi_125132R[*†]	-	125132	124766	74	136	156	210	330	436	381	364	*Atu6101*
24	pTi_148374R[†]	-	148374	148279	412	260	1677	2852	691	370	1183	3349	*Atu6129 (traB)*
25	pTi_149938F[†]	+	149938	150154	3298	4465	2545	3232	15992	17365	6638	9129	*5' UTR of Atu6131*
26	pTi_160741R	-	160741	160330	20	17	49	67	78	96	103	140	*Atu6140 (accb)*
27	pTi_166009R	-	166009	165829	10	17	31	64	66	103	90	85	*Atu6147*
28	pTi_167705R	-	167705	167504	10	45	17	18	74	81	43	43	*Atu6149*
29	pTi_181454R	-	181454	181227	30	40	76	96	105	85	146	87	*Atu6166 (virA)*
30	pTi_182044R	-	182044	181899	25	40	76	96	81	155	92	116	*Atu6166 (virA)*
31	pTi_182957R	-	182957	182884	0	57	108	320	0	11	20	126	*Atu6166 (virA)*
32	pTi_187104R	-	187104	187020	0	0	0	0	0	59	49	20	*Atu6170 (virB4)*
33	pTi_189018R	-	189018	188898	0	23	28	32	39	44	99	89	*Atu6172 (virB6)*
34	pTi_189991R	-	189991	189796	5	23	45	82	35	18	65	144	*Atu6174 (virB8)*
35	pTi_190683R[*†]	-	190683	190309	89	141	201	252	407	351	553	530	*Atu6175 (virB9)*
36	pTi_191667R	-	191667	191470	7	8	1	17	44	28	39	35	*Atu6176 (virB10)*
37	pTi_196737R	-	196737	196549	25	11	21	39	178	140	83	53	*Atu6182 (virD2)*
38	pTi_198827R	-	198827	198585	15	28	73	50	109	248	276	108	*Atu6183 (virD3)*
39	pTi_200229R[†]	-	200229	200107	114	147	250	387	182	329	164	201	*Atu6184 (virD4)*
40	pTi_201463R[*†]	-	201463	201118	134	306	399	469	275	307	475	648	*Atu6184 (virD4)*
41	pTi_203582R	-	203582	203369	0	0	0	4	43	22	29	12	*Atu6185 (virD5)*
42	pTi_204428R	-	204428	204118	5	17	24	32	35	37	54	61	*Atu6185 (virD5)*

Ti plasmid		Strand	ncRNA position		RPKM (-TEX)				RPKM (+TEX)				
	ncRNA name		5' end	3' end	YEP-L	YEP-S	AB	IND	YEP-L	YEP-S	AB	IND	antisense to
43	pTi_205576R	-	205576	205339	0	79	42	85	85	225	92	142	*Intergenic*
44	pTi_206209R	-	206209	206143	0	0	14	11	78	18	47	51	*Atu6189 (virE1)*
45	pTi_206590R[†]	-	206590	206424	35	147	198	135	357	469	329	227	*Atu6190 (virE2)*
46	pTi_207119R	-	207119	206724	5	17	42	32	89	96	125	69	*Atu6190 (virE2)*
47	pTi_208901R	-	208901	208631	114	113	111	121	132	133	130	138	*Atu6191 (virE3)*

[*]ncRNAs have been validated with Northern blot analysis and/or 5' and 3' RACE.

[†]ncRNAs have been previously identified or detected by Wilms et al. 2012.

Table S4-A. Candidate ncRNAs up-regulated by AS. Differentially expressed ncRNAs were identified by DESeq analysis ($P < 0.05$). ncRNA position, abundance (RPKM, Reads Per Kilobase per Million mapped reads), fold change and P-value were provided. Putative vir box sequences and the relative location to the 5' end of each ncRNA were given in the last two columns.

		ncRNA position			RPKM (-TEX)				RPKM (+TEX)							putative vir box sequences	
	ncRNA ID	5' end	3' end	Strand	YEP-L	YEP-S	AB	IND	YEP-L	YEP-S	AB	IND	Fold change	*P-value*	antisense to	box 1	box 2
**Circular choromosome**																	
1	C1_110258F	110258	110380	+	5420	5444	2955	3636	559	381	220	983	2.00	0.019	Intergenic	-164: TTCTTTTGAAAG	-89: TTCGGATGAAAA
2	C1_112676R	112676	112535	-	541	402	819	8686	5028	3404	3819	23172	9.06	0.000	Intergenic	-40: TTCCCTTGAAAA	
3	C1_813546R	813546	813485	-	630	1336	1118	2013	70	67	18	114	2.65	0.010	Intergenic	-17: TTCAGATGTCGC	
4	C1_1003889R	1003889	1003434	-	0	11	87	277	12	7	110	357	4.30	0.004	*Atu1007*	-197: AAGCATTGAAAA	-57: ACCATATGAACC
5	C1_1052093R	1052093	1051804	-	600	787	149	366	644	1294	211	443	3.02	0.028	Intergenic	-132: GGCATTTAAAAC	
6	C1_1464632R	1464632	1464500	-	84	255	309	1392	54	336	152	1202	7.47	0.000	Intergenic	-120: CAAAAATGATAT	
7	C1_1705208F	1705208	1705458	+	0	23	14	131	66	92	29	236	11.35	0.000	Intergenic	-123: GGCAAATGACAA	-77: TCCATTTTTTAC
8	C1_2032975F	2032975	2033106	+	144	28	1104	3619	78	15	318	2193	5.48	0.000	*Atu2071*	-126: CGTCATTGAAGC	
9	C1_2139202F	2139202	2139332	+	818	1387	1597	7564	644	902	2166	21501	9.84	0.000	Intergenic	-86: ATCACTAAAAAT	-72: TGTTTTTGTAAT
10	C1_2290013R	2290013	2289373	-	620	1013	28	67	248	333	2	30	4.35	0.004	*Atu2311 (xdhC)*	-102: TTCAGAGGGAAC	
**Linear choromosome**																	
1	C2_132595F	132595	132735	+	679	572	3115	11914	326	362	1770	10380	6.09	0.000	Intergenic	-141: CGCAGGTGAAGT	-14: CGCAGGTGAAGT
**pAt plasmid**																	
1	pAt_500617R	500617	500541	-	0	0	69	298	136	44	49	345	7.10	0.008	*Atu5508 (gst)*		
**Ti plasmid**																	
1	pTi_82838F	82838	83218	+	10	6	7	50	66	26	56	124	3.69	0.016	*Atu6069 (rbsA); A*	-163: TGGCCTCGTAAT	
2	pTi_148374R	148374	148279	-	412	260	1677	2852	691	370	1183	3349	2.86	0.023	*Atu6129 (traB)*	-106: CACCGTTGCGAT	-79: GTCAAGCGAACC
3	pTi_182957R	182957	182884	-	0	57	108	320	0	11	20	126	4.75	0.030	*Atu6166 (virA)*	-145: TCCAATTGCAAA	

Table S4-B. Candidate ncRNAs down-regulated by AS. Differentially expressed ncRNAs were identified by DESeq analysis (*P* < 0.05). ncRNA position, abundance (RPKM, Reads Per Kilobase per Million mapped reads), fold change and P-value were provided.

		ncRNA position		Strand	RPKM (-TEX)				RPKM (+TEX)				Fold change	*P-value*	antisense to
	ncRNA ID	5' end	3' end		YEP-L	YEP-S	AB	IND	YEP-L	YEP-S	AB	IND			
**Circular choromosome**															
1	C1_247797F	247797	247930	+	0	11	52	0	124	137	168	45	0.25	0.035	*Atu0249*
2	C1_883375F	883375	883594	+	40	23	63	4	194	92	94	16	0.15	0.032	Intergenic
3	C1_1626061F	1626061	1626474	+	55	119	1247	849	12	30	509	43	0.74	0.033	*Atu1638; Atu1639*
4	C1_2087200F	2087200	2087384	+	1661	8580	6413	1243	6545	20617	11237	2333	0.27	0.004	Intergenic
5	C1_2437621F	2437621	2437761	+	1572	402	1319	92	1036	336	1660	142	0.10	0.000	*Intergenic*
**Linear choromosome**															
1	C2_37894R	37894	37539	-	0	6	10	0	35	33	87	18	0.23	0.029	*Atu3041*
**pAt plasmid**															
1	pAt_55271R	55271	55154	-	45	866	3597	877	105	717	3728	1042	0.35	0.025	*Intergenic*

Table S4-C. Candidate ncRNAs accumulated during stationary phase. Differentially expressed ncRNAs were identified by DESeq analysis ($P < 0.05$). ncRNA position, abundance (RPKM, Reads Per Kilobase per Million mapped reads), fold change and P-value were provided.

		ncRNA position		Strand	RPKM (-TEX)				RPKM (+TEX)				Fold change	*P-value*	antisense to
	ncRNA ID	5' end	3' end		YEP-L	YEP-S	AB	IND	YEP-L	YEP-S	AB	IND			
**Circular choromosome**															
1	C1_293634F	293634	293992	+	15	119	14	18	50	251	20	24	6.02	0.0220	*Atu0301 (dnaN)*
2	C1_813854F	813854	814010	+	60	328	201	167	74	429	379	331	5.99	0.0311	Intergenic
3	C1_942251R	942251	942016	-	233	1171	830	1080	636	3179	1046	1109	5.27	0.0137	*Intergenic*
4	C1_998095R	998095	998034	-	0	113	497	277	0	30	29	89	Inf	0.0391	Intergenic
5	C1_1275443R	1275443	1275297	-	2713	12965	25899	11016	8574	36917	30473	16440	4.66	0.0166	*Atu1287*
6	C1_1589675R	1589675	1589365	-	10	108	52	21	39	140	76	28	5.57	0.0425	Atu1607 (radC)
7	C1_1711559R	1711559	1711329	-	79	928	840	497	23	159	179	248	11.57	0.0038	*Intergenic*
8	C1_2087200F	2087200	2087384	+	1661	8580	6413	1243	6545	20617	11237	2333	3.78	0.0270	Intergenic
9	C1_2208947F	2208947	2209298	+	5	40	73	60	31	159	233	93	5.85	0.0451	*Atu2238 (fbcB)*
10	C1_2601060R	2601060	2600812	-	0	91	42	18	12	126	20	24	20.40	0.0034	Atu2617 (cheW)
**Linear choromosome**															
1	C2_133474F	133474	133761	+	169	849	531	224	1470	4945	3129	741	3.71	0.0269	*Atu3127 (rspF)*
2	C2_1261393R	1261393	1261273	-	20	357	1354	366	12	152	383	130	17.32	0.0079	Atu4144
3	C2_1281891R	1281891	1281788	-	10	453	431	281	101	1867	1658	666	22.25	0.0001	*Intergenic*
4	C2_1456982R	1456982	1456608	-	55	628	653	327	70	488	638	408	9.69	0.0024	Intergenic
**pAt plasmid**															
1	pAt_55271R	55271	55154	-	45	866	3597	877	105	717	3728	1042	11.59	0.0027	*Intergenic*
**Ti plasmid**															
1	pTi_205576R	205576	205339	-	0	79	42	85	85	225	92	142	3.84	0.0201	*Intergenic*

Table S4-D. Candidate ncRNAs suppressed during stationary phase. Differentially expressed ncRNAs were identified by DESeq analysis (*P* < 0.05). ncRNA position, abundance (RPKM, Reads Per Kilobase per Million mapped reads), fold change and P-value were provided.

		ncRNA position			RPKM (-TEX)				RPKM (+TEX)						
	ncRNA ID	5' end	3' end	Strand	YEP-L	YEP-S	AB	IND	YEP-L	YEP-S	AB	IND	Fold change	*P-value*	antisense to
**Circular choromosome**															
1	C1_109477F	109477	109594	+	1503	413	15354	24677	16287	3009	37950	34389	0.20	0.0318	*Intergenic*
2	C1_982030R	982030	981727	-	337	57	3986	5579	962	229	5155	4584	0.23	0.0300	Atu0986
3	C1_1667597F	1667597	1667843	+	164	17	0	0	493	59	16	8	0.12	0.0100	*Atu1680 (ppiB)*
4	C1_2163256F	2163256	2163370	+	5043	1273	28677	25589	17172	4036	27964	27289	0.25	0.0399	suhB (=AbcR1)
**Linear choromosome**															
1	C2_1303985F	1303985	1304143	+	1597	430	1382	1800	7403	1648	4913	3853	0.24	0.0431	*Atu4179*
2	C2_1305779F	1305779	1305881	+	1726	543	2406	3228	5564	580	3553	5343	0.17	0.0206	Intergenic
3	C2_1306368F	1306368	1306506	+	248	45	240	394	361	33	314	457	0.14	0.0325	*Intergenic*
**pAt plasmid**															
1	pAt_85273R	85273	84906	-	144	11	0	11	473	118	25	26	0.22	0.0168	*Atu5083*

Table S5. Selected ncRNAs for 5' and 3' RACE. Twenty ncRNAs have been selected for validation with 5' and 3' RACE from all four replicons. Three ncRNAs were not amplified by PCR. Predicted 5' and 3' ends by RNA sequencing were provided with the 5' and 3' ends validated with RACE. Genes encoded on the complementary strand were given on the right-most column.

		RNA seq results			RACE results			
	**ncRNA tag**	**5' end**	**3' end**	**Size (nt)**	**5' end**	**3' end**	**Size (nt)**	**antisense to**
**Circular chromosome**								
1	C1_110258F*	110258	110380	123	110161	110346	186	Intergenic
2	C1_813550R†	813546	813485	62	813555	813535	21	Intergenic
3	C1_1643533F	1643533	1643599	67	1643485	1643683	199	Intergenic
4	C1_2273315F†	2273315	2273376	62	2273241	2273368	128	Intergenic
5	C1_2667196F*†	2667196	2667281	86	2667196	2667280	85	Intergenic
**Linear chromosome**								
1	C2_1324083F	1324083	1324449	367	1323780	1324393	614	Atu4200
2	C2_1966629F†	1966629	1966687	59	1966571	1966694	124	Intergenic
**At plasmid**								
1	pAT_55271R*†	55271	55154	118	55154	55277	124	Intergenic
2	pAT_157836F*†	157836	158083	248	157840	158108	269	*Atu5157* (*atsD*); *Atu5158*
**Ti plasmid**								
1	pTi_5440F	5440	5738	299	5439	5747	309	Intergenic
2	pTi_54770R† (repE)	54770	54130	641		54062		Atu6044 (repB)
3	pTi_82838F	82838	83218	621	82933	83163	231	Atu6069 (rbsA); Atu6070 (rbsB)
4	pTi_84241F	84241	84602	362	84210	84492	283	Atu6071 (aiiB)
5	pTi_125132R†	125132	124766	367	125132	124994	139	Atu6101
6	pTi_148374R†	148374	148279	96	n/a	n/a		Atu6129 (traB)
7	pTi_190683R†	190683	190309	375	190702	190311	392	Atu6175 (virB9)
8	pTi_201463R†	201463	201118	346	201398	201234	165	Atu6184 (virD4)
9	pTi_206590R†	206590	206424	167	n/a	n/a		Atu6190 (virE2)

*ncRNAs have been validated with Northern blot analysis.
†ncRNAs have been previously identified or detected by Wilms et al. 2012.
n/a, no amplication

Table S6. Oligonucleotides used in this study.

	Oligonucleotide name	Purpose	Sequence
1	C1_101545R-Hyb-1	Northern blot probe	CTGGCTCGTTAGGGCTGCTTCCTTC
2	C1_109477F-Hyb-1	Northern blot probe	GACAATTGGGAGGAGGAGTATTGTCAGTCC
3	C1_109596F-Hyb-1	Northern blot probe	ACTTGGGTCGAATTTGAAGGAAAAGATCGT
4	C1_110258F-Hyb-1	Northern blot probe	ATCCGTTTTTCGTCAGTGCCAGTACAT
5	C1_112676R-Hyb-1	Northern blot probe	AGCTATCAGACTACGGCGTGATCAGG
6	C1_445306F-Hyb-1	Northern blot probe	ATGATATAGCTGTCGCACTGGCCTTGAATC
7	C1_942251R-Hyb-1	Northern blot probe	GGTCATAAACGGTCTGGGGGATTGTC
8	C1_982030R-Hyb-1	Northern blot probe	AAACGATGGTAACCCTGACTGGAGCGAG
9	C1_1275443R-Hyb-1	Northern blot probe	TTGTTTTTCAGGTTTCGCGTGATCTGTAAC
10	C1_1345805R-Hyb-1	Northern blot probe	AGGAAACACCTGCGAGCCCTCAAGT
11	C1_1745471R-Hyb-1	Northern blot probe	GAGAGAAGCTTATGGGCTGCCAAAAATGTA
12	C1_2087200F-Hyb-1	Northern blot probe	ATCTACGGTTCTTAACTGCGTTTGGTCTTC
13	C1_2139202F-Hyb-1	Northern blot probe	AAGATGCATTCCGGCGGATCAAAAT
14	C1_2163255F-Hyb-1	Northern blot probe	CCTTTAAGTGTGAAGGTCAAAAACCTCCAG
15	C1_2446240R-Hyb-1	Northern blot probe	AACACCATCGAAAATATTCTGCAAATCGGT
16	C1_2667196F-Hyb-1	Northern blot probe	CTGGGGGTAAAGGGTCAATGCAC
17	C2_312778F-Hyb-1	Northern blot probe	GATCAGGTTCAAGGGTCCGGTTCAC
18	C2_824767F-Hyb-1	Northern blot probe	GCTCTCAAGGTAAAACAGGGAGAAAAATCA
19	C2_1303985F-Hyb-1	Northern blot probe	ACAACAAGCATTCAGCTCATCAACCTGATT
20	C2_1305779F-Hyb-1	Northern blot probe	TCATCCTGTTCTAGACAGGCGTCTTACTGT
21	C2_1831446F-Hyb-1	Northern blot probe	CAGGCTTTCTGAAAAGATGAACAGCGATAT
22	pAt55271R-Hyb-1	Northern blot probe	GCGATGAGAAACCGGAAAAACCATC
23	pAt157836F-Hyb-1	Northern blot probe	GTATGTAAGATTGAAGCGTCTGACAATCACCGTTT
24	C1_2541934R-Hyb-1	Northern blot probe	TACGCCAGTATGAACTGGATCAGGTTCAAC
25	Atu2569-Hyb-1	Northern blot probe	GTCTTCAGCTGTTTGTCCATGTTTTCCTTG
26	A3	5' RACE: 5' RNA adapter	AUAUGCGCGAAUUCCUGUAGAACGAACACUAGAAGAAA
27	A3SP-F1	5' RACE: A3 specific primer for first round PCR	GCGCGAATTCCTGTAGA
28	A3SP-F3	5' RACE: A3 specific primer for second round PCR	CGAATTCCTGTAGAACGAACA
29	C1_813550R-R1	5' RACE: reverse primer for first round PCR	GGTCACCCGAACGAAAGAG
30	C1_813550R-R2	5' RACE: reverse primer for second round PCR	CGACGGGCCTCTTTCGTTC
31	C1_110258F-R1	5' RACE: reverse primer for first round PCR	AAAGCGTTCAACGGTCTGAT

	Oligonucleotide name	Purpose	Sequence
32	C1_110258F-R2	5' RACE: reverse primer for second round PCR	TTCAACGGTCTGATGTTCCA
33	C1_1643533F-R1	5' RACE: reverse primer for first round PCR	AAGACCAGCCTTTTCGGAAG
34	C1_1643533F-R2	5' RACE: reverse primer for second round PCR	CTTTTCGGAAGCCGTTGTT
35	C1_2273315F-R1	5' RACE: reverse primer for first round PCR	GGCTATCATCCGGAAAAAGG
36	C1_2273315F-R2	5' RACE: reverse primer for second round PCR	AAGGACGGCCAGCGTAAT
37	C2_1966629F-R1	5' RACE: reverse primer for first round PCR	GCTCGCCACTCATGGAGTAA
38	C2_1966629F-R2	5' RACE: reverse primer for second round PCR	GCCACTCATGGAGTAACGAA
39	pAT-55271R-R1	5' RACE: reverse primer for first round PCR	CGATGAGAAACCGGAAAAAC
40	pAT-55271R-R2	5' RACE: reverse primer for second round PCR	AGAAACCGGAAAAACCATCG
41	pAt157836F-R1	5' RACE: reverse primer for first round PCR	TGAAGCGTCTGACAATCACC
42	pAt157836F-R2	5' RACE: reverse primer for second round PCR	CGTCTGACAATCACCGTTTC
43	C1_2667196F-R1	5' RACE: reverse primer for first round PCR	CTGGGGGTAAAGGGTCAATG
44	C1_2667196F-R2	5' RACE: reverse primer for second round PCR	GGGGTAAAGGGTCAATGCAC
45	C2_1324083F-R1	5' RACE: reverse primer for first round PCR	GTTGATTGGTCCAAGCCTGT
46	C2_1324083F-R2	5' RACE: reverse primer for second round PCR	GATTGGTCCAAGCCTGTCAT
47	pTi_5440F-R1	5' RACE: reverse primer for first round PCR	GCAGTTTGTCATTTCGCCCTA
48	pTi_5440F-R2	5' RACE: reverse primer for second round PCR	GTCATTTCGCCCTACAATCACA
49	pTi_54770R-R1	5' RACE: reverse primer for first round PCR	TGGGTCAGAGCAGTTCAAGC
50	pTi_54770R-R2	5' RACE: reverse primer for second round PCR	TCAGAGCAGTTCAAGCAATCTG
51	pTi_82838F-R1	5' RACE: reverse primer for first round PCR	TCAGGTACGACGACTACTTCACTG
52	pTi_82838F-R2	5' RACE: reverse primer for second round PCR	CGACTACTTCACTGCCACCG
53	pTi_84241F-R1	5' RACE: reverse primer for first round PCR	CAGGCAATACGCCGAATC
54	pTi_84241F-R2	5' RACE: reverse primer for second round PCR	AATACGCCGAATCCCGTT
55	pTi_125132R-R1	5' RACE: reverse primer for first round PCR	CCGAACGCTATGTCGAGTCTG
56	pTi_125132R-R2	5' RACE: reverse primer for second round PCR	CGCTATGTCGAGTCTGCCAC
57	pTi_148374R-R1	5' RACE: reverse primer for first round PCR	GGCTGGGTGCGACCACAG
58	pTi_148374R-R2	5' RACE: reverse primer for second round PCR	CTGGGTGCGACCACAGCC
59	pTi_190683R-R1	5' RACE: reverse primer for first round PCR	CAAGGTTCTCCCACCCCAG
60	pTi_190683R-R2	5' RACE: reverse primer for second round PCR	CTCCCACCCCAGCCAGTG
61	pTi_201463R-R1	5' RACE: reverse primer for first round PCR	TTGACGGGCATCTACGATG
62	pTi_201463R-R2	5' RACE: reverse primer for second round PCR	CGGGCATCTACGATGACGC
63	pTi_206590R-R1	5' RACE: reverse primer for first round PCR	CGGACATGCTCGATTCTCAG

	Oligonucleotide name	Purpose	Sequence
64	pTi_206590R-R2	5' RACE: reverse primer for second round PCR	CTCGATTCTCAGTCAAGTCATGTT
65	E1	3' RACE: 3' RNA adapter	5'phosphate-UUCACUGUUCUUAGCGGCCGCAUGCUC-idT-3' (idT: 3' inverted deoxythymidine)
66	E1SP-R1	3' RACE: E1 specific primer for cDNA synthesis and first round PCR	CATGCGGCCGCTAAGAAC
67	E1SP-R2	3' RACE: E1 specific primer for second round PCR	GGCCGCTAAGAACAGTGAA
68	C1_813550R-F1	3' RACE: forward primer for first round PCR	GCTTTTAAGACTTTCAGATGTCG
69	C1_813550R-F2	3' RACE: forward primer for second round PCR	GCGTCCCCTGAAACAAGG
70	C1_110258F-F1	3' RACE: forward primer for first round PCR	TGGAACATCAGACCGTTGAA
71	C1_110258F-F2	3' RACE: forward primer for second round PCR	GGATGTACTGGCACTGACGA
72	C1_1643533F-F1	3' RACE: forward primer for first round PCR	GCCTGAAATACCGGAGTATGA
73	C1_1643533F-F2	3' RACE: forward primer for second round PCR	AACAACGGCTTCCGAAAAG
74	C1_2273315F-F1	3' RACE: forward primer for first round PCR	CGCGACTGCCGTTATCTCTA
75	C1_2273315F-F2	3' RACE: forward primer for second round PCR	GTCGGGCTGCTGATTGAC
76	C2_1966629F-F1	3' RACE: forward primer for first round PCR	CGGCGGTTTTCATTTCAG
77	C2_1966629F-F2	3' RACE: forward primer for second round PCR	CGGATCGTTCGTTACTCCAT
78	pAt55271R-F1	3' RACE: forward primer for first round PCR	GTGGCAGGGATGACGAAC
79	pAt55271R-F2	3' RACE: forward primer for second round PCR	GACGAACGCGATGGTTTT
80	pAt157836F-F1	3' RACE: forward primer for first round PCR	TACACGATTCACTCGGCAAA
81	pAt157836F-F2	3' RACE: forward primer for second round PCR	GCCGTAACCGTATCAAGTTCA
82	C1_2667196F-F1	3' RACE: forward primer for first round PCR	GCCTAGGCACCAAGATGACC
83	C1_2667196F-F2	3' RACE: forward primer for second round PCR	GGTGCATTGACCCTTTACCC
84	C2_1324083F-F1	3' RACE: forward primer for first round PCR	GTACCTCCCGCCCAGATAAT
85	C2_1324083F-F2	3' RACE: forward primer for second round PCR	ATGAGCAATCCCAAAACAGG
86	pTi_5440F-F1	3' RACE: forward primer for first round PCR	GCTGTGATTGTAGGGCGAAAT
87	pTi_5440F-F2	3' RACE: forward primer for second round PCR	TGATTGTAGGGCGAAATGACA
88	pTi_54770R-F1	3' RACE: forward primer for first round PCR	TGCTTGAACTGCTCTGACCC
89	pTi_54770R-F2	3' RACE: forward primer for second round PCR	GCTCTGACCCAAGCATCGC
90	pTi_82838F-F1	3' RACE: forward primer for first round PCR	CGGTGGCAGTGAAGTAGTCG
91	pTi_82838F-F2	3' RACE: forward primer for second round PCR	GAAGTAGTCGTCGTACCTGATCG
92	pTi_84241F-F1	3' RACE: forward primer for first round PCR	CGGCGTATTGCCTGATATG
93	pTi_84241F-F2	3' RACE: forward primer for second round PCR	ATTGCCTGATATGGCTGACC
94	pTi_125132R-F1	3' RACE: forward primer for first round PCR	GCTCGATATCCTGCCCGATC
95	pTi_125132R-F2	3' RACE: forward primer for second round PCR	TATCCTGCCCGATCTCCG

	Oligonucleotide name	Purpose	Sequence
96	pTi_148374R-F1	3' RACE: forward primer for first round PCR	CGGCTGTGGTCGCACCCA
97	pTi_148374R-F2	3' RACE: forward primer for second round PCR	TGTGGTCGCACCCAGCCC
98	pTi_190683R-F1	3' RACE: forward primer for first round PCR	CTGGCTGGGGTGGGAGAA
99	pTi_190683R-F2	3' RACE: forward primer for second round PCR	GGGGTGGGAGAACCTTGC
100	pTi_201463R-F1	3' RACE: forward primer for first round PCR	TAGGTCGGTGTTTCGTCGTC
101	pTi_201463R-F2	3' RACE: forward primer for second round PCR	CGGTGTTTCGTCGTCGGC
102	pTi_206590R-F1	3' RACE: forward primer for first round PCR	ACCTCAGACCGATTGCCTT
103	pTi_206590R-F2	3' RACE: forward primer for second round PCR	GACCGATTGCCTTGAGCG
104	atsD-UP-F1-SphI	Forward primer for the upstream flanking sequence of *atsD*	AGTCGCATGCGATATCGAGCCTCCTGCAAA
105	atsD-UP-R1-SacII	Reverse primer for the upstream flanking sequence of *atsD*	TACGCCGCGGGAGCGCAGGATGTAAGGTTC
106	atsD-DN-F1-SacII	Forward primer for the downstream flanking sequence of *atsD*	TACGCCGCGGATTCACTCGGCAAACCAAAA
107	atsD-DN-R1-EcoRI	Reverse primer for the downstream flanking sequence of *atsD*	ACTGGAATTCTGGTGCAGGTGTTACGGATA
108	PaadAT-F2	Forward primer for the selectable marker *aadA*	ATGCGGTACCATTATTCTTAAATTATTCAATTAGATA
109	TpsbANT-R2	Reverse primer for the selectable marker *aadA*	CCACCGCGGTGGAAATAAC
110	MCS-BE-F1	Forward strand of the multiple cloning sites for pTF505	GATCTGACTAAGCTTACGCGTCTGCAGCCGCGGTCGAC CCGGG
111	MCS-BE-R1	Reverse strand of the multiple cloning sites for pTF505	TTAAGGGCCCAGCTGGCGCCGACGTCTGCGCATTCGAA TCAGT
112	pTF505F1	Sequencing primer for pTF505	GCCTCTTATCCATTCTCATTGAA
113	pTF505R1	Sequencing primer for pTF505	ACGGCTGCACTGAACGTC
114	AtuPrrnC-F1-KpnI	Forward PCR primer for the PrrnC promoter	AGTCGGTACCTACGAGCCCGAAAATCGTTA
115	AtuPrrnC1-R-BglII	Reverse PCR primer for the PrrnC promoter	AGTCAGATCTGTGAGCGGACTTATAAGAGAA
116	TpsbANT-F-EcoRI	Forward PCR primer for the terminator TspbaNT	AGTCGAATTCCTCGACAGATCCTGGCCTA
117	TpsbANT-R-SphI	Reverse PCR primer for the terminator TspbaNT	AGTCGCATGCTGGAAATAACTTCGTATAATGTATGCT
118	C58avb10R-H3F1	Forward PCR primer for pTi_191667R overexpression construct without native promoter	ATGCAAGCTTATAATCGTCCCCTCCGTCAC
119	C58avb10R-BstR1	Reverse PCR primer for pTi_191667R overexpression construct without native promoter	ATGCCCACCGCGGTGGGGGGCTCAAAAGTTGATCGT
120	C58avb10R-H3F2	Forward PCR primer for pTi_191667R overexpression construct with native promoter	ATGCAAGCTTACGAACATCCCAGGGTAACA
121	C58avb10R-BstR2	Reverse PCR primer for pTi_191667R overexpression construct with native promoter	ATGCCCACCGCGGTGGTGGTCTCCGACACACATCAC
122	C58anti-avb10R-H3F1	Forward PCR primer for anti-pTi_191667R overexpression construct without native promoter	ATGCAAGCTTGGGGCTCAAAAGTTGATCGT
123	C58anti-avb10R-BstR1	Reverse PCR primer for anti-pTi_191667R overexpression construct without native promoter	ATGCCCACCGCGGTGGATAATCGTCCCCTCCGTCAC
124	C58anti-avb10R-H3F2	Forward PCR primer for anti-pTi_191667R overexpression construct with native promoter	ATGCAAGCTTTGGTCTCCGACACACATCAC
125	C58anti-avb10R-BstR2	Reverse PCR primer for anti-pTi_191667R overexpression construct with native promoter	ATGCCCACCGCGGTGGACGAACATCCCAGGGTAACA
126	aatsDR-F1	Forward PCR primer for pAt_157836F overexpression construct	ATCCAAGCTTAAGCAAAGAATGCTGTGACG
127	aatsDR-R1	Reverse PCR primer for pAt_157836F overexpression construct	ATGCTGCAGGCATACCCCGTGAGAAAGG

	Oligonucleotide name	Purpose	Sequence
128	anti-atsDR-F1	Forward PCR primer for anti-pAt_157836F overexpression construct	ATCCAATCTTGCATACCCCGTGAGAAAGG
129	anti-atsDR-R1	Reverse PCR primer for anti-pAt_157836F overexpression construct	ATGCTGCAGAAGCAAAGAATGCTGTGACG
130	atsD-OE-F1	Forward PCR primer for atsD overexpression construct	ATCCAAGCTTGGAGGTCGAAATGATCTACATTG
131	atsD-OE-R1	Reverse PCR primer for atsD overexpression construct	ATCGCTGCAGCACGCTGCTGCAAGACAT
