# Supplementary material for: A Genome-Wide Survey of Highly Expressed Non-Coding RNAs and Biological Validation of Selected Candidates in Agrobacterium tumefaciens
Source: PLoS One. 2013 Aug 8;8(8):e70720. doi: 10.1371/journal.pone.0070720 (PMC3738593; doi:10.1371/journal.pone.0070720)
Supplement: File S2 — Figure S1, Effects of primary transcript enrichment by terminator 5′-phosphate-dependent exonuclease; Figure S2, 5′ and 3′ RACE for ncRNAs on Ti plasmid; Figure S3, Expression profiling of virD4* internal transcript with primary transcript enrichment (+TEX); Figure S4, Expression profiling of C3 and Ti2; Figure S5, Effects of two antisense RNAs (pTi_191667R and pAt_157836F) on Agrobacterium virulence; Figure S6, Map of the expression vector pTF505. (PDF) Chi [file pone.0070720.s002.pdf]

SUPPORTING INFORMATION FILE S2

A Genome-Wide Survey of Highly Expressed Non-Coding RNAs and Biological Validation of Selected Candidates in *Agrobacterium tumefaciens*

Keunsub Lee[1,2], Xiaoqiu Huang[3], Chichun Yang[1,2], Danny Lee[4], Vincent Ho[4], Kan Nobuta[4], Jian-Bing Fan[4], Kan Wang[1,2,*]

This file contains:

Figure S1. Effects of primary transcript enrichment by terminator 5'-phosphate-dependent exonuclease

Figure S2. 5' and 3' RACE for ncRNAs on Ti plasmid

Figure S3. Expression profiling of virD4* internal transcript with primary transcript enrichment (+TEX)

Figure S4. Expression profiling of C3 and Ti2.

Figure S5. Effects of two antisense RNAs (pTi_191667R and pAt_157836F) on *Agrobacterium* virulence

Figure S6. Map of the expression vector pTF505

References

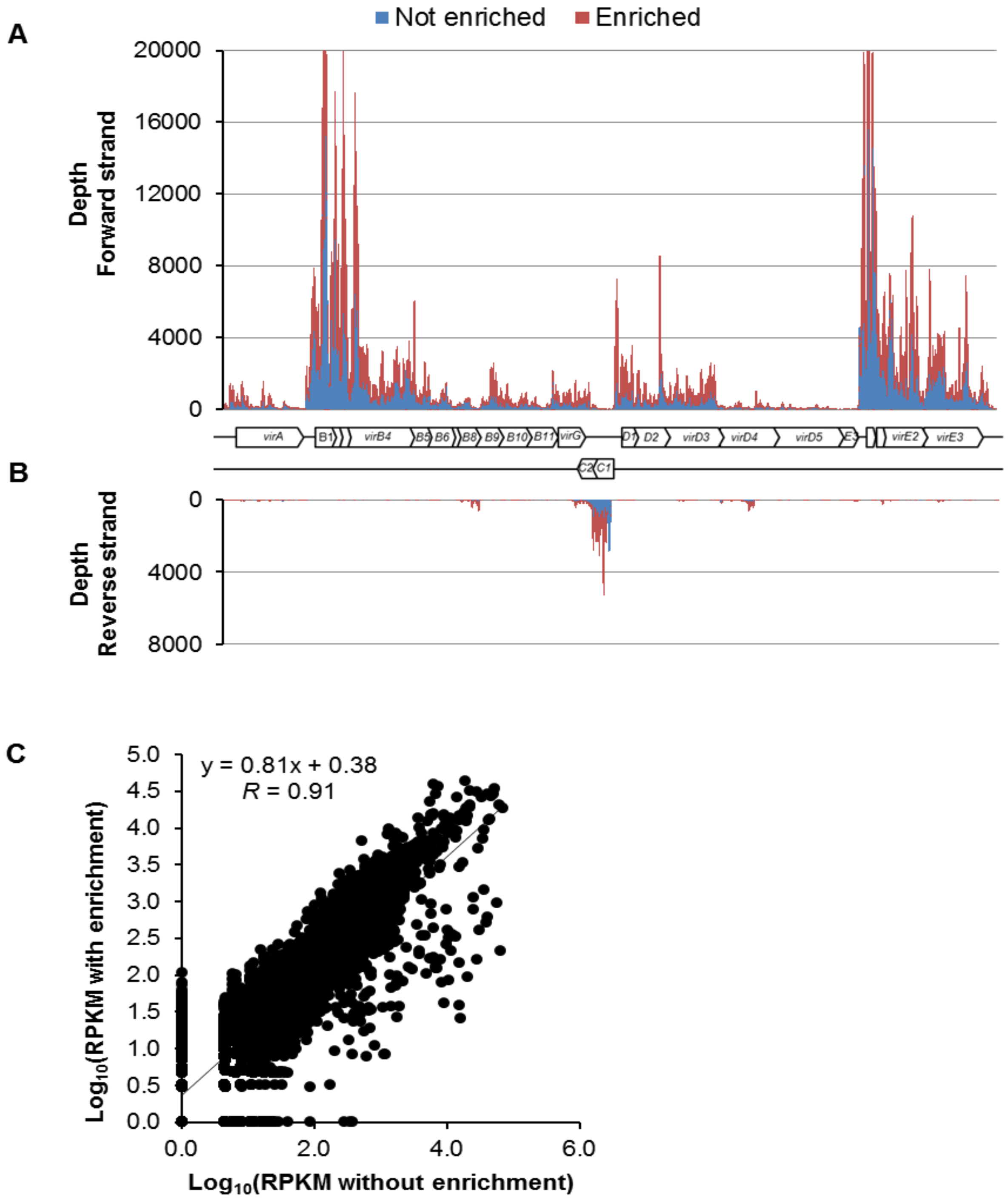

Figure S1. Effects of primary transcript enrichment by terminator 5'-phosphate-dependent exonuclease. Primary transcript enrichment by thermiator 5'-phosphate-dependent exonuclease (+TEX) improves the overall genome coverage without significant bias in transcript quantification. Depth of coverage at each nucleotide position was plotted with respect to the

annotated *vir* genes on Ti plasmid: A) forward strand and B) reverse strand. At 92.8% (28303/30505) of the nucleotide positions from 180590 to 211094 had higher depth of coverage with TEX enrichment than did without enrichment. C) Log-transformed RPKMs of 5,355 protein-coding genes with and without primary transcript enrichment were plotted. There was a very high correlation, r = 0.91, suggesting that primary transcript enrichment did not have systemic bias on gene expressions. For those genes with very low coverage without enrichment became detectable with enrichment. Among the 5432 protein-coding genes, 3411 ~ 3842 genes were detected (RPKM > 0) without TEX treatment (YEP-L, 3603; YEP-S, 3487; AB, 3842; IND, 3411), while 3957 ~ 4361 genes were detected with TEX enrichment (YEP-L, 3957; YEP-S, 3959; AB, 4361; IND, 4156).

ncRNA tag	5' end	5' RACE<br>TAP* + - +<br>RT† + + -	3' end	3' RACE<br>RT + -	antisense to
pTi_5440F	5439	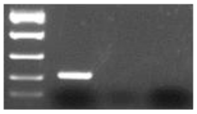	5747	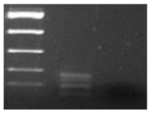	
pTi_54770R		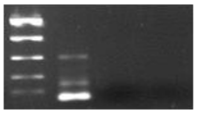	54062	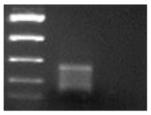	*Atu6044* (*repB*)
pTi_82838F	82933	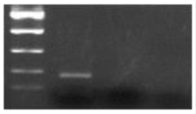	83163	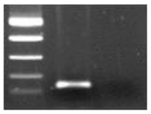	*Atu6069* (*rbsA*)
pTi_84241F	84210	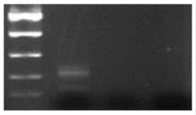	84492	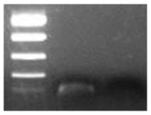	*Atu6071* (*aiiB*)
pTi_125132R	125132	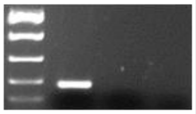	124994	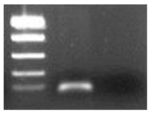	*Atu6101*
pTi_148374R	no amplification		no amplification		*Atu6129* (*traB*)
pTi_190683R	190702	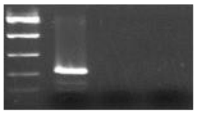	190311	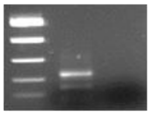	*Atu6175* (*virB9*)
pTi_201463R	201398	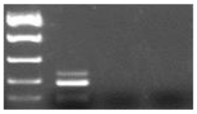	201234	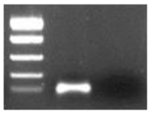	*Atu6184* (*virD4*)
pTi_206590R	no amplification		no amplification		*Atu6190* (*virE2*)

Figure S2. 5' and 3' RACE for ncRNAs on Ti plasmid. Nine ncRNAs on Ti plasmid were selected for validation with RACE. Seven of them were detected with 5' and 3' RACE, including *cis*-antisense RNAs for *virB9* and *virD4*. E Gel® Low Range Quantitative DNA Ladder (Invitrogen, Inc.) was run along with the PCR products and represent 2 kb, 800 bp, 400 bp, 200 bp, and 100 bp. *TAP: tobacco acid pyrophosphatase. †RT: reverse transcriptase.

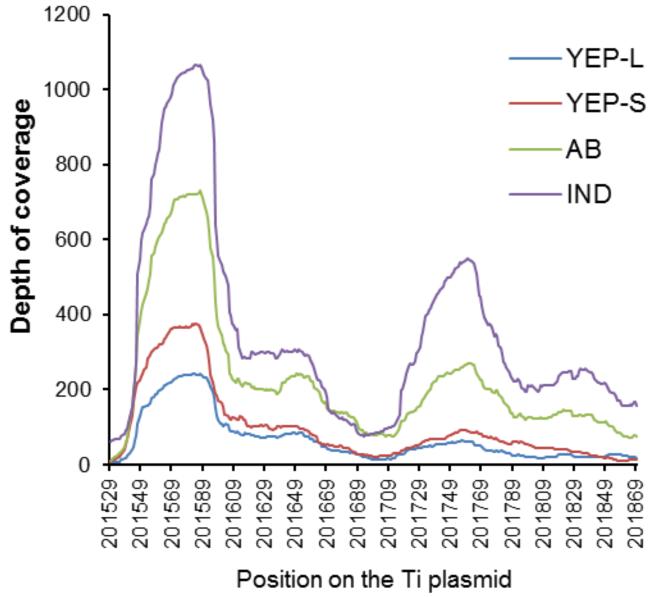

Figure S3. Expression profiling of virD4* internal transcript with primary transcript enrichment (+TEX). An internal transcript from *virD4* was expressed without *vir* gene induction by AS. Depth of coverage for each nucleotide position from 201529 to 201869 on Ti plasmid was plotted. RPKM (+TEX): YEP-L, 267; YEP-S, 380; AB, 526; IND, 738.

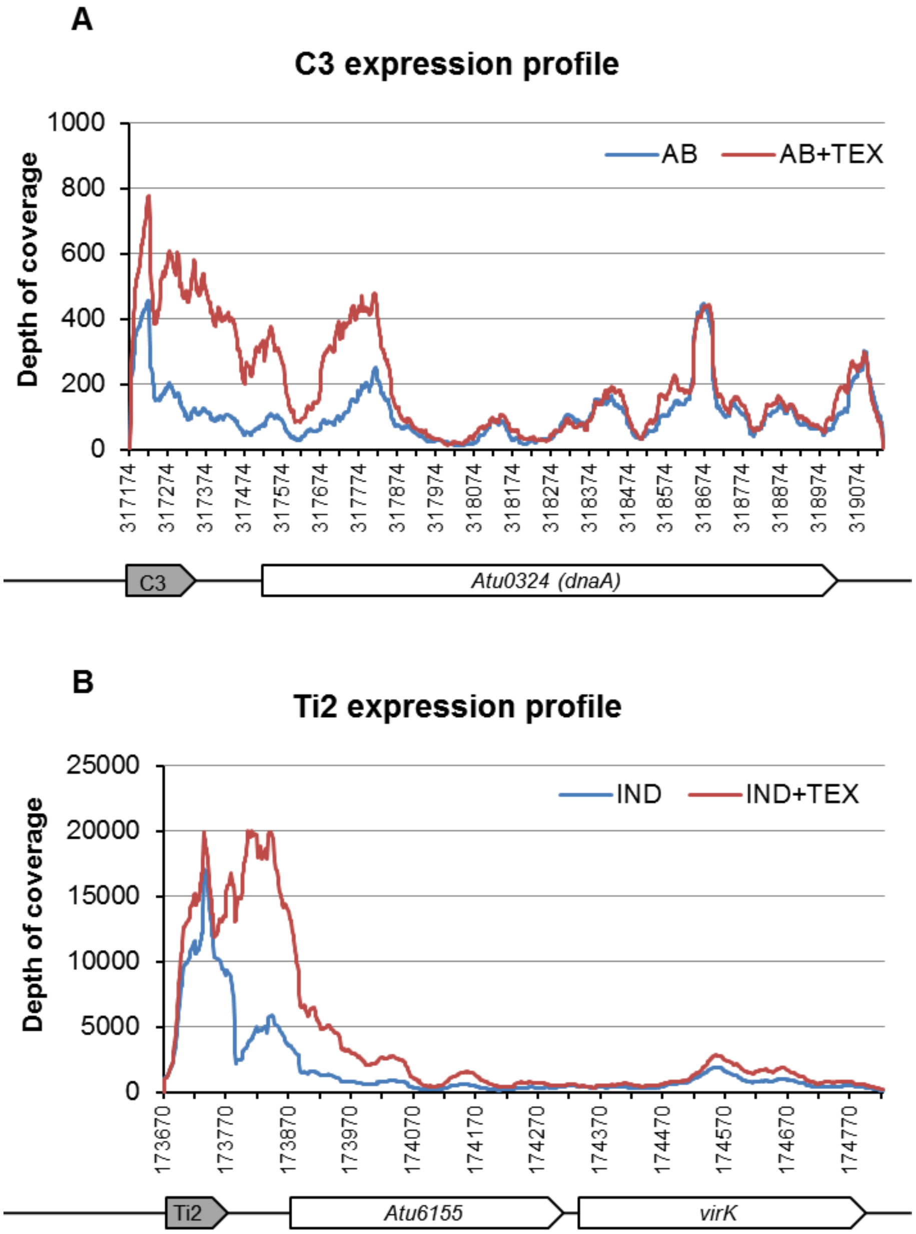

Figure S4. Expression profiling of C3 and Ti2**.** Our depth of coverage data suggested that two sRNAs identified by Wilms et al. (2012), C3 and Ti2, were likely part of 5’ UTRs of *dnaA* (A) and *Atu6155* (B).

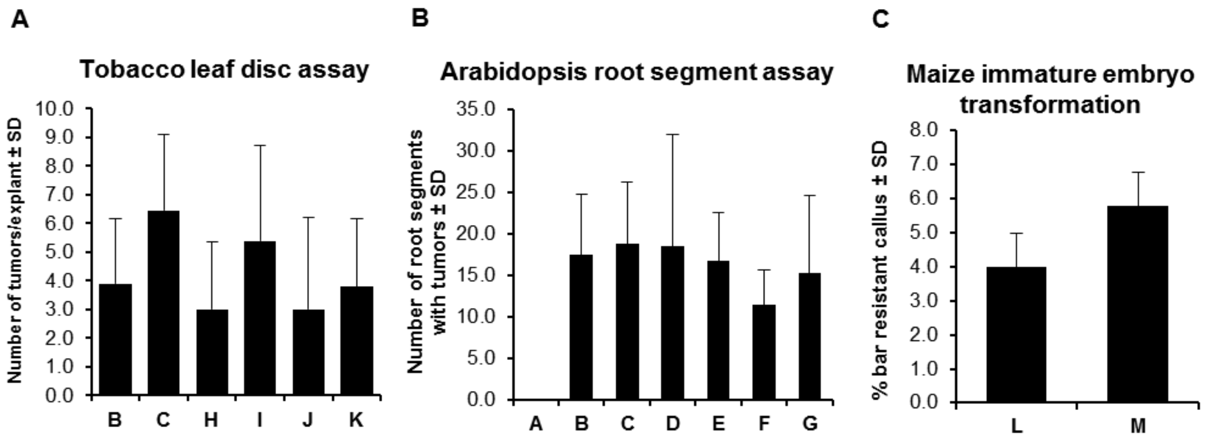

Strain Code	Strain name	Strain description	Tobacco leaf disc	Arabidopsis root segment	Maize immature embryo
A	A136	Avirulent *Agrobacterium* strain, negative control		x	
B	C58 WT	Virulent *Agrobacterium* strain C58	x	x	
C	C58 WT+EV	C58 carrying an empty plasmid vector control	x	x	
D	Δ*atsD*	C58 knockout mutant of *atsD* and *cis*-antisense RNA pAt_157840F		x	
E	OE: pAt_157840F	C58 carrying an overexpression construct of pAt_157840F		x	
F	OE: anti-pAt_157840F	C58 carrying an overexpression construct of the antisense sequence of pAt_157840F		x	
G	OE: *atsD*	C58 carrying an overexpression construct of *atsD*		x	
H	OE: pTi_191635R-S	C58 carrying an overexpression construct of pTi_191635R without putative native promoter	x		
I	OE: pTi_191635R-L	C58 carrying an overexpression construct of pTi_191635R with putative native promoter	x		
J	OE: anti-pTi_191635R-S	C58 carrying an overexpression construct of the antisense sequence of pTi_191635R without putative native promoter	x		
K	OE: anti-pTi_191635R-L	C58 carrying an overexpression construct of the antisense sequence of pTi_191635R with putative native promoter	x		
L	A11S10	Binary vector construct pTF101.1 (CaMV 35S promoter drives herbicide resistance bar gene, Paz et al. 2004) in *Agrobacterium* strain EHA105			x
M	A11S11	A11S10 knockout mutant of *atsD* and *cis*-antisense RNA pAt_157840F			x

Figure S5. Effects of two antisense RNAs (pTi_191667R and pAt_157836F) on *Agrobacterium* virulence. A) Tobacco leaf disc assay was performed using *A. tumefaciens* C58 strains overexpressing a *cis*-antisense RNA (H and I: pTi_191667R, antisense to *virB10*) or its complementary sequence (J and K: anti-pTi_191667R) with/without its native promoter (L/S). Eighteen leaf discs were used for each strain and the numbers of tumors on each leaf disc were recorded three weeks after inoculation. The mean numbers of tumors per explant ± standard deviation were shown. There was no significant difference between treatments (Student's *t*-test, $P > 0.05$). B) Arabidopsis root segment transformation assay was conducted using a knockout strain of *atsD* and *cis*-antisense RNA (D) and overexpression strains (E ~ H). Sixty root segments were used for each strain and the numbers of root segments with tumors were recorded three weeks after inoculation. Experiments were repeated 2 – 6 times for each strain. The mean

numbers of root segments with tumors ± standard deviation were shown. No significant differences among treatments were found (Student's *t*-test, $P > 0.05$). C) Maize immature embryo transformation was conducted using disarmed *Agrobacterium* strain EHA105 (L) and *atsD* knockout strain (M) carrying a binary vector pTF101.1 (Paz *et al.*, 2004). For each infection experiment, around 200 maize immature embryos were used for both strains. A total of three independent infection experiments were performed for this side-by-side comparison. Mean % maize embryos with callus ± standard deviation from three independent experiments were shown. There was marginally significant difference between the wild type (L) and knockout mutant (M) (paired-sample *t*-test, $P = 0.017$).

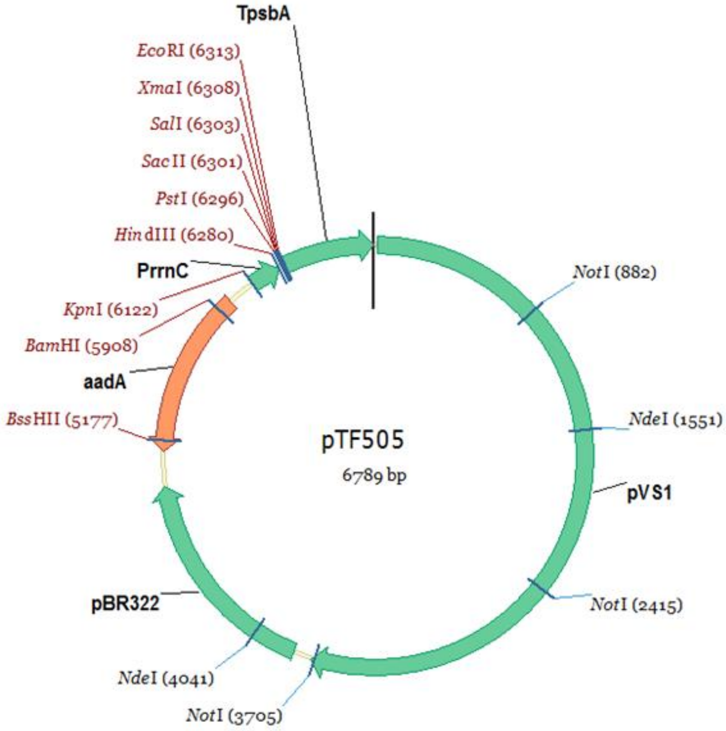

Figure S6. Map of the expression vector pTF505. P*rrnC*, *A. tumefaciens rrnC* promoter for constitutive gene expression; TpsbA, transcription terminator; *aadA*, encoding aminoglycoside 3' adenyltransferase for resistance to antibiotics spectinomycin and streptomycin; pVS1, replication origin for *Agrobacterium*; pBR322, replication origin for *E. coli*.

References

Paz, M., H. Shou, Z. Guo, Z. Zhang, A. Banerjee & K. Wang, (2004) Assessment of conditions affecting *Agrobacterium* -mediated soybean transformation using the cotyledonary node explant. *Euphytica* **136**: 167-179.

Wilms I, Overlöper A, Nowrousian M, Sharma CM, Narberhaus F (2012) Deep sequencing uncovers numerous small RNAs on all four replicons of the plant pathogen *Agrobacterium tumefaciens*. RNA Biology 9: 446-457.
